# Structurally Diverse Polymethylated Phloroglucinol Meroterpenoids from *Baeckea frutescens*

**DOI:** 10.1007/s13659-018-0189-3

**Published:** 2018-10-29

**Authors:** Yin-E Zhi, Xu-Jie Qi, Hui Liu, Yuan Zeng, Wei Ni, Li He, Zu-Ding Wang, Hai-Yang Liu

**Affiliations:** 1 State Key Laboratory of Phytochemistry and Plant Resources in West China, Kunming Institute of Botany, Chinese Academy of Sciences, and Yunnan Key Laboratory of Medicinal Chemistry, Kunming, 650201 China; 2 Department of Dermatology, The First Affiliated Hospital of Kunming Medical University, Kunming, 650032 China; 3 Kunming Botanee Bio-Technique Co. Ltd, Kunming, 650106 China

**Keywords:** *Baeckea frutescens*, Myrtaceae, Polymethylated phloroglucinol meroterpenoids, Anti-inflammatory activity

## Abstract

**Electronic supplementary material:**

The online version of this article (10.1007/s13659-018-0189-3) contains supplementary material, which is available to authorized users.

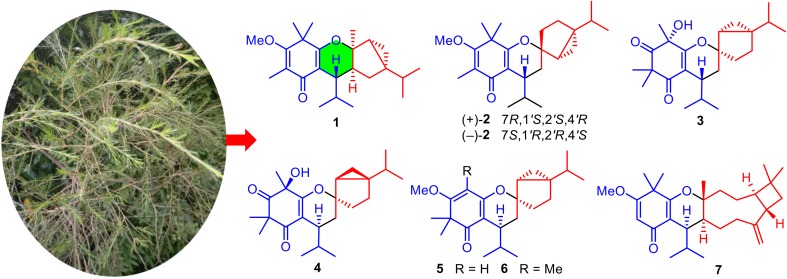

## Introduction

**Fig. 1:**
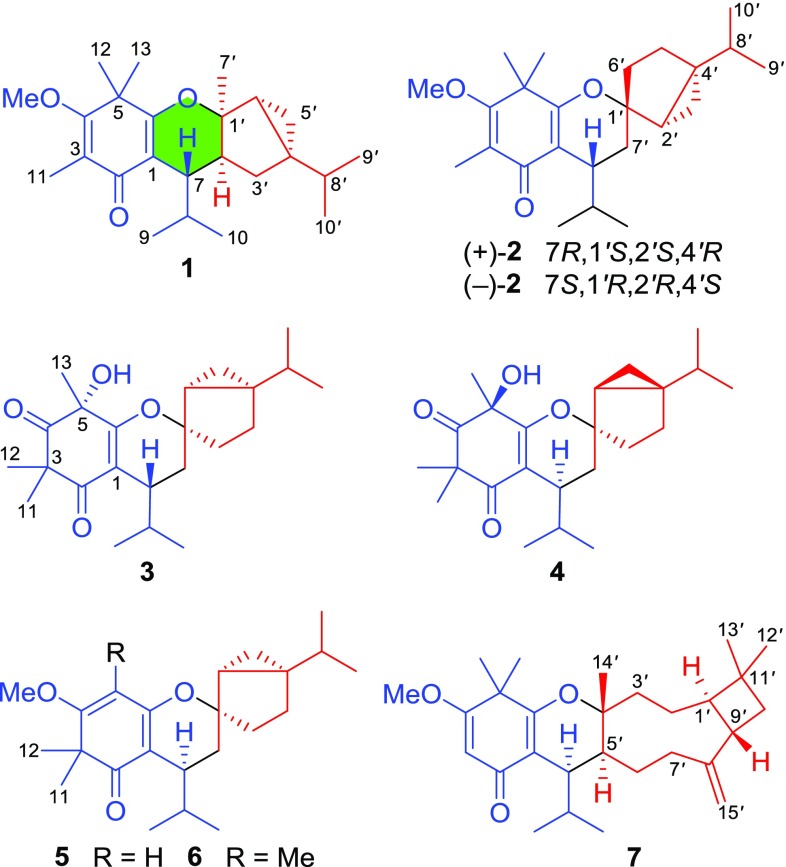
Chemical structures of **1**–**7**

Species of Myrtaceae family are known to be a rich source of structurally interesting and bioactive phloroglucinol derivatives [1–7]. *Baeckea frutescens*, widely distributed in south China, has been used as a folk herbal medicine for the treatment of fever, rheumatism, and snake bites [8]. Our previous work reported 12 new polymethylated phloroglucinol meroterpenoids (PPMs) with significant cytotoxic and anti-inflammatory effects, baeckfrutones A–L, from the twigs and leaves of *Baeckea frutescens* [9]. Further chemical examination by an HPLC–UV guided method led to the isolation of seven new PPMs, baeckfrutones M–S (Fig. 1), from the twigs and leaves of title species. All isolates were evaluated for their cytotoxic activities against four human cancer cell lines (HCT116, Hela, DU145, and A549) and anti-inflammatory effects on NO levels in LPS-stimulated RAW 264.7 macrophages. Herein, we report the isolation, structural elucidation, and bioactivities of these isolates.

## Results and Discussion

**Fig. 2:**
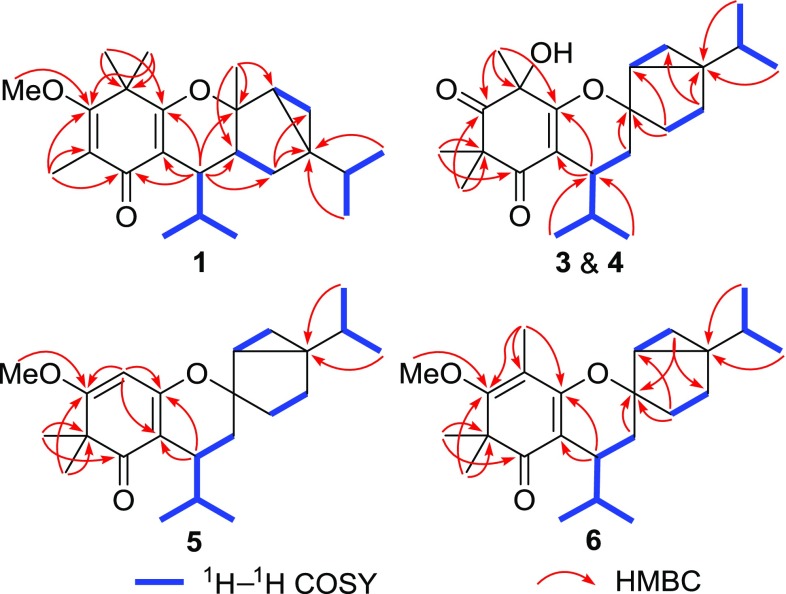
Key ^1^H–^1^H COSY and HMBC correlations of **1** and **3**–**6**

Baeckfrutone M (**1**) was isolated as a colorless gum. Its molecular formula, C_24_H_36_O_3_, was determined based on the HRESIMS *m*/*z* 395.2567 [M + Na]^+^ (calcd for 395.2562), requiring seven indices of hydrogen deficiency (IHDs). The ^1^H NMR spectrum (Table 1) of **1** showed signals attributable to eight methyls [*δ*_H_ 1.90, 1.48, 1.34, 1.31 (each s); 0.99 (d, *J* = 6.6 Hz), 0.81 (d, *J* = 6.8 Hz), 0.76 (d, *J* = 6.6 Hz), 0.74 (d, *J* = 6.8 Hz)], a methoxy group [*δ*_H_ 3.85 (s)], and two upfield protons [*δ*_H_ 0.51 (dd, *J* = 8.4, 5.1 Hz), 0.34 (dd, *J* = 5.1, 3.5 Hz)] for a cyclopropane methylene. The ^13^C NMR data (Table 1) of **1** revealed the presence of 24 carbons corresponding to one ketone carbonyl, four olefinic (two oxygenated ones), three quaternary (an oxygenated one), five methine, two methylene, one methoxyl, and eight methyl carbons. The aforementioned one ketone carbonyl and two double bond functionalities accounted for three of seven IHDs, thus suggesting **1** to be tetracyclic. Combined with the fragment of H-7–H-8–Me-9 and Me-10 as disclosed by the ^1^H–^1^H COSY spectrum, the HMBC correlations from H-7 to C-1/C-2/C-6, from Me-11 to C-2/C-3/C-4, from both Me-12 and Me-13 to C-4/C-5/C-6, and from *δ*_H_ 3.85 to C-4 revealed the presence of an enone-type isobutyrylphloroglucinol moiety (Fig. 2). Similarly, the existence of a thujene unit was verified by the HMBC correlations from H_2_-3*′* to C-4*′*/C-5*′*, from Me-7*′* to C-1*′*/C-2*′*/C-6*′*, and from Me-9*′*/Me-10*′* to C-4*′*, as well as the ^1^H − ^1^H COSY correlations (Fig. 2). Moreover, the HMBC correlations from H-7 to C-1*′*/C-2*′*/C-3*′* revealed a dihydropyran ring system [10] connecting the phloroglucinol and monoterpenoid moieties.

**Table 1:** ^1^H (500 MHz) and ^13^C (125 MHz) NMR data of **1** in CDCl_3_

No.	*δ*_C_	*δ*_H_ (mult., *J* in Hz)	No.	*δ*_C_	*δ*_H_ (mult., *J* in Hz)
1	109.2		1*′*	87.7	
2	188.7		2*′*	39.5	2.04 ddd (9.8, 7.7, 1.2)
3	117.2		3*′*a	36.5	1.77 dd (12.5, 7.7)
4	171.7		3*′*b		1.26 dd (12.5, 9.8)
5	42.6		4*′*	33.2	
6	168.5		5*′*a	14.3	0.51 dd (8.4, 5.1)
7	37.2	2.45 brdd (9.5, 1.2)	5*′*b		0.34 dd (5.1, 3.5)
8	31.9	1.66 m	6*′*	36.0	1.31 m
9	21.8	0.99 d (6.6)	7*′*	24.8	1.48 s
10	21.5	0.76 d (6.6)	8*′*	31.8	1.32 hept (6.8)
11	10.1	1.90 s	9*′*	19.5	0.74 d (6.8)
12	24.1	1.34 s	10*′*	20.0	0.81 d (6.8)
13	23.6	1.31 s	OMe-4	61.7	3.85 s

**Fig. 3:**
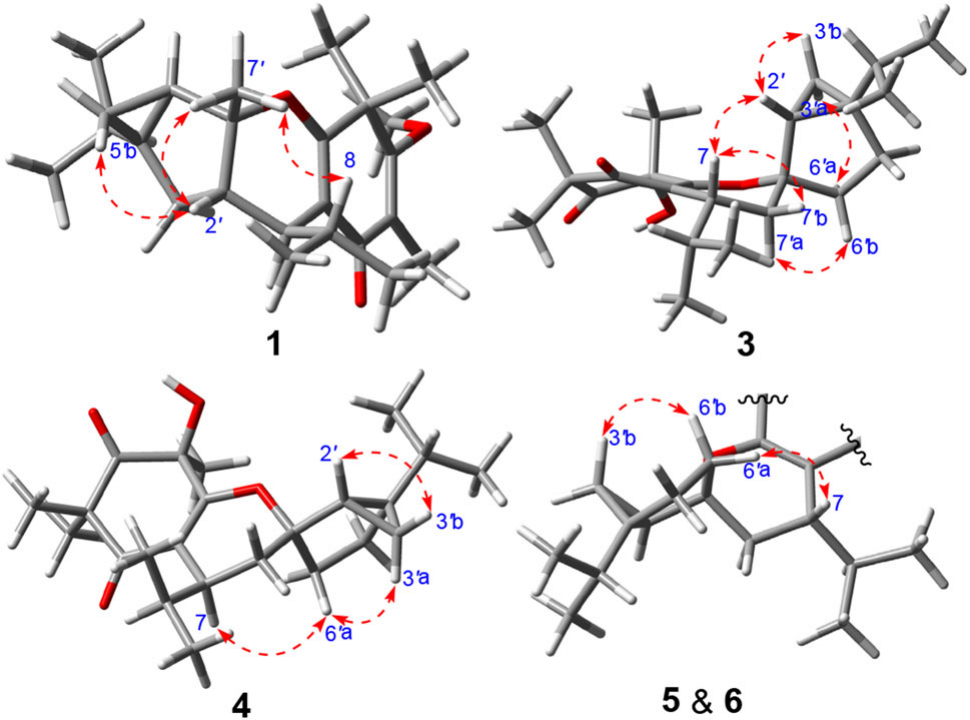
Key ROESY correlations of **1** and **3**–**5**

**Fig. 4:**
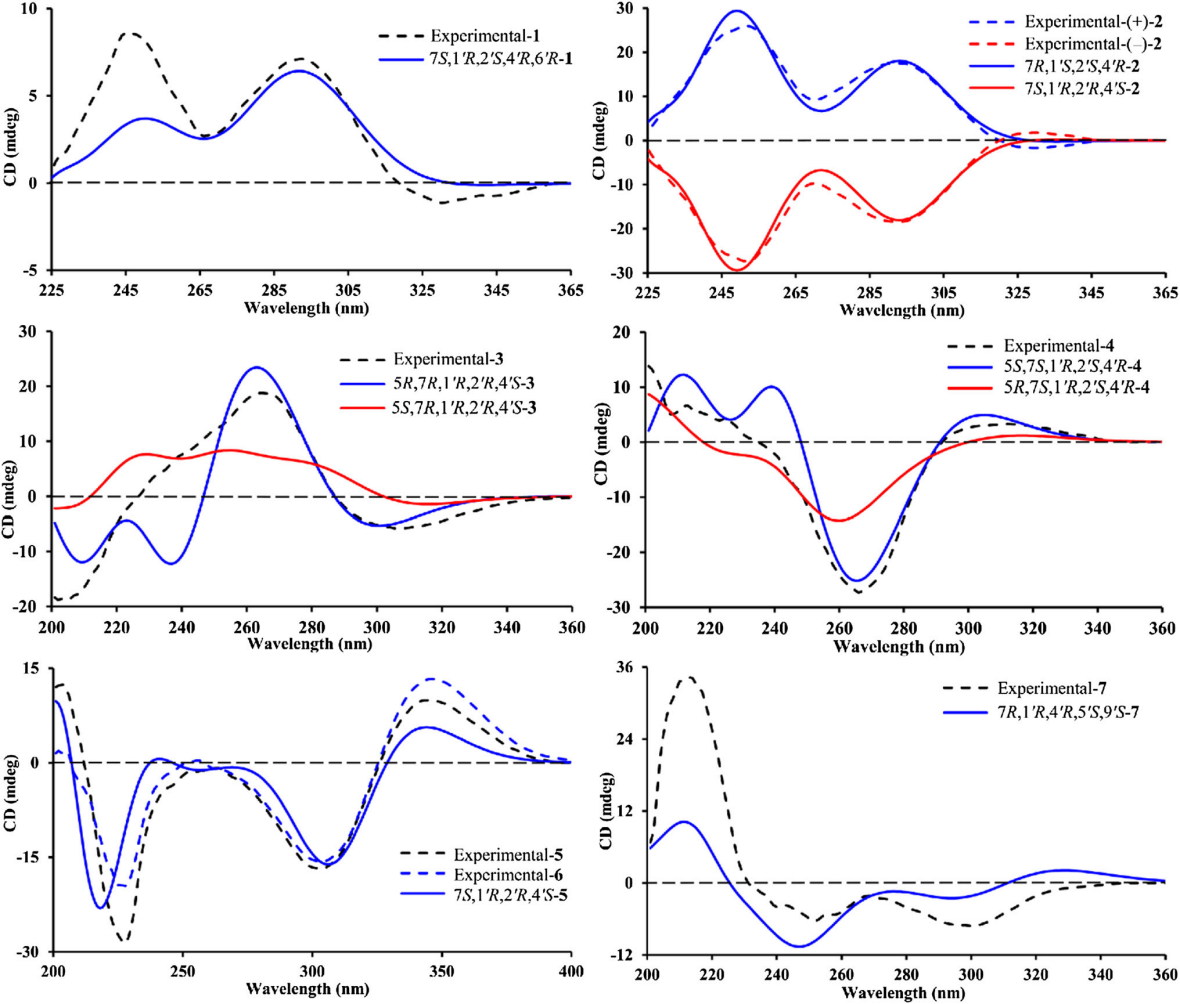
Calculated and experimental ECD data of **1**–**7**

The relative configuration of **1** was determined by the analysis of ROESY data (Fig. 3). The ROESY correlations of H-8 with Me-7*′*, of Me-7*′* with H-2*′*, and of H-2*′* with H-5*′*b suggested that these protons were co-facial and arbitrarily designated as *α*-oriented. Finally, the absolute configuration of **1** was assigned as 7*S*,1*′R*,2*′S*,4*′R*,6*′R* by the calculated ECD method (Fig. 4) using a time-dependent density functional theory (TDDFT) method at the B3LYP/6-311 ++G(2d,p) level.

Baeckfrutone N (**2**) shared the same molecular formula of C_24_H_36_O_3_ as **1** by the HRESIMS *m*/*z* 395.2568 [M + Na]^+^ (calcd for 395.2562). It possessed the same planar structure as beckfrutone B [9] based on the analysis of its 2D NMR data. Although the correlation of H-7 with H-6*′*a in ROESY spectrum was observed for **2**, the ROESY correlation of H-6*′*b with H-3*′*a indicated that the cyclopropane moiety in the sabinene unit was *α*-oriented. PPM **2** was proved to be a racemic mixture by the chiral HPLC analysis. The absolute configurations of (+)-**2** and (−)-**2**, 7*R*,1*′S*,2*′S*,4*′R* and 7*S*,1*′R*,2*′R*,4*′S*, were subsequently determined by further chiral HPLC separation and calculated ECD spectra (Fig. 4).

Baeckfrutone O (**3**) possessed the molecular formula of C_23_H_34_O_4_ with seven IHDs established by the HRESIMS *m*/*z* 397.2358 [M + Na]^+^ (calcd for 397.2355). The ^1^H NMR data (Table 2) of **3** displayed seven methyls [*δ*_H_ 1.59, 1.43, 1.32 (each s); 0.96 (d, *J* = 7.0 Hz), 0.92 (d, *J* = 6.9 Hz), 0.90 (d, *J* = 6.9 Hz), 0.58 (d, *J* = 7.0 Hz)]. The ^13^C and DEPT NMR data (Table 3) of **3** disclosed 23 carbon resonances, including two ketone carbonyl, six quaternary (two oxygenated and two olefinic ones), four methine, four methylene, and seven methyl carbons. The existence of a sabinene unit was further confirmed by HMBC correlations from H_2_-5*′* to C-3*′*/C-4*′*, from H_2_-6*′* to C-1*′*/C-2*′*, and from Me-9*′*/Me-10*′* to C-4′, as well as the ^1^H–^1^H COSY correlations (Fig. 2). The presence of an enone-type isobutyrylphloroglucinol moiety with a rare hydroxy group was inferred by the HMBC correlations from H-7 to C-1/C-6, from Me-9/Me-10 to C-7/C-8, from Me-11/Me-12 to C-2/C-3/C-4, and from Me-13 to C-4/C-5/C-6 (Fig. 2). Moreover, the HMBC correlation from H_2_-7*′* to C-1*′* and the partial structure of H_2_-7*′*–H-7 as revealed by the ^1^H–^1^H COSY correlations indicated that phloroglucinal was coupled with sabinene via a C-7–C-7*′* bond.

**Table 2:** ^1^H (500 MHz) NMR data of **2**–**6** in CDCl_3_

No.	**2**	**3**	**4**	**5**	**6**
5				5.07 s	
7	2.71 ddd (11.8, 7.7, 4.2)	2.95 ddd (11.4, 7.0, 3.8)	2.74 td (9.5, 4.0)	2.68 ddd (11.3, 7.6, 4.1)	2.59 ddd (11.2, 6.8, 4.6)
8	2.76 m	2.49 m	2.67 m	2.92 m	2.84 m
9	0.91 d (7.1)	0.96 d (7.0)	0.98 d (7.0)	0.93 d (7.0)	0.90 d (7.0)
10	0.54 d (6.9)	0.58 d (7.0)	0.70 d (6.9)	0.61 d (7.0)	0.62 d (7.0)
11	1.81 s	1.43 s	1.41 s	1.26 s	1.26 s
12	1.29 s	1.32 s	1.34 s	1.23 s	1.22 s
13	1.19 s	1.59 s	1.68 s		1.83 s
2*′*	1.08 dd (7.8, 3.3)	1.26 dd (8.0, 3.6)	1.17 dd (8.0, 3.4)	1.13 m	1.22 overlapped
3*′*a	0.75 dd (5.2, 3.3)	0.86 dd (5.0, 3.6)	0.91 dd (5.1, 3.7)	0.86 dd (5.3, 3.6)	0.48 dd (8.4, 5.4)
3*′*b	0.44 dd (7.8, 5.2)	0.40 dd (8.0, 5.0)	0.55 dd (8.0, 5.1)	0.52 dd (7.7, 5.3)	0.35 dd (5.4, 3.7)
5*′*a	1.60 2H m	1.74 2H m	1.71 m	1.66 2H m	1.82 overlapped
5*′*b			1.65 m		1.54 brdd (12.2, 7.9)
6*′*a	1.71 br dd (12.8, 6.6)	1.90 m	1.74 m	1.70 m	1.66 m
6*′*b	0.96 q-like (11.3)	1.73 m	1.19 m	1.14 overlapped	1.04 ddd (11.4, 6.8, 3.4)
7*′*a	1.68 2H m	1.84 brd (7.2)	1.79 2H dd (9.4, 0.7)	1.79 dd (13.3, 7.6)	1.76 brd (11.1)
7*′*b		1.72 brd (6.7)		1.14 dd (7.6, 3.7)	1.65 m
8*′*	1.34 hept (6.9)	1.30 hept (6.9)	1.40 hept (6.9)	1.39 hept (6.9)	1.40 hept (6.9)
9*′*	0.89 d (6.9)	0.92 d (6.9)	0.95 d (6.9)	0.95 d (6.9)	1.02 d (6.9)
10*′*	0.84 d (6.9)	0.90 d (6.9)	0.90 d (6.9)	0.90 d (6.9)	0.97 d (6.9)
OMe-4	3.78 s			3.65 s	3.77 s

**Table 3:** ^13^C (125 MHz) NMR data of **2**–**6** in CDCl_3_

No.	**2**	**3**	**4**	**5**	**6**
1	111.7	114.5	113.1	105.8	109.2
2	187.8	196.9	196.0	199.9	200.2
3	117.4	53.0	56.4	48.1	49.3
4	171.4	211.8	210.4	172.3	167.4
5	42.5	75.0	73.7	91.0	111.9
6	171.6	167.5	168.6	168.5	166.6
7	33.0	33.9	33.7	32.8	34.0
8	26.3	26.1	26.6	25.6	26.2
9	20.4	20.5	20.5	20.4	20.6
10	15.3	15.5	15.7	15.4	15.9
11	9.6	23.8	26.7	22.7	22.2
12	23.6	23.9	20.6	26.4	25.8
13	23.6	28.3	27.8		9.9
1*′*	86.5	89.2	88.6	87.3	89.7
2*′*	31.5	26.1	31.3	31.7	32.0
3*′*	11.3	12.0	11.8	11.5	13.0
4*′*	32.6	34.1	33.1	32.7	34.2
5*′*	25.1	24.3	25.2	25.2	24.8
6*′*	30.1	34.0	29.8	29.9	31.3
7*′*	30.4	30.9	30.1	29.7	28.8
8*′*	32.3	32.6	32.3	32.4	32.5
9*′*	19.5	19.5	19.6	19.6	19.5
10*′*	19.5	19.5	19.5	19.5	20.2
OMe-4	61.5			55.5	61.7

The relative configuration of **3** was assigned by a ROESY experiment. As depicted in Fig. 3, the ROESY correlations of H-7 with H-2*′*/H-7*′*b and of H-2*′* with H-3*′*b suggested that these protons were co-facial and arbitrarily designated as *β*-oriented. Likewise, the ROESY correlations of H-7*′*a with H-6*′*b and of H-3*′*a with H-6*′*a proved these protons were *α*-oriented. However, the relative configuration of Me-13 was left unassigned due to the lack of solid ROESY correlation. In order to determine the absolute configuration, the ECD spectra for two epimers with configurations of 5*R*,7*R*,1*′R*,2*′R*,4*′S* and 5*S*,7*R*,1*′R*,2*′R*,4*′S* were calculated. Finally, the absolute configuration of **3** was defined as 5*R*,7*R*,1*′R*,2*′R*,4*′S* by the calculated ECD spectra (Fig. 4).

Baeckfrutone P (**4**) shared the same molecular formula as **3** by inspection of the HRESIMS *m*/*z* 397.2361 [M + Na]^+^ (calcd for 397.2355). Overall, the NMR data (Tables 2 and 3) of **4** highly resembled those of **3**, revealing that they shared the same planar structure. This was supported by analysis of the ^1^H–^1^H COSY and HMBC correlations (Fig. 2). The ROESY correlations of H-6*′*a with H-7 and H-3*′*a indicated that these protons were co-facial and designated as *α*-oriented, but the ROESY correlations of H-2*′* with H-3*′*a permitted assignment *β*-orientation for H-2*′* (Fig. 3). Moreover, the specific rotation value of **4** (+43.3, in MeOH) revealed it had different absolute configuration of C-7 compared to that of **3** (− 18.5, in MeOH) [9,11], and the ECD spectra for two epimers with different configurations of C-7 were calculated. Finally, the absolute configuration of **4** was assigned as 5*S*,7*S*,1*′R*,2*′S*,4*′R* by the calculated ECD spectrum (Fig. 4).

Baeckfrutone Q (**5**) was proved to have the molecular formula of C_23_H_34_O_3_ as deduced from the HRESIMS *m*/*z* 359.2585 [M + Na]^+^ (calcd for 359.2581). **5** was proved to shared the same planar structure as that of (±)-baeckfrutone I [9] according to analysis of their NMR data, as well as the ^1^H–^1^H COSY and HMBC spectra (Fig. 2). The ROESY correlations of H-7 with H-6*′*a, of H-6*′*b with H-3*′*b suggested that these protons were co-facial and assigned as *α*-oriented (Fig. 3). The calculated ECD spectrum (Fig. 4) confirmed the absolute configuration of **5** as 7*S*,1*′R*,2*′R*,4*′S*.

Baeckfrutone R (**6**) was shown to possess the molecular formula of C_24_H_36_O_3_ according to the HRESIMS *m*/*z* 395.2564 [M + Na]^+^ (calcd for 395.2562), with 14 mass units more than that of **5** indicative of a methyl difference from the latter. The HMBC correlations from the additional methyl at *δ*_H_ 1.83 to C-4/C-5/C-6 indicated that this methyl was attached to C-5. Analysis of its ROESY and comparison of its experimental ECD spectrum with that of **5** revealed that they shared the same relative (Fig. 3) and absolute configurations (Fig. 4).

Baeckfrutone S (**7**) gave the molecular formula of C_28_H_42_O_3_ by the HRESIMS *m*/*z* 449.3036 [M + Na]^+^ (calcd for 449.3032). Comparison of the 1D NMR data of **7** (Table 4) with those of frutescone F [12] revealed that they were structural analogues, differing in the absence of the signals for Me-13. This hypothesis was supported by the HMBC correlations from H-3 to C-1/C-2/C-4/C-5. Additionally, the ROESY correlations of H-1*′* with H-5*′*, of H-5*′* with H-7, and of Me-10 with Me-14*′* indicated it shared the same relative configuration as frutescone F. The absolute configuration of **7** was defined as 7*R*,1*′R*,4*′R*,5*′S*,9*′S* by its calculated ECD spectrum (Fig. 4).

**Table 4:** ^1^H (500 MHz) and ^13^C (125 MHz) NMR data of **7** in CDCl_3_

No.	*δ*_C_	*δ*_H_ (mult., *J* in Hz)
1	112.4	
2	187.3	
3	99.2	5.39 s
4	175.7	
5	41.4	
6	168.5	
7	35.0	2.70 dd (4.6, 3.2)
8	25.9	2.02 m
9	26.6	1.15 d (6.8)
10	19.6	0.66 d (6.9)
11	24.8	1.33 s
12	24.0	1.30 s
1*′*	57.2	1.54 m
2*′*a	23.6	1.58 m
2*′*b		1.35 m
3*′*a	44.7	2.01 m
3*′*b		1.45 brdd (14.2, 9.0)
4*′*	84.2	
5*′*	39.5	1.76 m
6*′*a	24.8	1.76 m
6*′*b		1.72 m
7*′*a	35.5	2.41 m
7*′*b		2.15 m
8*′*	151.2	
9*′*	41.6	2.43 q (9.9)
10*′*a	36.4	1.71 t (10.4)
10*′*b		1.58 dd (10.4, 7.7)
11*′*	34.3	
12*′*	29.8	0.94 s
13*′*	21.7	0.97 s
14*′*	22.9	1.30 s
15*′*	110.8	4.90 2H brs
OMe-4	55.6	3.68

**Scheme 1:**
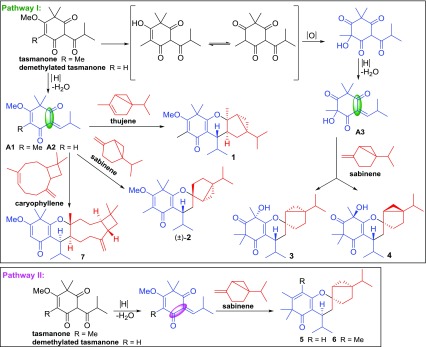
Hypothetical biosynthetic pathways for **1**–**7**

Biogenetically, PPMs **1**–**7** could derive from the same phloroglucinol precursors, tasmanone and demethylated tasmanone. Briefly, the dehydration of the putative precursors would give the intermediates **A1** and **A2**, which could undergo hetero–Diels–Alder (HDA) reactions with thujene, sabinene, and caryophyllene via pathways I and II to yield **1**, **2**, and **5**–**7**. Similarly, the keto-enol interaction, oxidation, and dehydration of tasmanone could afford intermediate **A3**, which would undergo HDA reactions via pathway I with sabinene to yield **3** and **4** (Scheme 1).

These isolates were evaluated for their cytotoxicities against five human cancer cell lines (HL-60, A-549, SMMC-7721, MCF-7, and SW480) and anti-inflammatory effects. However, none of them were cytotoxic at the concentration of 40 μΜ. Compared with the positive control L-*N*^G^-monomethyl arginine (L-NMMA, IC_50_: 54.07 ± 1.05 *μ*M), (+)-**2** and **7** showed more potent inhibitory effects on NO production in LPS stimulated RAW 264.7 macrophages with IC_50_ values of 36.21 ± 1.18 and 20.86 ± 0.60 μM, respectively, whereas others were inactive.

In conclusion, baeckfrutones M–R (**1**–**7**), seven new PPMs were isolated from the twigs and leaves of *B. frutescens*. PPM **1** is a novel meroterpenoid possessing a 6/6/5/3 tetracyclic skeleton, whereas **3** and **4** represent the first hybrids of hydroxytasmanone type phloroglucinol and monoterpene. (+)-**2** and **7** exhibited more potent anti-inflammatory activities. The current study could enrich the phloroglucinol-terpene meroterpenoids from Myrtaceae species.

## Experimental

### General Experimental Procedures

Optical rotations were measured with a Jasco P-1020 polarimeter. UV spectra were obtained using a Shimadzu UV-2401 PC spectrophotometer. IR spectra were obtained with a Bruker FT-IR Tensor 27 spectrometer using KBr pellets. 1D and 2D NMR spectra in CDCl_3_ were recorded on an AVANCE III 500 and 600 MHz spectrometers. HREIMS spectra were recorded on an Agilent 1290 UPLC/6540 Q-TOF mass spectrometer. ECD spectra were recorded on an Applied Photophysics spectropolarimeter. Column chromatography (CC) was performed on silica gel (200–300 mesh, Qingdao Marine Chemical Ltd., Qingdao, China) and RP-18 gel (20–45 µm, Fuji Silysia Chemical Ltd., Tokyo, Japan). Semi-preparative HPLC were performed on an Agilent 1260 instrument with a ZORBAX SB-C18 column (9.4 × 250 mm, 5 μm) or an Agilent 1100 instrument with a CHIRALPAK IC column (10 × 250 mm, 5 μm). TLC (GF_254_, Qingdao Haiyang Chemical Co., Ltd. Qingdao, China or RP-18 F_254_, Merck, Darmstadt, Germany) was used to monitor the fractions. Spots were detected by a UV light (254 nm) and followed by dipping in 10% H_2_SO_4_ in EtOH and heating at 110 °C.

### Plant Material

The stems and leaves of *B. frutescens* were collected in September 2016 from Dongxing city, Guangxi Zhuang Autonomous Region, China, and were authenticated by Prof. Rong Li from Kunming Institute of Botany, Chinese Academy of Sciences. A voucher specimen (No. HY0031) has been deposited at the State Key Laboratory of Phytochemistry and Plant Resources in West China, Kunming Institute of Botany, Chinese Academy of Sciences, Kunming, China.

### Extraction and Isolation

The air-dried and powdered twigs and leaves of *B. frutescens* (10.0 kg) were extracted with MeOH (3 × 50 L) by maceration at room temperature. The solvents were evaporated under reduced pressure to afford a crude extract (1.2 kg). The obtained extract was subjected to a silica gel column chromatography (CC) with a step gradient of petroleum ether (PE)–EtOAc (1:0 → 0:1, v/v) as mobile phase, followed by CHCl_3_–MeOH (1:1 → 0:1, v/v) to afford seven fractions (A–G). Fr. B (25 g) was chromatographed on a silica gel CC with a PE–EtOAc (50:1 → 10:1, v/v) gradient to obtain five (B1–B4) subfractions, respectively. Fr. B3 (420 mg) was further purified by semi-preparative HPLC eluted with MeCN–H_2_O (85:15 → 100:0, v/v) to yield **1** (4.0 mg), **2** (35.4 mg), **5** (8.5 mg), **6** (11.6 mg), and **7** (18.2 mg). Fr. D (16.5 g) was separated by RP-18 CC eluted with MeOH–H_2_O (50:50 → 90:10, v/v) as mobile phase, to yield eight subfractions (D1–D8). Fr. B3 (100 mg) was subsequently purified by semi-preparative HPLC eluted with MeCN–H_2_O (75:25 → 90:10, v/v) to give **3** (11.8 mg) and **4** (4.2 mg). PPM **2** was resolved into (+)-**2** (1.0 mg) and (−)-**2** (1.0 mg) by a semi-preparative chiral column using *n*-hexane–2-propanol (95:5, v/v).

#### Baeckfrutone M (**1**)

Colorless gum; [*α*]$_{\text{D}}^{21}$ +29.3 (*c* 0.10, MeOH); UV (MeOH) *λ*_max_ (log *ε*) 204 (3.67), 250 (3.72), 298 (3.37) nm; ECD (MeOH) *λ*_max_ (Δ*ε*) 251 (+4.23), 294 (+3.57), 333 (− 0.58) nm; ^1^H (500 MHz, CDCl_3_) and ^13^C (125 MHz, CDCl_3_) NMR data, see Table 1; (+)-HRESIMS *m*/*z* 395.2567 [M + Na]^+^ (calcd for C_24_H_36_O_3_Na, 395.2562).

#### (±)-Baeckfrutone N (**2**)

Colorless gum; [*α*]$_{\text{D}}^{20}$ +60.8 (*c* 0.10, MeOH) for (+)-**2**, [*α*]$_{\text{D}}^{20}$ −58.2 (*c* 0.10, MeOH) for (−)-**2**; UV (MeOH) *λ*_max_ (log *ε*) 203 (3.69), 249 (3.71), 296 (3.35) nm; ECD (MeOH) *λ*_max_ (Δε) 250 (+13.06), 291 (+8.79), 329 (−0.86) nm for (+)-**2**, ECD (MeOH) *λ*_max_ (Δε) 251 (−13.75), 292 (−9.24), 330 (+0.90) nm for (−)-**2**; ^1^H (500 MHz, CDCl_3_) and ^13^C (125 MHz, CDCl_3_) NMR data, see Tables 2 and 3; (+)-HRESIMS *m*/*z* 395.2568 [M + Na]^+^ (calcd for C_24_H_36_O_3_Na, 395.2562).

#### Baeckfrutone O (**3**)

Colorless gum; [*α*]$_{\text{D}}^{20}$ −18.5 (*c* 0.12, MeOH); UV (MeOH) *λ*_max_ (log *ε*) 265 (3.82) nm; ECD (MeOH) *λ*_max_ (Δ*ε*) 208 (−4.44), 268 (+4.41), 308 (−1.37); ^1^H (600 MHz, CDCl_3_) and ^13^C (150 MHz, CDCl_3_) NMR data, see Tables 2 and 3; (+)-HRESIMS *m*/*z* 397.2358 [M + Na]^+^ (calcd for C_23_H_34_O_4_Na, 397.2355).

#### Baeckfrutone P (**4**)

Colorless gum; [*α*]$_{\text{D}}^{20}$ +43.3 (*c* 0.15, MeOH); UV (MeOH) *λ*_max_ (log *ε*) 265 (3.79) nm; ECD (MeOH) *λ*_max_ (Δ*ε*) 215 (+1.68), 267 (−6.83), 317 (+0.82) nm; ^1^H (500 MHz, CDCl_3_) and ^13^C (125 MHz, CDCl_3_) NMR data, see Tables 2 and 3; (+)-HRESIMS *m*/*z* 397.2361 [M + Na]^+^ (calcd for C_23_H_34_O_4_Na, 397.2355).

#### Baeckfrutone Q (**5**)

Colorless gum; [*α*]$_{\text{D}}^{21}$ +21.1 (*c* 0.10, MeOH); UV (MeOH) *λ*_max_ (log *ε*) 223 (4.04), 345 (3.47) nm; ECD (MeOH) *λ*_max_ (Δ*ε*) 231 (− 10.19), 303 (− 7.55), 346 (+6.23) nm; ^1^H (500 MHz, CDCl_3_) and ^13^C (150 MHz, CDCl_3_) NMR data, see Tables 2 and 3; (+)-HRESIMS *m*/*z* 359.2585 [M + Na]^+^ (calcd for C_23_H_34_O_3_Na, 359.2581).

#### Baeckfrutone R (**6**)

Colorless gum; [*α*]$_{\text{D}}^{20}$ +20.6 (*c* 0.10, MeOH); UV (MeOH) *λ*_max_ (log *ε*) 221 (4.38), 338 (3.81) nm; ECD (MeOH) *λ*_max_ (Δε) 229 (−7.94), 304 (−7.26), 348 (+5.45) nm; ^1^H (500 MHz, CDCl_3_) and ^13^C (125 MHz, CDCl_3_) NMR data, see Tables 2 and 3; (+)-HRESIMS *m*/*z* 395.2564 [M + Na]^+^ (calcd for C_24_H_36_O_3_Na, 395.2562).

#### Baeckfrutone S (**7**)

Colorless gum; [*α*]$_{\text{D}}^{20}$ −42.5 (*c* 0.10, MeOH); UV (MeOH) *λ*_max_ (log *ε*) 203 (4.10), 245 (4.06), 298 (3.73) nm; ECD (MeOH) *λ*_max_ (Δ*ε*) 216 (+16.48), 254 (−3.04), 302 (−3.43) nm; ^1^H (500 MHz, CDCl_3_) and ^13^C (125 MHz, CDCl_3_) NMR data, see Table 3; (+)-HRESIMS *m*/*z* 449.3036 [M + Na]^+^ (calcd for C_28_H_42_O_3_Na, 449.3032).

### ECD Calculation

The ECD calculations of **1**–**7** were performed using Gaussian 09 [13]. Briefly, the 3D structures of these compounds were first established according to the observed ROESY correlations. Conformational analysis was performed using CONFLEX software at the MMFF94s level with an energy cutoff of 1.0 kcal/mol. The conformers were further optimized by using the Density Functional Theory (DFT) at the B3LYP/6-31 + G(d) level in gas phase. The optimized conformations were finally subjected to ECD calculations using Time Dependent DFT (TDDFT) at the B3LYP/6-311 ++G(2d,p) level in MeOH. The calculated ECD spectra were produced by SpecDis software [14], which were subsequently compared with their experimental spectra.

### Cytotoxic Assay

The cytotoxic assay with taxol as a positive control was conducted by MTT method in 96-well plates as our previously described [10].

### NO Production Assay

The NO production assay with L-*N*^G^-monomethyl arginine (L-NMMA) as a positive control was performed as our described previously [15].

## Electronic supplementary material

Below is the link to the electronic supplementary material.

Supplementary material 1 (PDF 10447 kb)
