# Correction to: Structurally Diverse Polymethylated Phloroglucinol Meroterpenoids from *Baeckea frutescens*

**DOI:** 10.1007/s13659-018-0196-4

**Published:** 2018-12-24

**Authors:** Yin-E Zhi, Xu-Jie Qin, Hui Liu, Yuan Zeng, Wei Ni, Li He, Zu-Ding Wang, Hai-Yang Liu

**Affiliations:** 1 State Key Laboratory of Phytochemistry and Plant Resources in West China, Kunming Institute of Botany, Chinese Academy of Sciences, and Yunnan Key Laboratory of Medicinal Chemistry, Kunming, 650201 China; 2 Department of Dermatology, The First Affiliated Hospital of Kunming Medical University, Kunming, 650032 China; 3 Kunming Botanee Bio-Technique Co. Ltd, Kunming, 650106 China

## Correction to: Natural Products and Bioprospecting (2018) 8:431–439 10.1007/s13659-018-0189-3

In the original publication, two errors have occurred. The corrected texts are provided below:

1. The author, Xu-Jie Qi, should read as Xu-Jie Qin among the author group.
2. The reference 9 should be cited as X.J. Qin, Y.E. Zhi, H. Yan, Y. Zhang, H. Liu, Q. Yu, S. Wang, Q. Zhao, L. He, X. Ma, L.K. An, H.Y. Liu, Tetrahedron **74**, 6658–6666 (2018).

