# Supplementary material for: Structurally Diverse Polymethylated Phloroglucinol Meroterpenoids from Baeckea frutescens
Source: Nat Prod Bioprospect. 2018 Oct 29;8(6):431–9. doi: 10.1007/s13659-018-0189-3 (PMC6224813; doi:10.1007/s13659-018-0189-3)
Supplement: Supplementary file 1 — Supplementary material 1 (PDF 10447 kb) [file 13659_2018_189_MOESM1_ESM.pdf]

Structurally diverse polymethylated phloroglucinol meroterpenoids from *Baeckea frutescens*

Yin-E Zhi , Xu-Jie Qi, Hui Liu, Yuan Zeng, Wei Ni, Li He, Zu-Ding Wang, Hai-Yang Liu*

State Key Laboratory of Phytochemistry and Plant Resources in West China, Kunming Institute of Botany, Chinese Academy of Sciences, and Yunnan Key Laboratory of Medicinal Chemistry, Kunming 650201, China

Department of Dermatology, The First Affiliated Hospital of Kunming Medical University, Kunming 650032, China

Kunming Botanee Bio-Technique Co. Ltd., Kunming 650106, China

List of Content

Figure S1−S7 NMR and HRESIMS data of **1**……………………………………………………………..…..1–4

Figure S8−S14 NMR and HRESIMS data of **2**…………………………………………………..…………4–7

Figure S15−S21 NMR and HRESIMS data of **3**………………………………………………..……..……8–11

Figure S22−S28 NMR and HRESIMS data of **4**……………………………………….………………..……11–14

Figure S29−S35 NMR and HRESIMS data of **5**……………………………………….………………..……15–18

Figure S26−S42 NMR and HRESIMS data of **6**……………………………………….………………..……18–21

Figure S43−S49 NMR and HRESIMS data of **7**……………………………………….………………..……22–25

Computational data of 1–5 and 7……………………………………………………………………………26–28

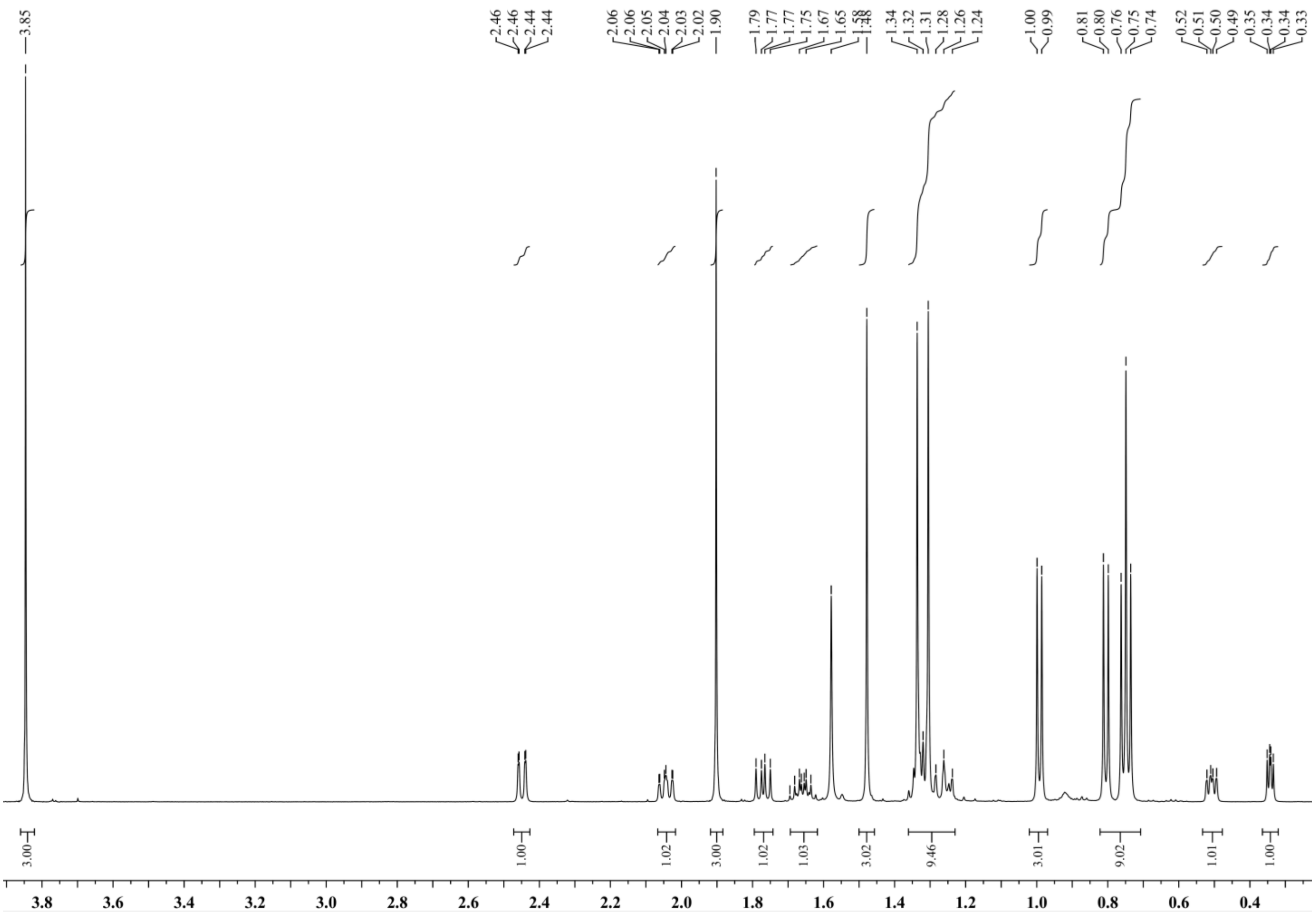

Figure S1. ^{1}H NMR spectroscopic data of **1**

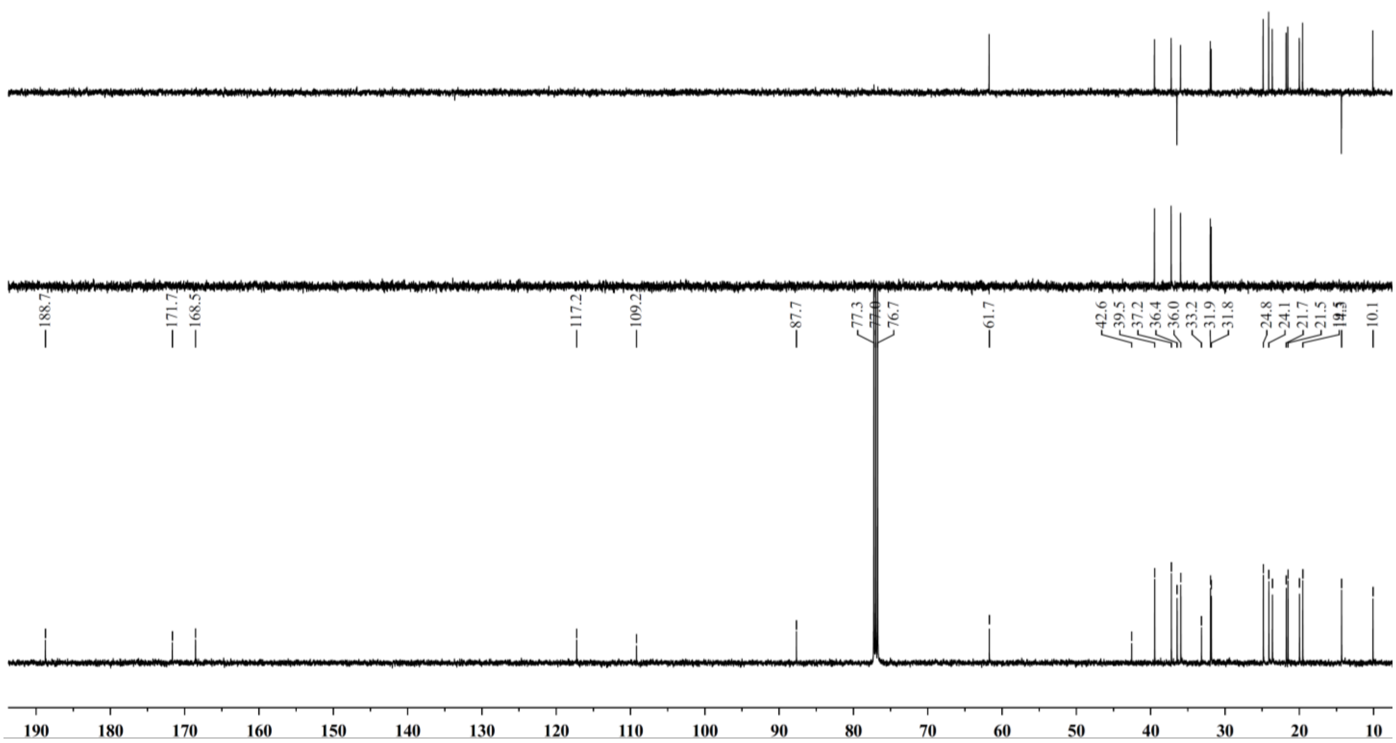

Figure S2. ^{13}C NMR spectroscopic data of **1**

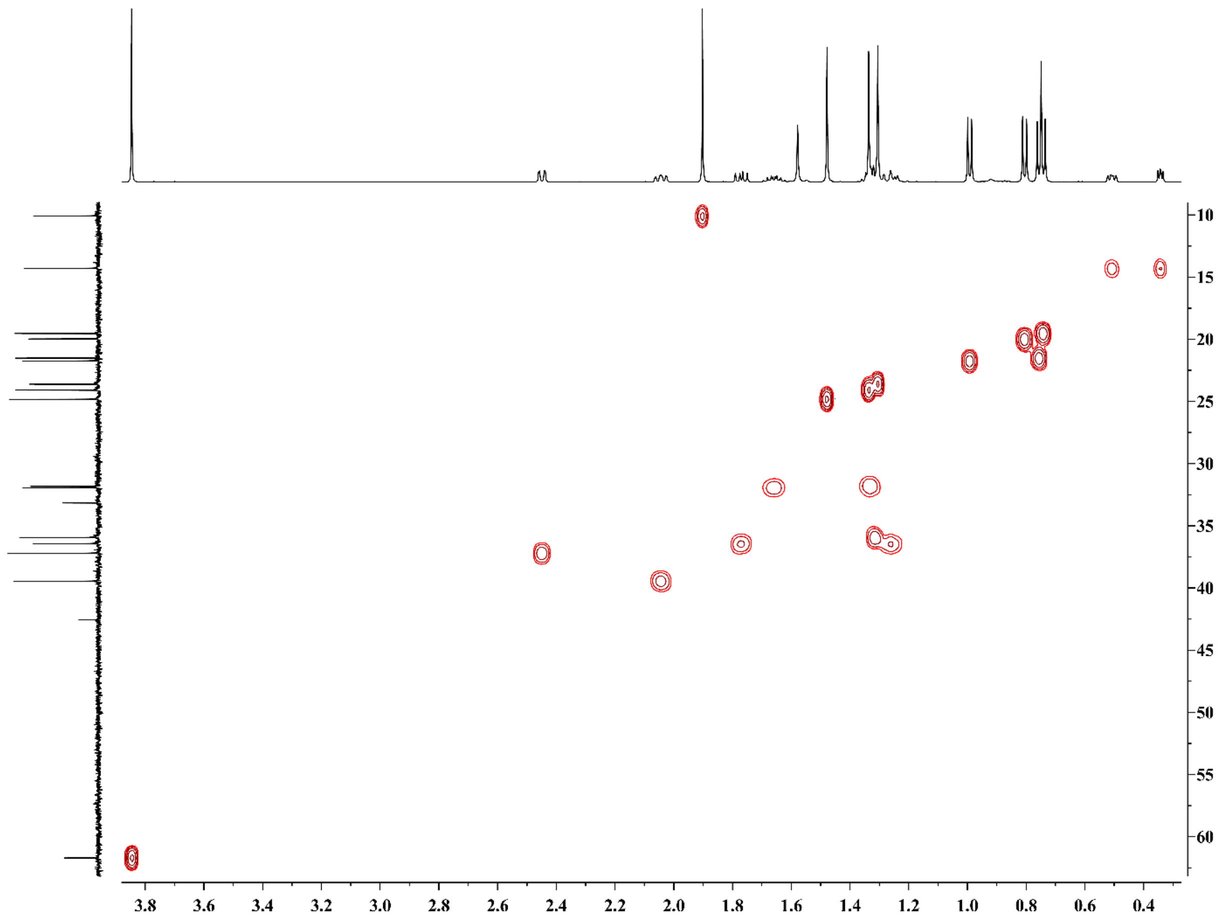

Figure S3. HSQC spectroscopic data of **1**

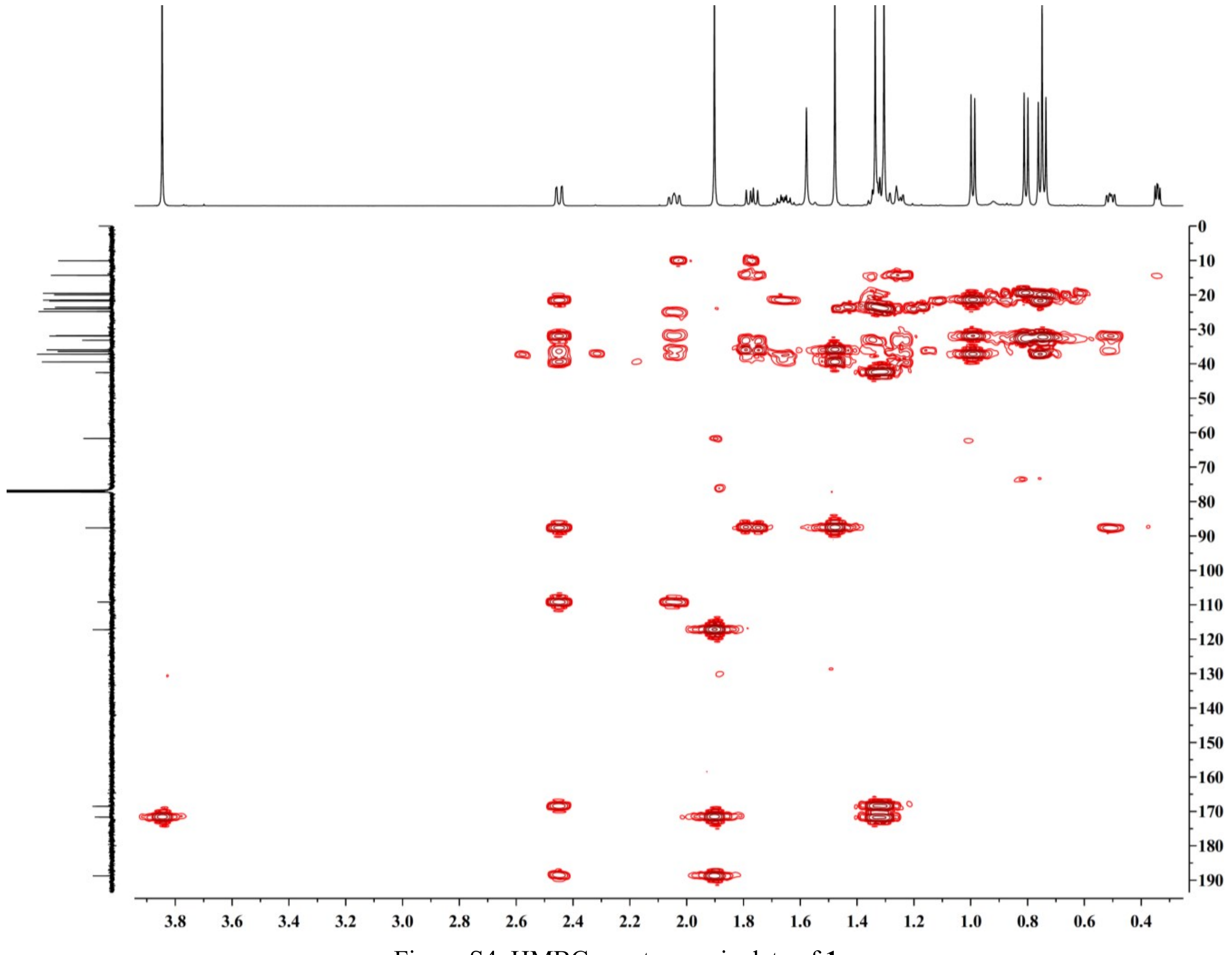

Figure S4. HMBC spectroscopic data of **1**

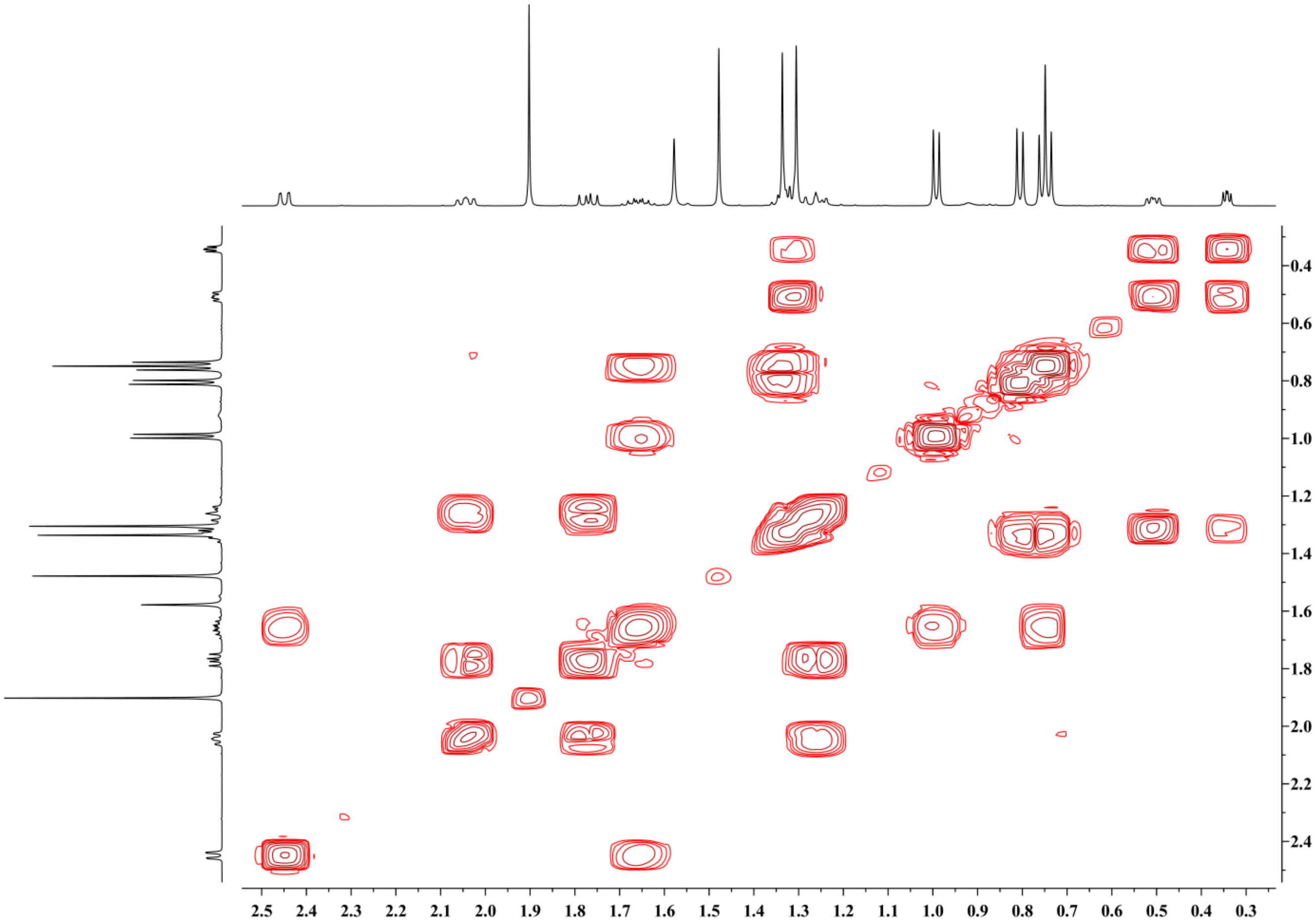

Figure S5. ^{1}H–^{1}H COSY spectroscopic data of **1**

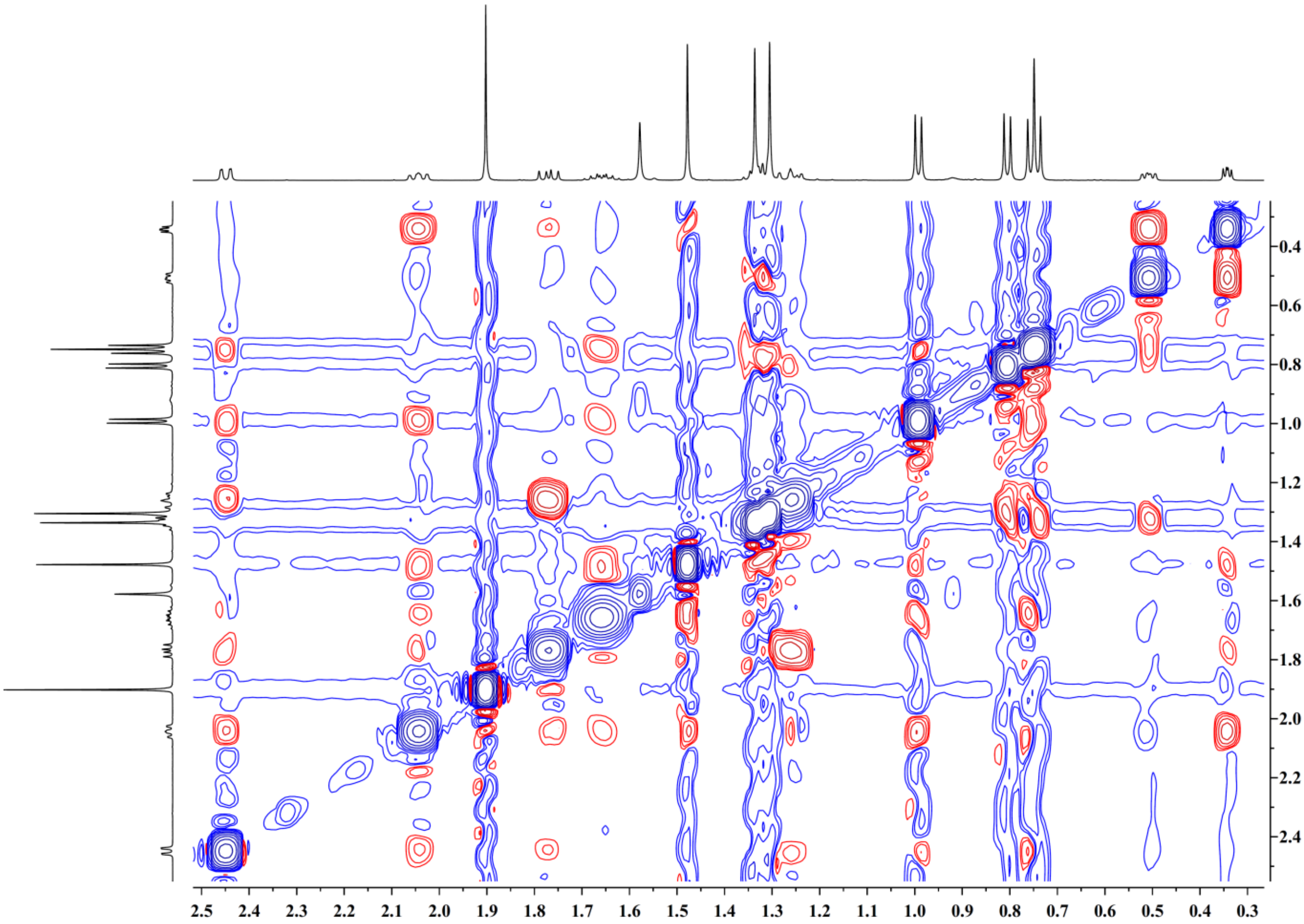

Figure S6. ROESY spectroscopic data of **1**

User Spectra

Fragmentor Voltage	Collision Energy	Ionization Mode
250	0	ESI

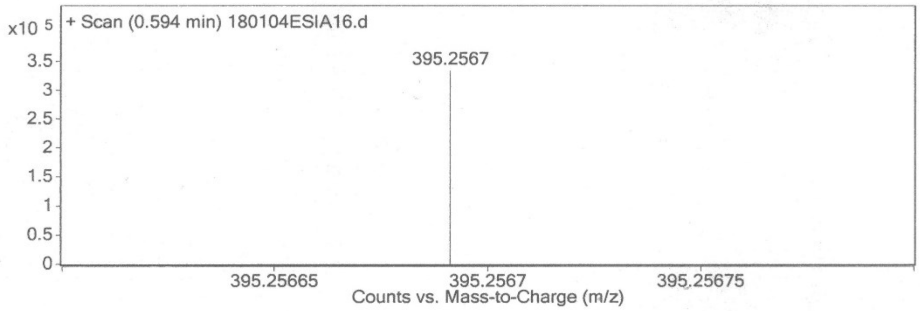

Peak List

m/z	z	Abund	Formula	Ion
121.0509	1	13723.79		
237.1485	1	51511.8		
373.2739	1	35292.46		
395.2567	1	334896.97	C24 H36 Na O3	M+
396.2594	1	71333.4	C24 H36 Na O3	M+
411.2294	1	14677.27		
767.5235	1	348966.88		
768.5264	1	174128.25		
769.5287	1	35736.1		
922.0098	1	47282.09		

Formula Calculator Element Limits

Element	Min	Max
C	0	200
H	0	400
O	0	10
Na	1	1

Formula Calculator Results

Formula	CalculatedMass	Mz	Diff.(mDa)	Diff. (ppm)	DBE
C24 H36 Na O3	395.2562	395.2567	-0.5	1.2	6.5

Figure S7. HRESIMS spectroscopic data of **1**

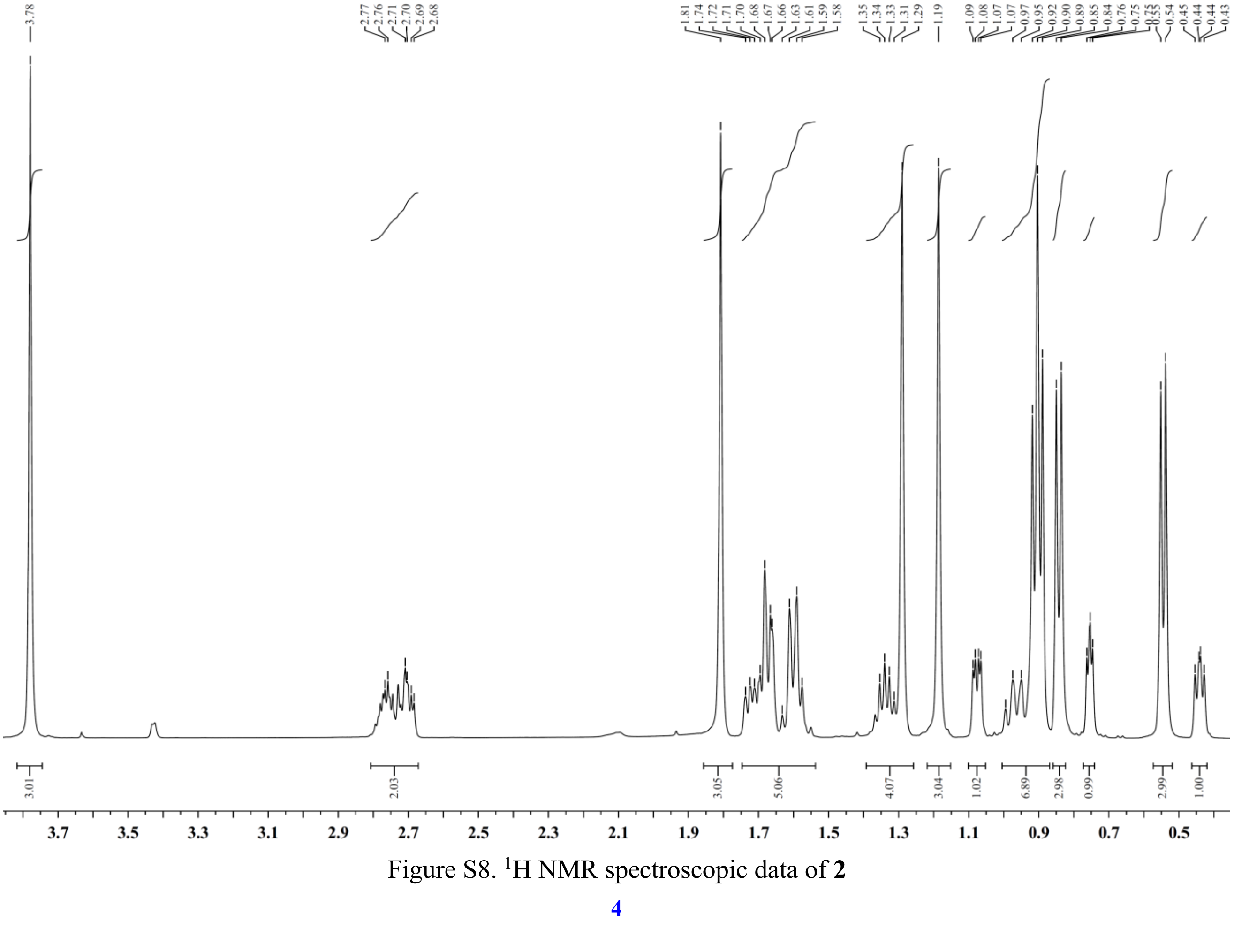

Figure S8. ^{1}H NMR spectroscopic data of **2**

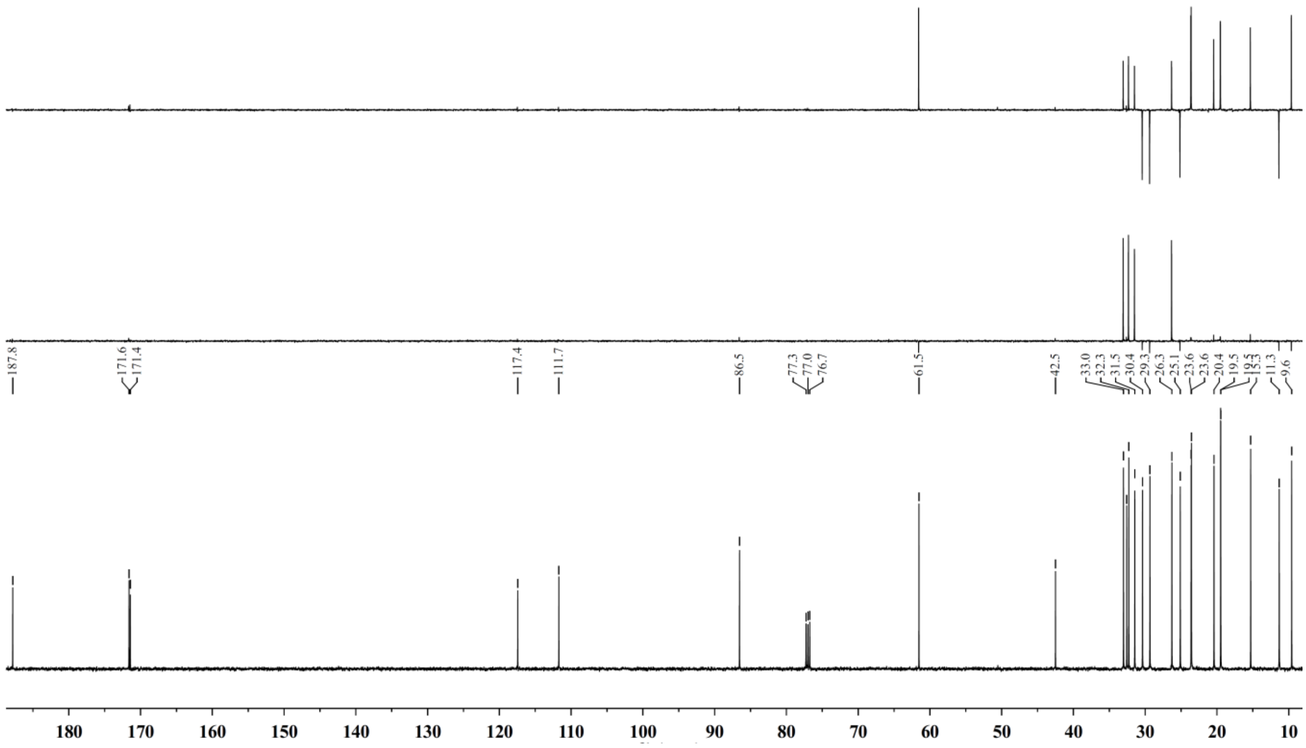

Figure S9. ^{13}C NMR spectroscopic data of **2**

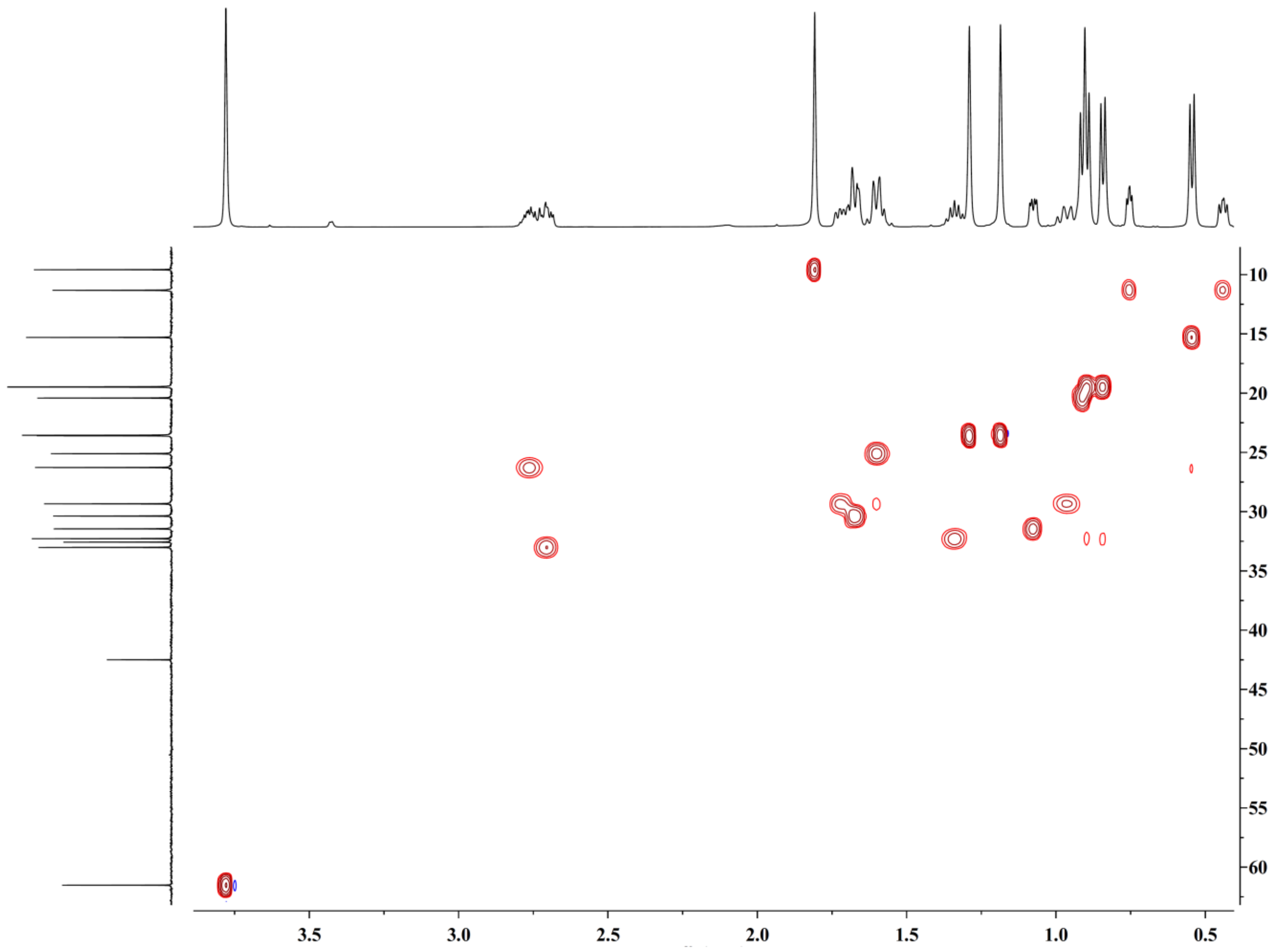

Figure S10. HSQC spectroscopic data of **2**

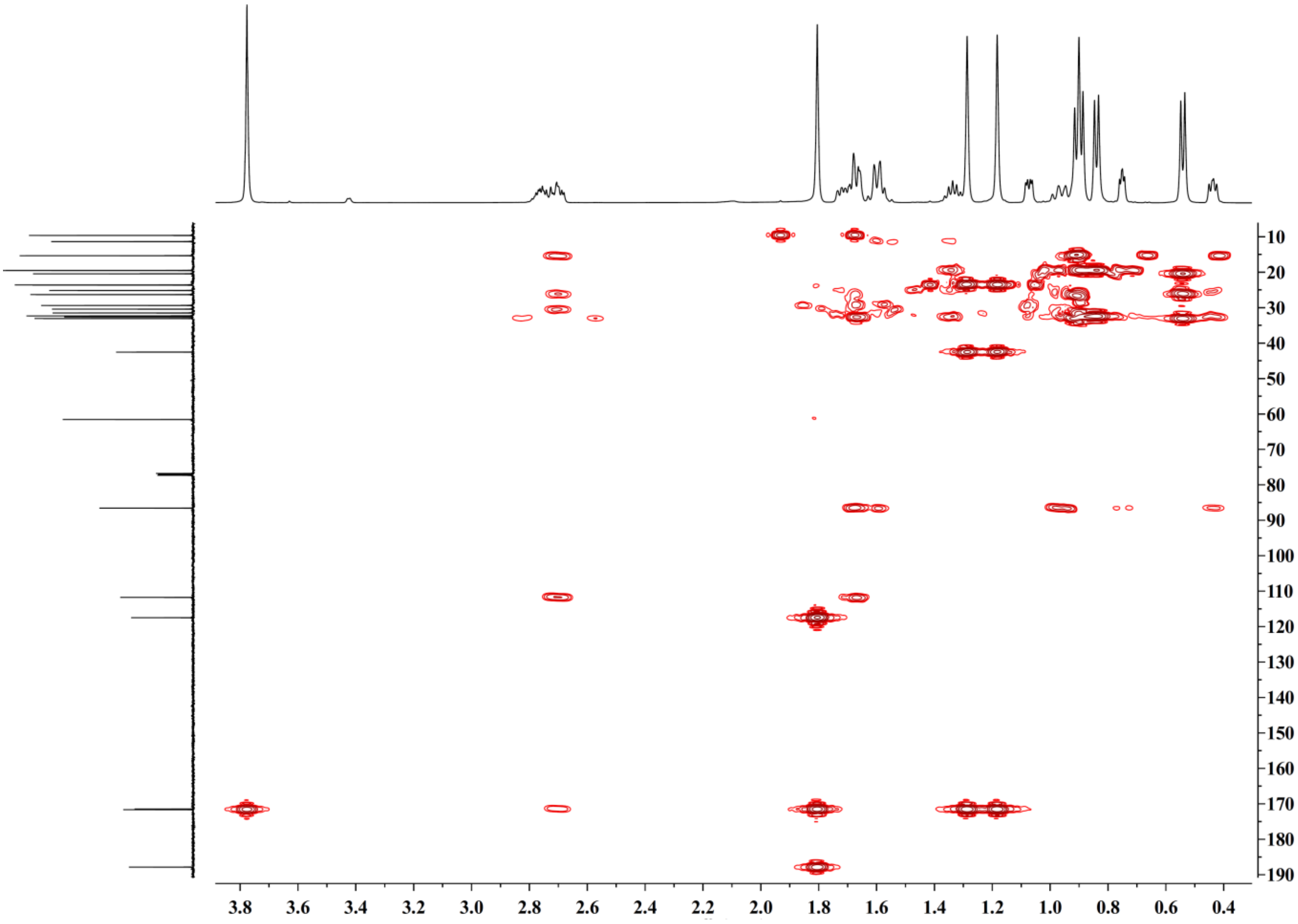

Figure S11. HMBC spectroscopic data of **2**

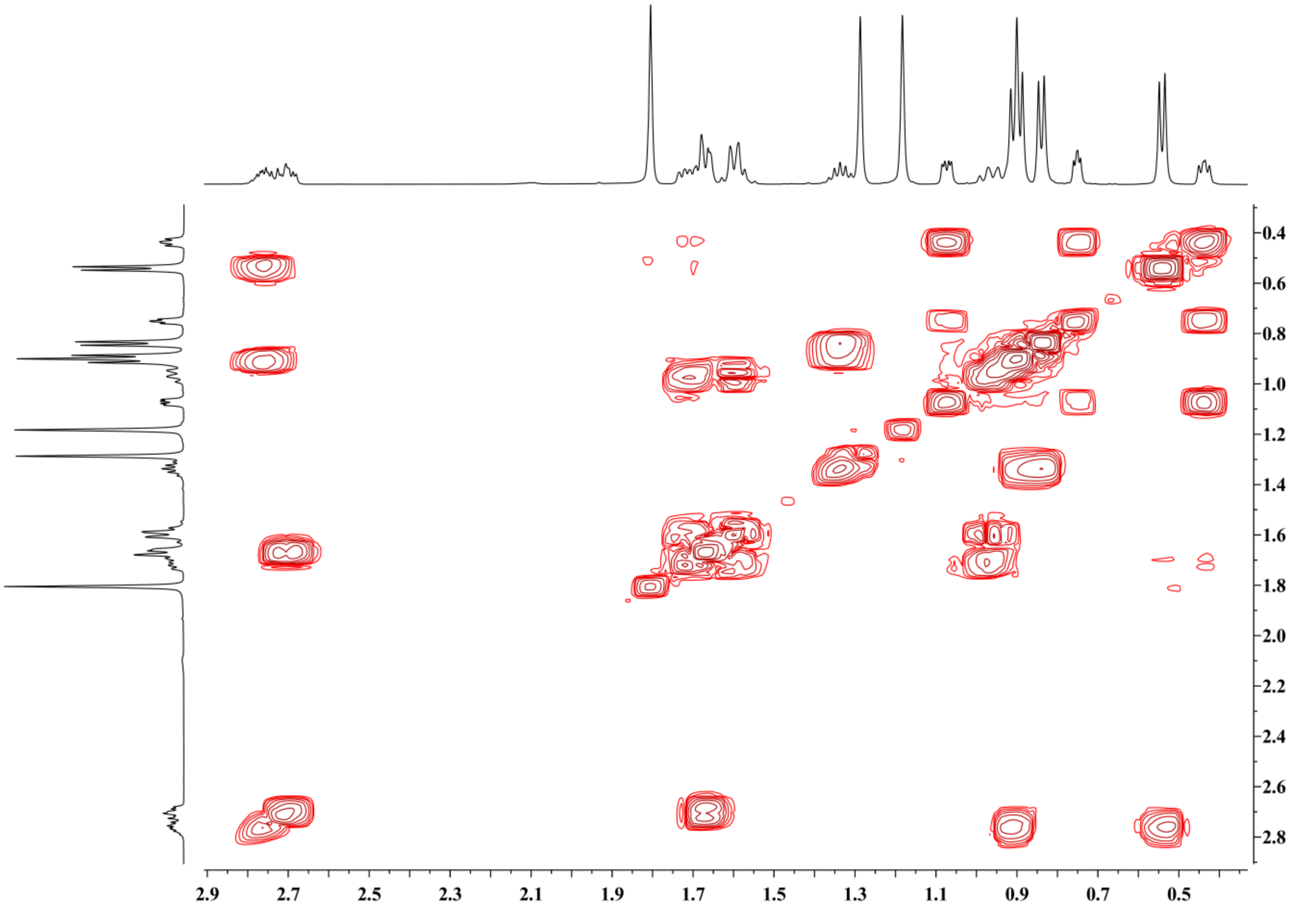

Figure S12. ^{1}H–^{1}H COSY spectroscopic data of **2**

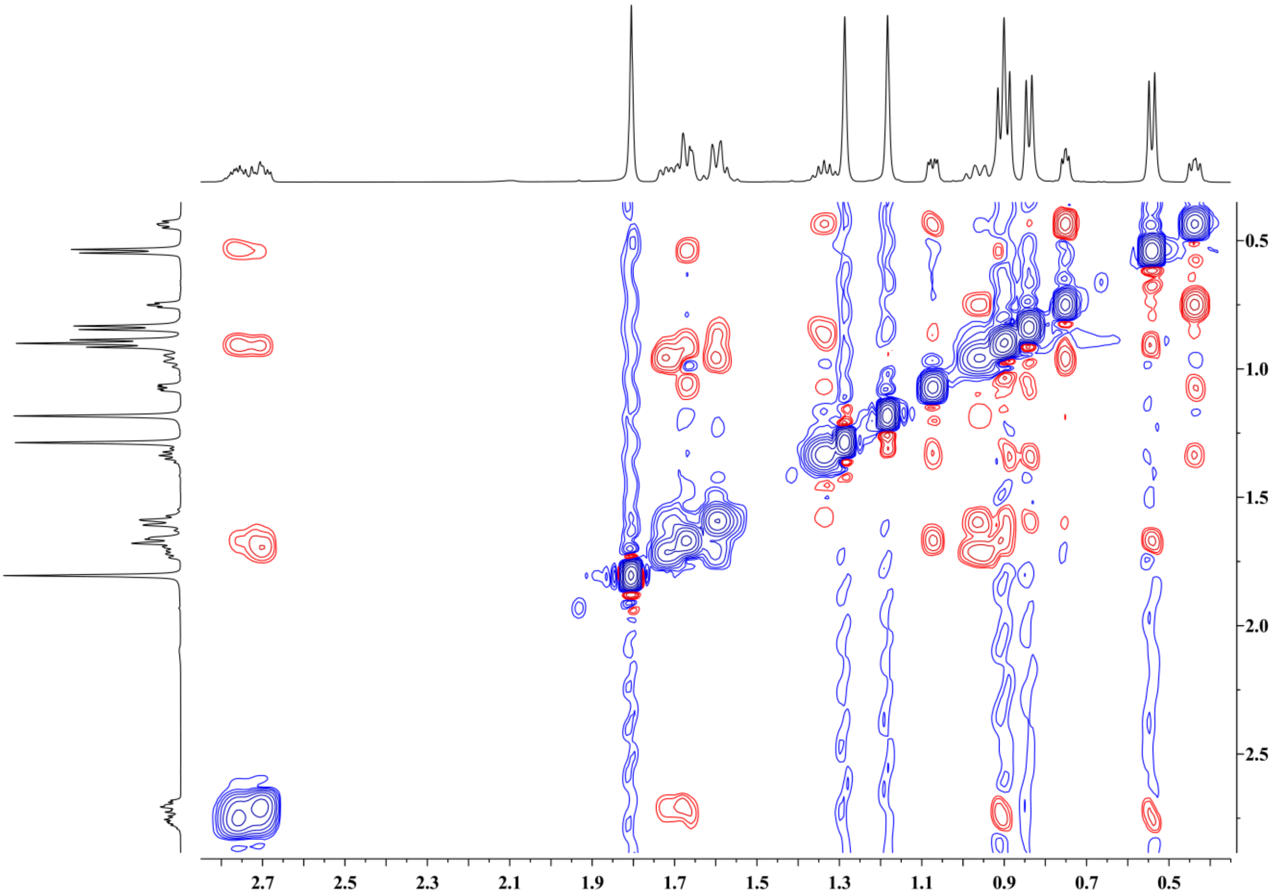

Figure S13. ROESY spectroscopic data of **2**

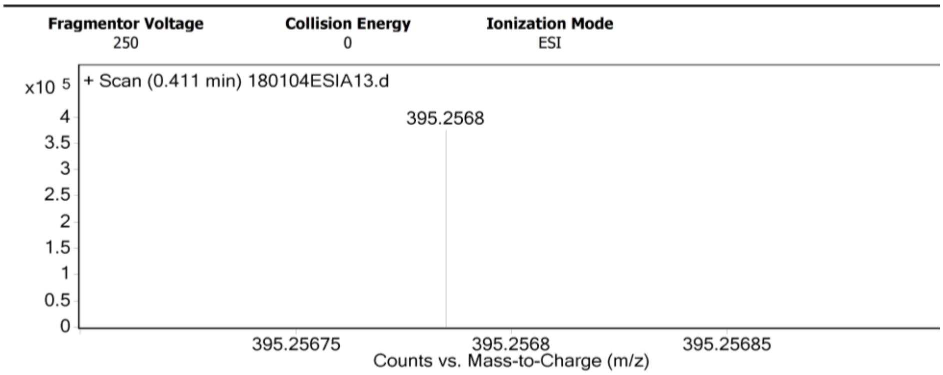

Peak List

m/z	z	Abund	Formula	Ion
121.0509	1	14883.04		
237.1483	1	47656.75		
373.2738	1	25738.46		
395.2568	1	374733.5	C24 H36 Na O3	M+
396.2595	1	80820.34	C24 H36 Na O3	M+
411.2295	1	20309.08		
767.5241	1	429814.53		
768.5272	1	215509.23		
769.5292	1	44847.32		
922.0098	1	48641.22		

Formula Calculator Element Limits

Element	Min	Max
C	0	200
H	0	400
O	0	10
Na	1	1

Formula Calculator Results

Formula	CalculatedMass	Mz	Diff.(mDa)	Diff. (ppm)	DBE
C24 H36 Na O3	395.2562	395.2568	-0.6	1.5	6.5

Figure S14. HRESIMS spectroscopic data of **2**

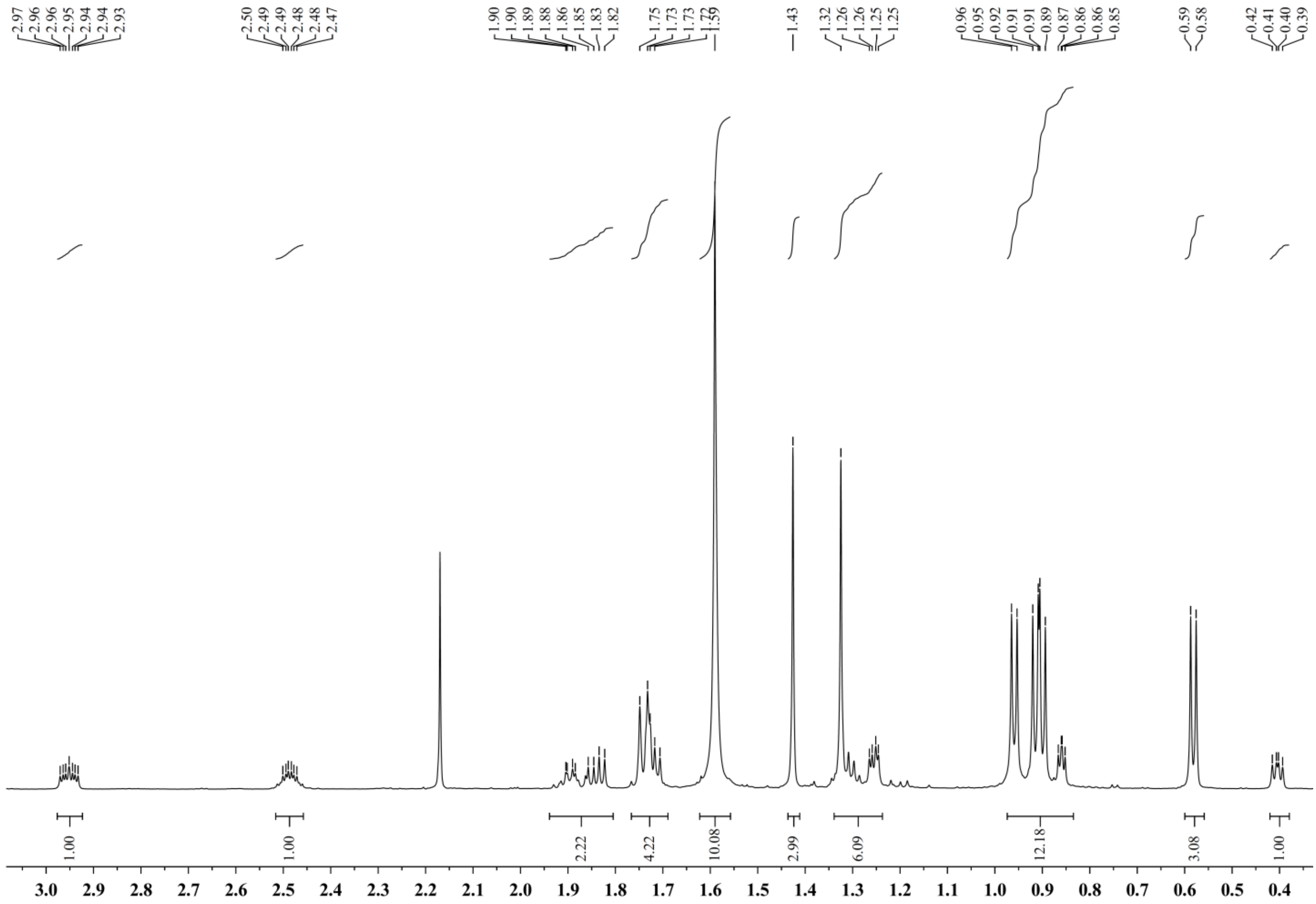

Figure S15. ^{1}H NMR spectroscopic data of **3**

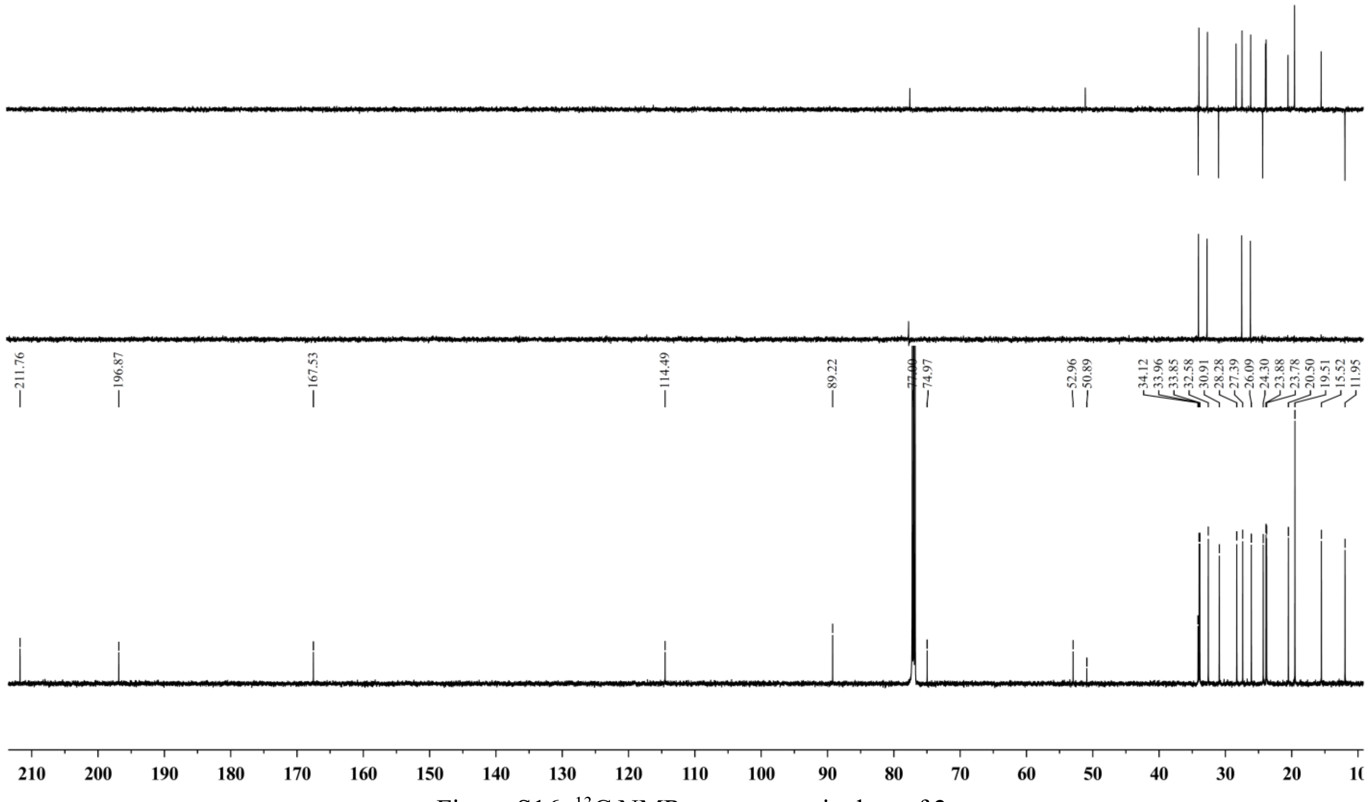

Figure S16. ^{13}C NMR spectroscopic data of **3**

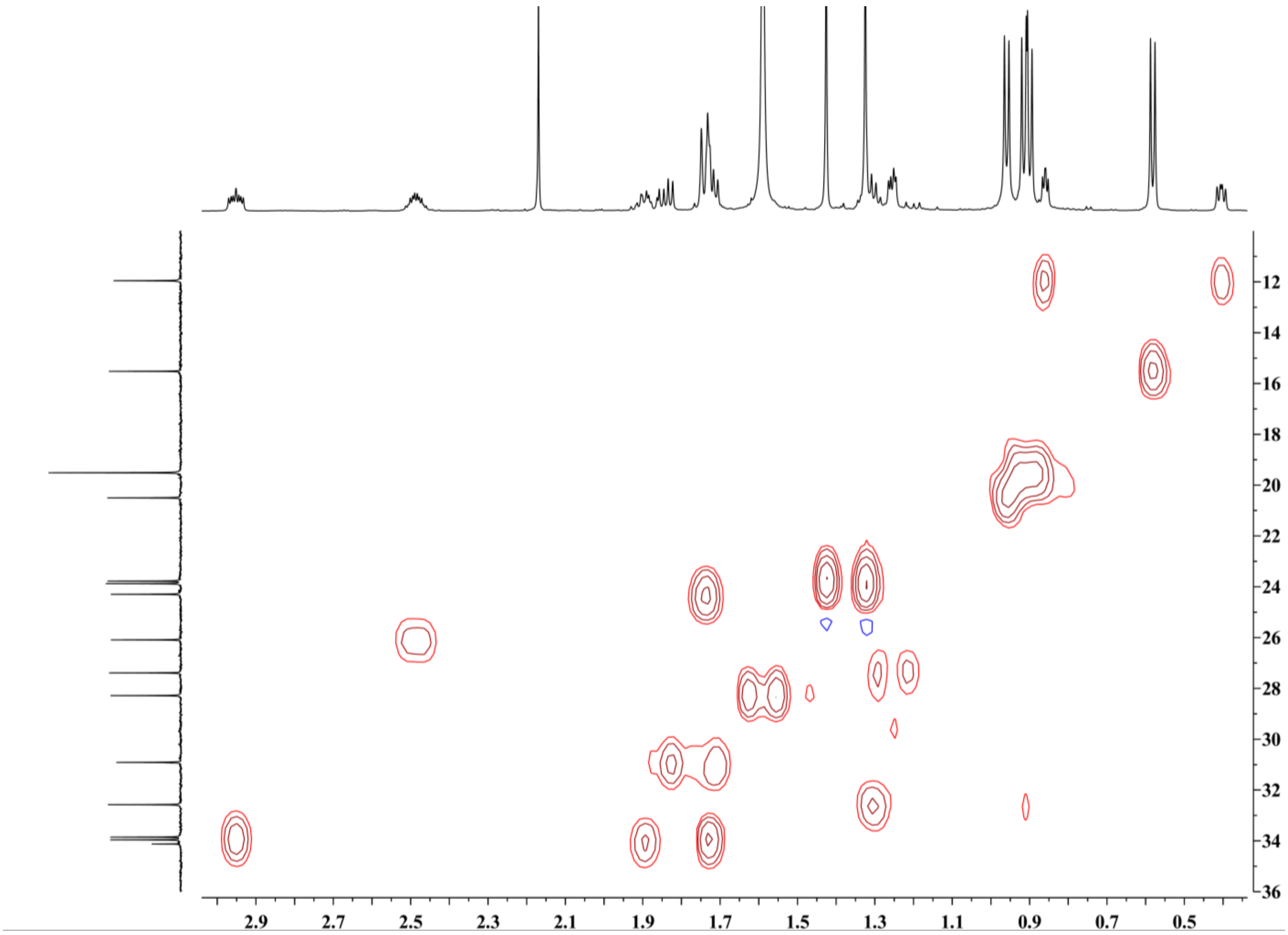

Figure S17. HSQC spectroscopic data of **3**

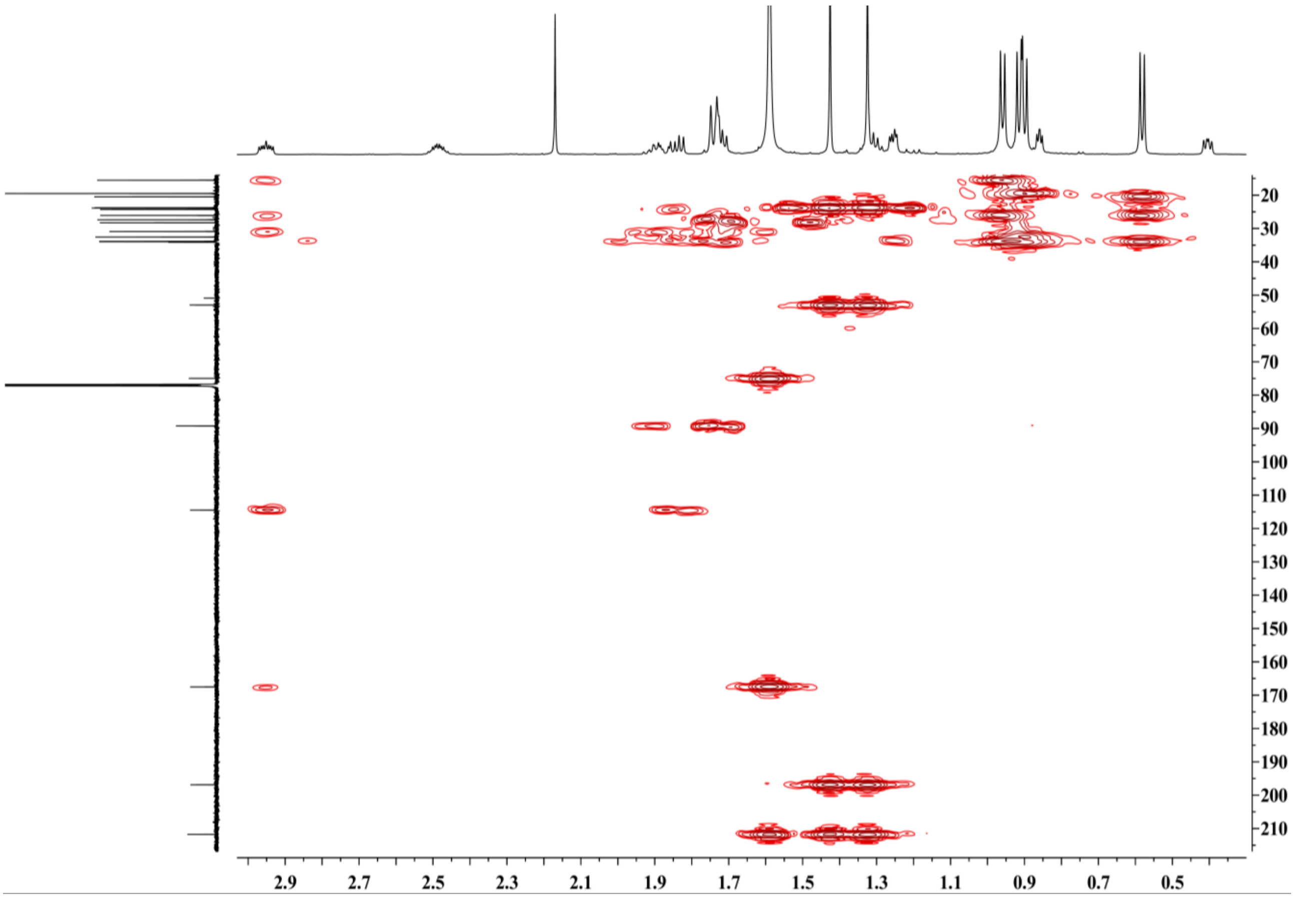

Figure S18. HMBC spectroscopic data of **3**

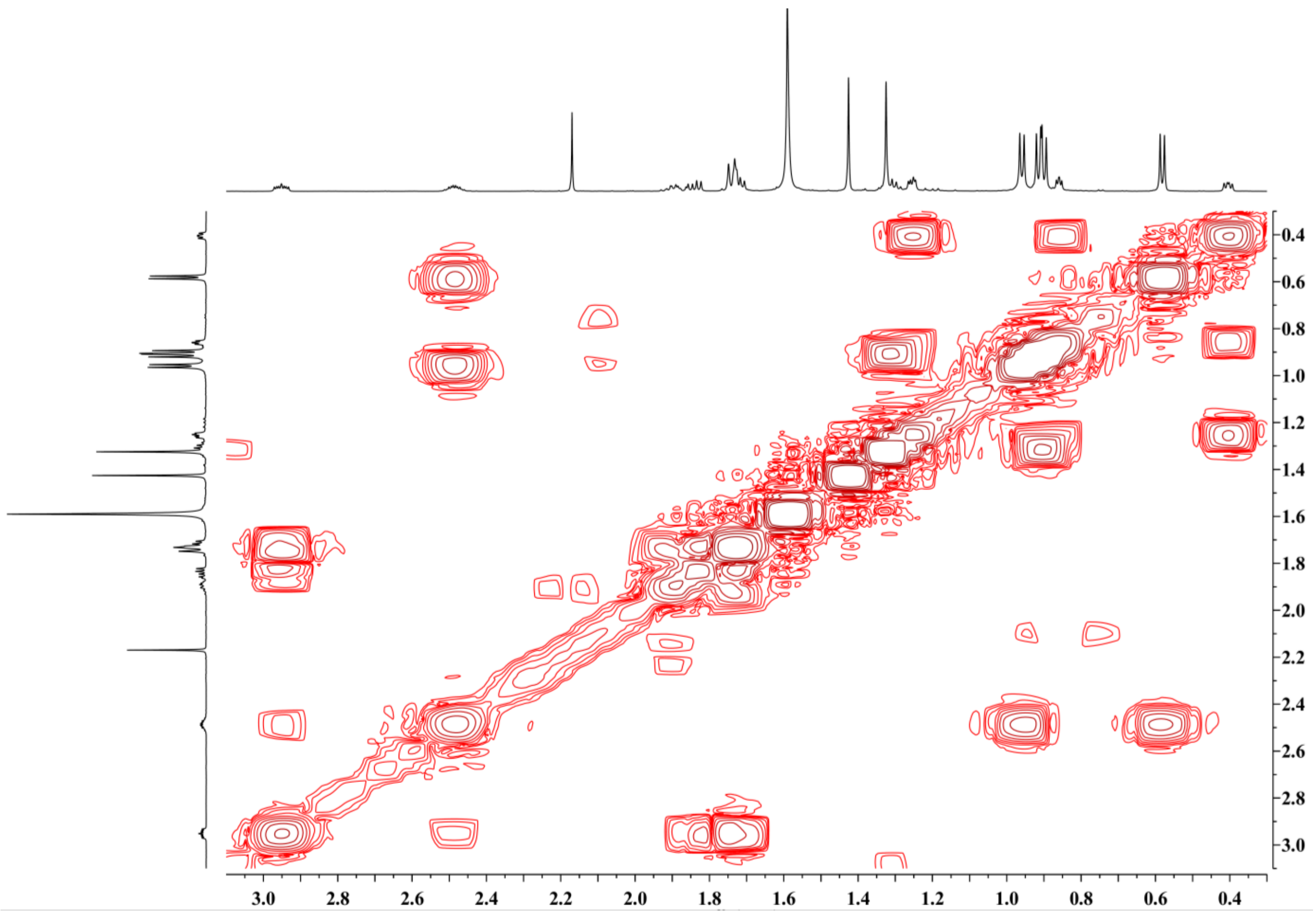

Figure S19. ^{1}H–^{1}H COSY spectroscopic data of **3**

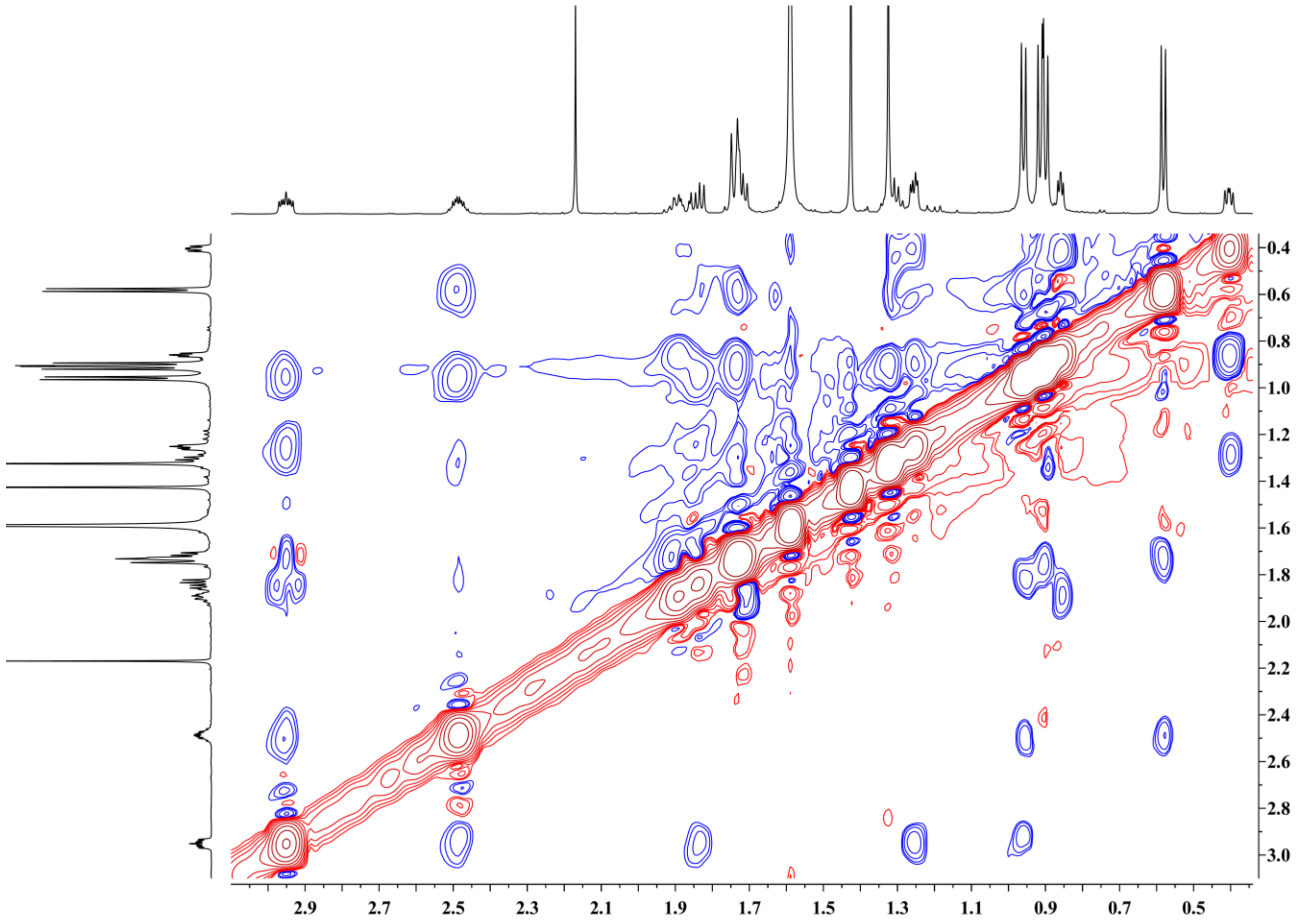

Figure S20. ROESY spectroscopic data of **3**

User Spectra

Fragmentor Voltage	Collision Energy	Ionization Mode
250	0	ESI

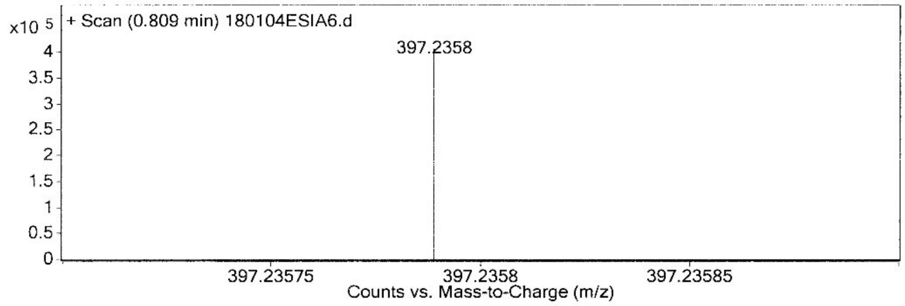

Peak List

m/z	z	Abund	Formula	Ion
261.1092	1	99375.87		
397.2358	1	406713.22	C23 H34 Na O4	M+
398.2385	1	82986.1	C23 H34 Na O4	M+
411.251	1	179042.22		
412.2538	1	37473.98		
413.2658	1	192606.05		
414.2685	1	37828.1		
771.4817	1	152102.91		
772.4843	1	66278.34		
922.0098	1	46548.69		

Formula Calculator Element Limits

Element	Min	Max
C	0	200
H	0	400
O	0	10
Na	1	1

Formula Calculator Results

Formula	CalculatedMass	Mz	Diff.(mDa)	Diff. (ppm)	DBE
C23 H34 Na O4	397.2355	397.2358	-0.3	0.8	6.5

Figure S21. HRESIMS spectroscopic data of **3**

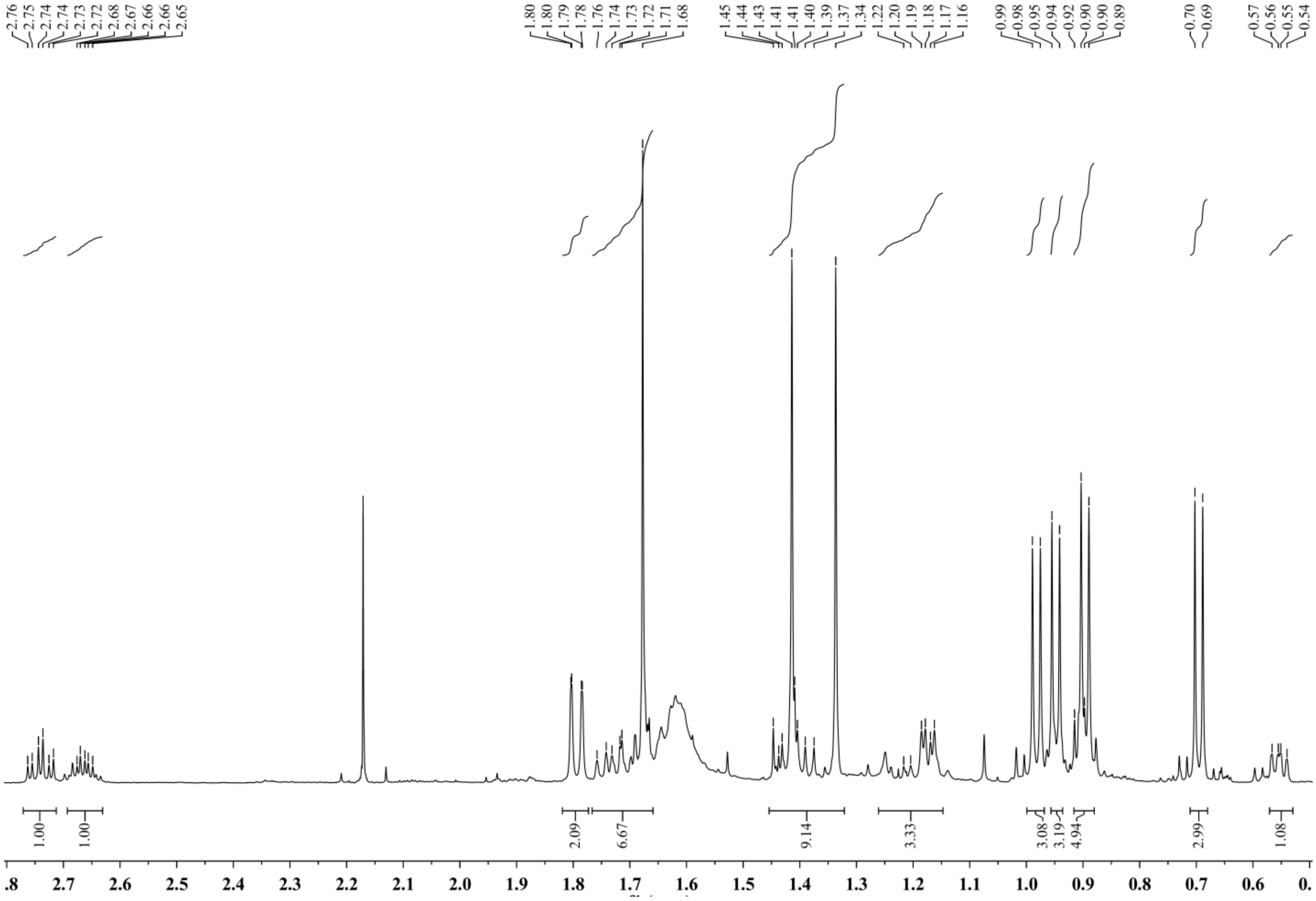

Figure S22. ^{1}H NMR spectroscopic data of **4**

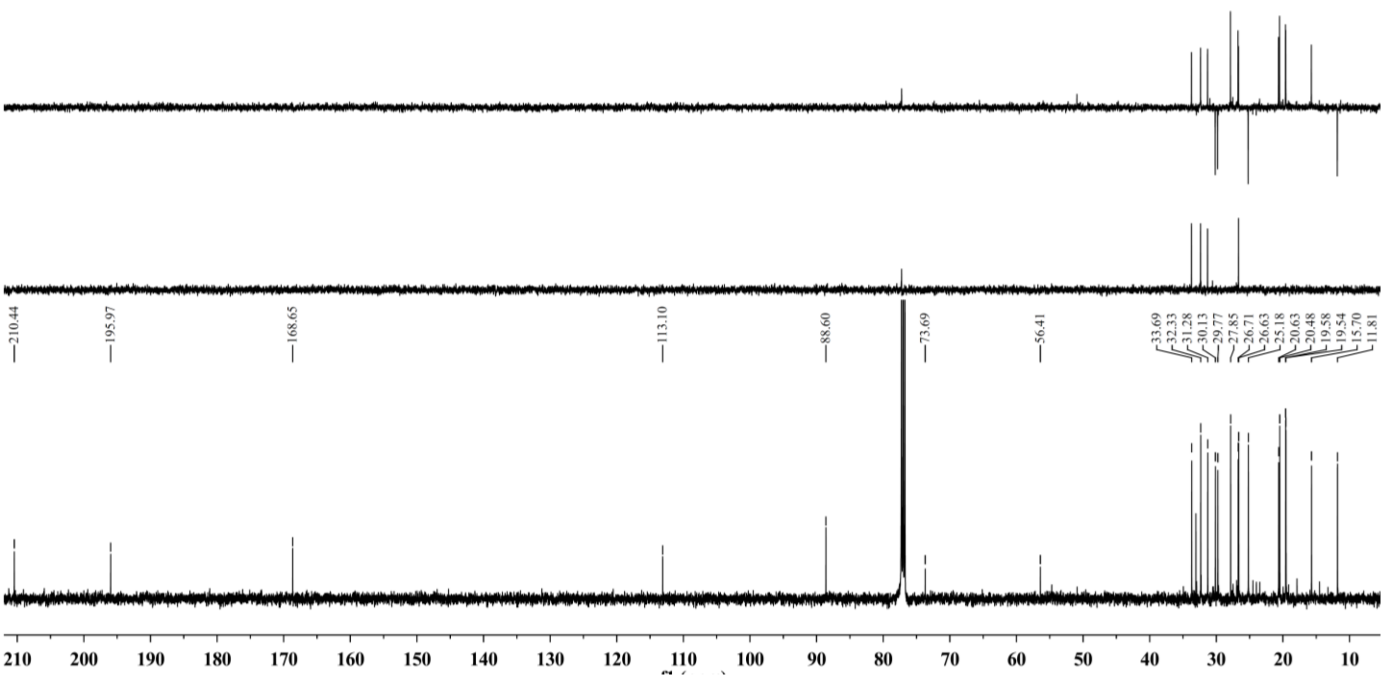

Figure S23. ^{13}C NMR spectroscopic data of **4**

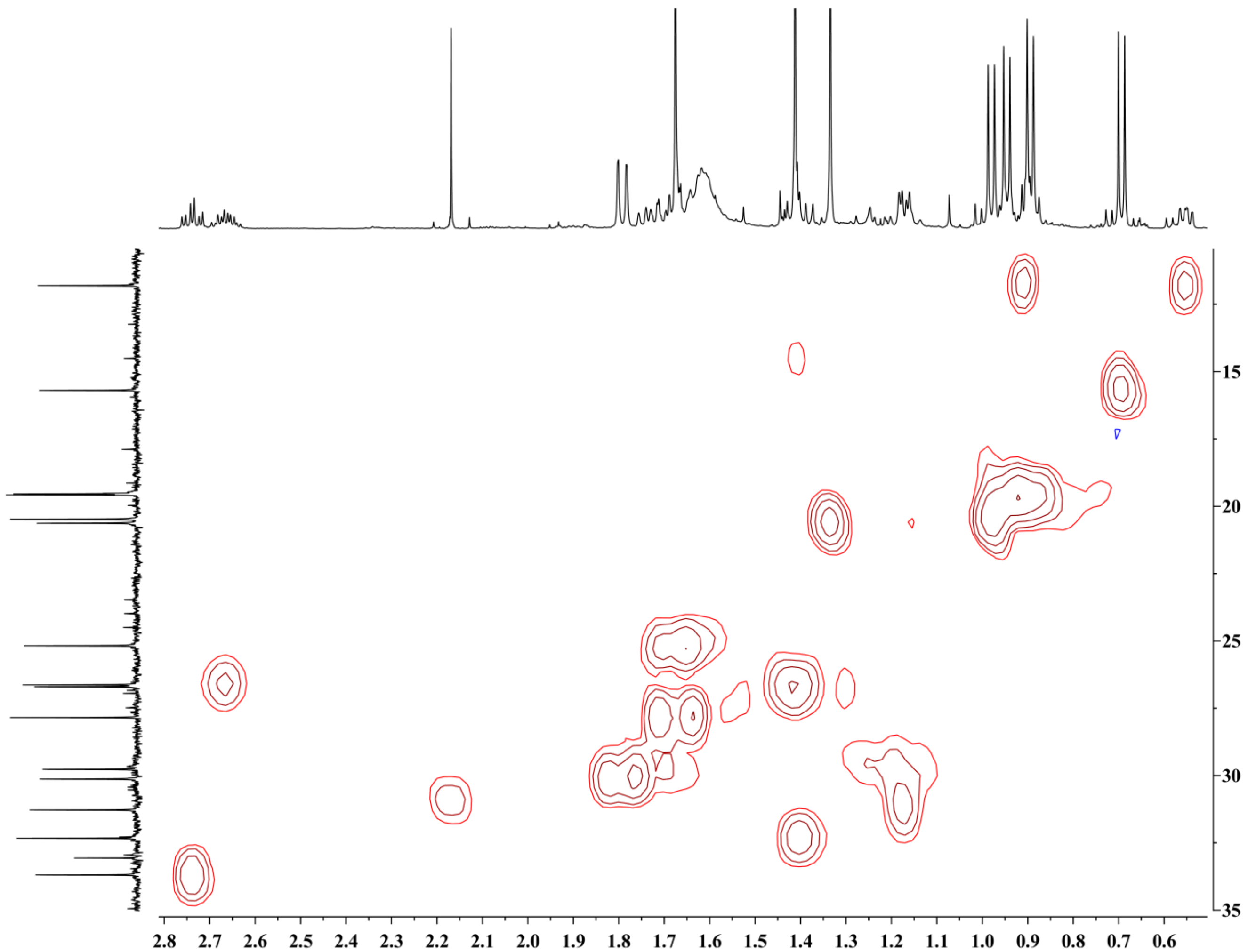

Figure S24. HSQC spectroscopic data of **4**

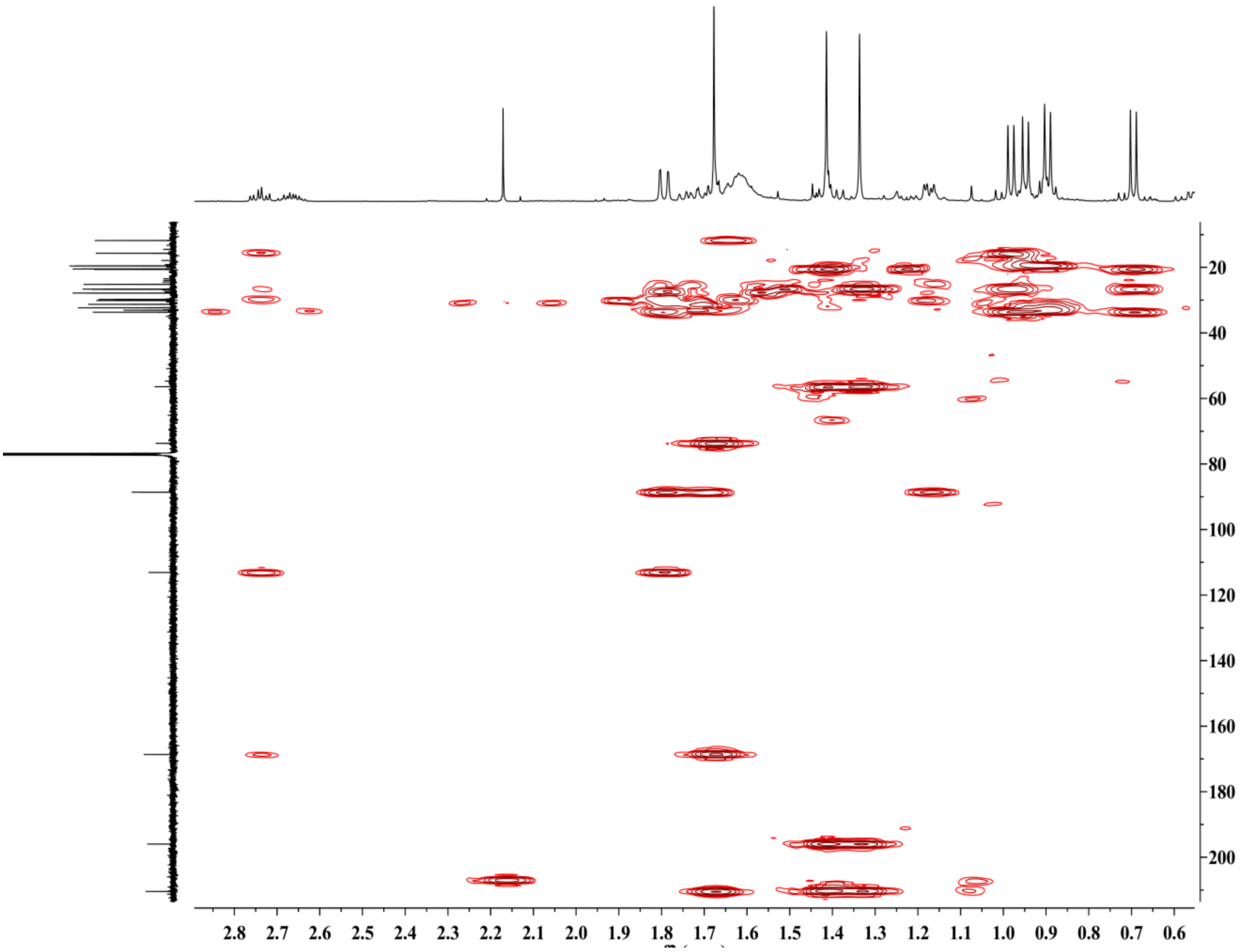

Figure S25. HSBC spectroscopic data of **4**

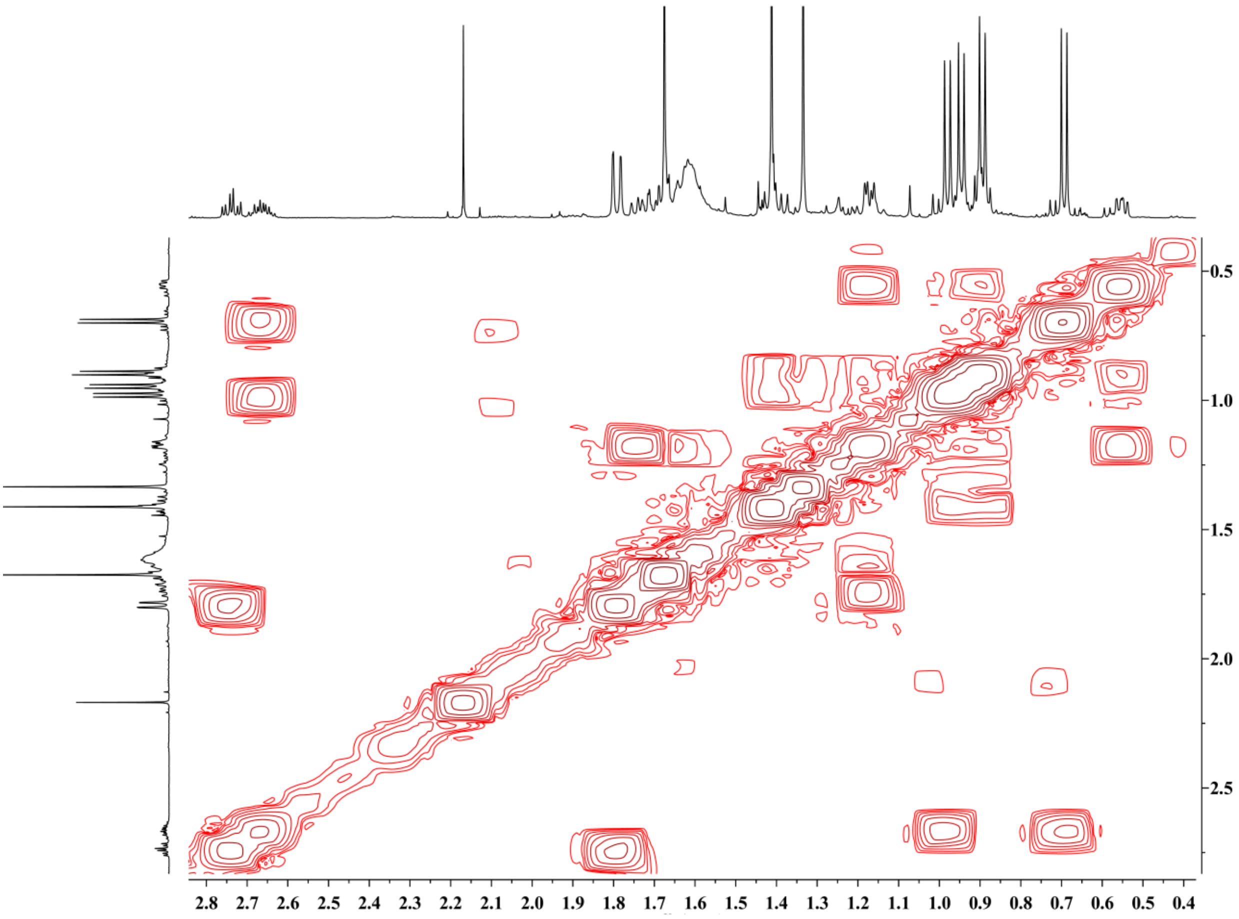

Figure S26. ^{1}H–^{1}HCOSY spectroscopic data of **4**

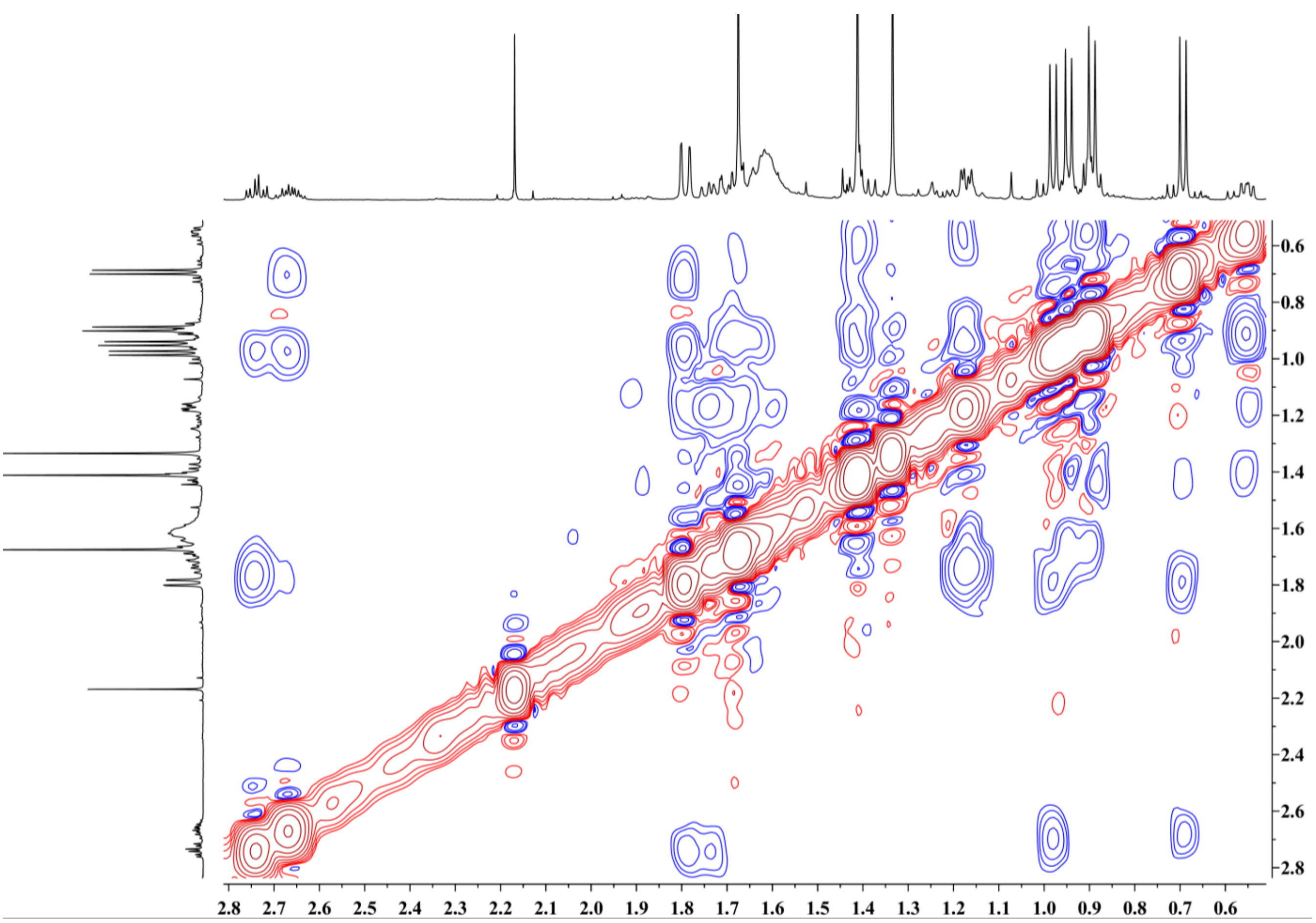

Figure S27. ROESY spectroscopic data of **4**

User Spectra

Fragmentor Voltage	Collision Energy	Ionization Mode
250	0	ESI

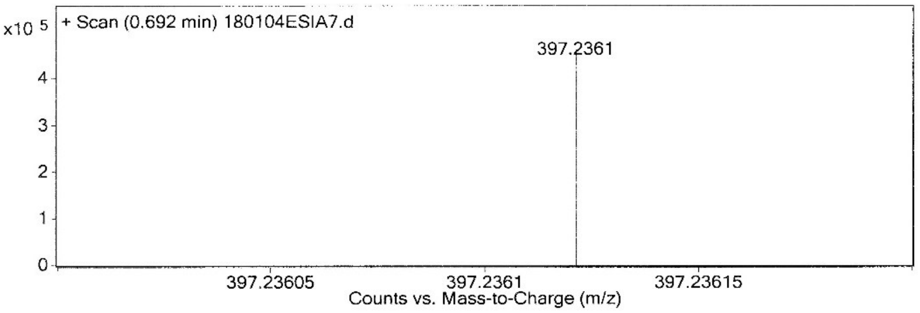

Peak List

m/z	z	Abund	Formula	Ion
121.0509	1	14231.64		
261.1098	1	98019.95		
397.2361	1	460401.75	C23 H34 Na O4	M+
398.2389	1	96930.02	C23 H34 Na O4	M+
409.2349	1	16257.62		
413.2507	1	22215.75		
771.4822	1	140942.3		
772.485	1	61547.04		
773.4864	1	12093.4		
922.0098	1	48289.07		

Formula Calculator Element Limits

Element	Min	Max
C	0	200
H	0	400
O	0	10
Na	1	1

Formula Calculator Results

Formula	CalculatedMass	Mz	Diff.(mDa)	Diff. (ppm)	DBE
C23 H34 Na O4	397.2355	397.2361	-0.6	1.6	6.5

Figure S28. HRESIMS spectroscopic data of **4**

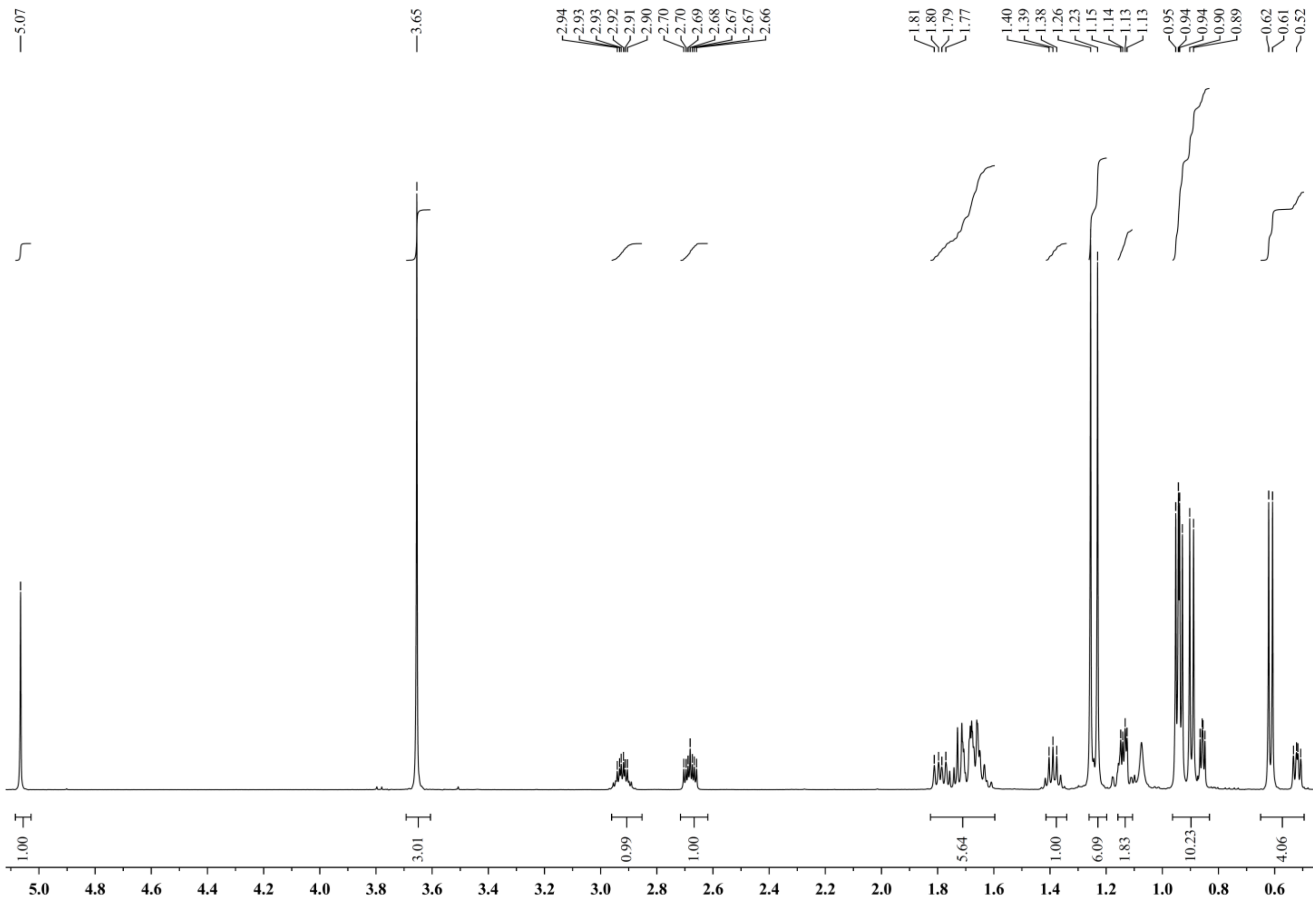

Figure S29. ^{1}H NMR spectroscopic data of **5**

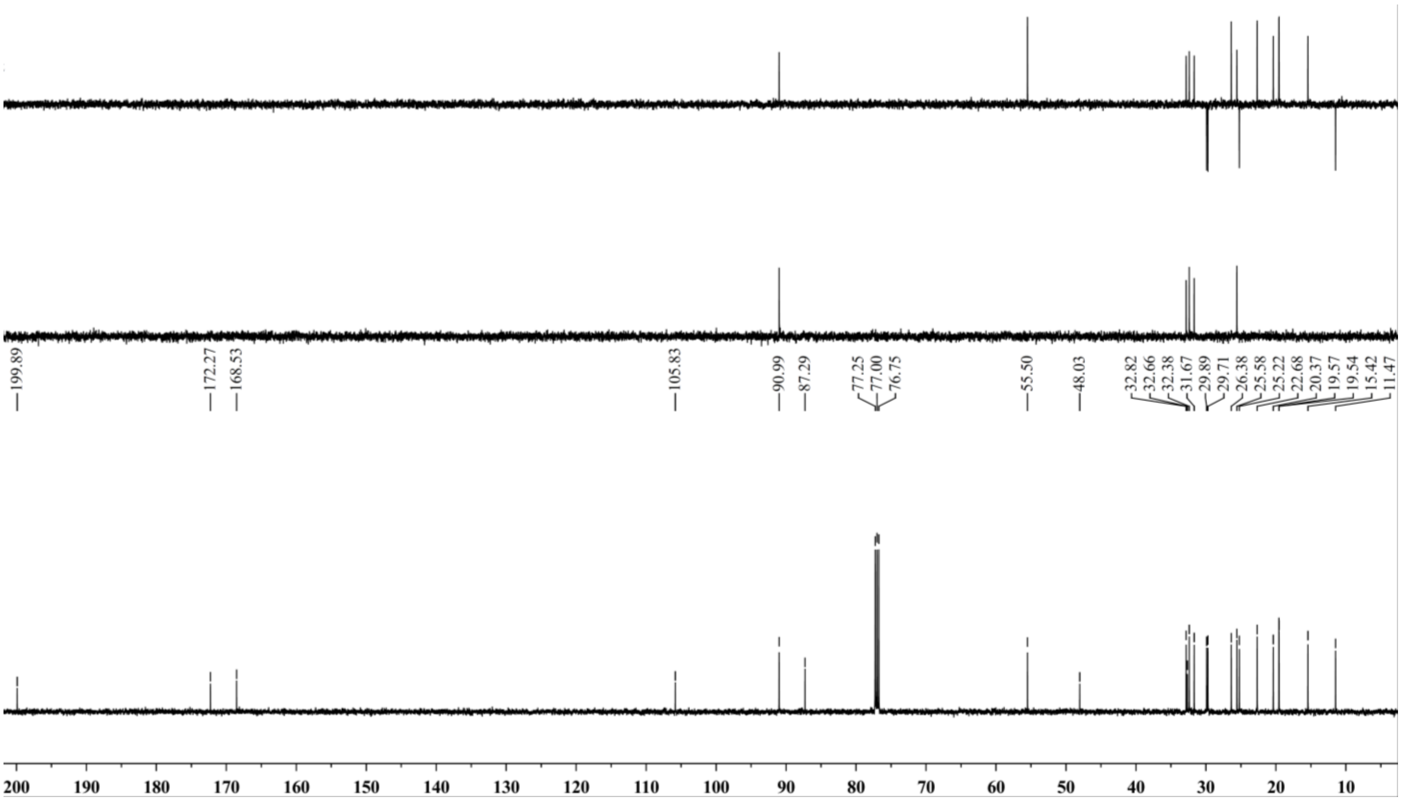

Figure S30. ^{13}C NMR spectroscopic data of **5**

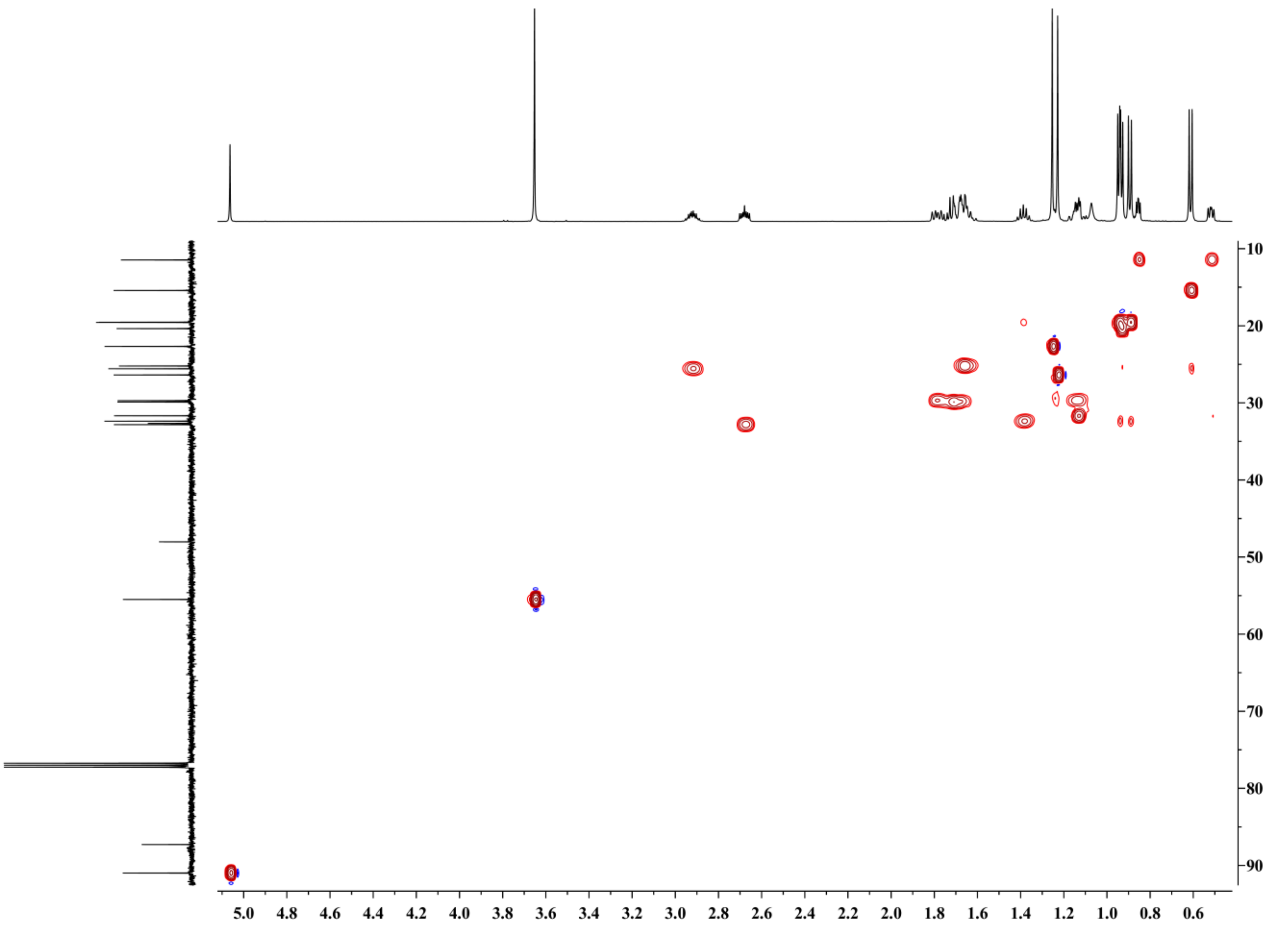

Figure S31. HSQC spectroscopic data of **5**

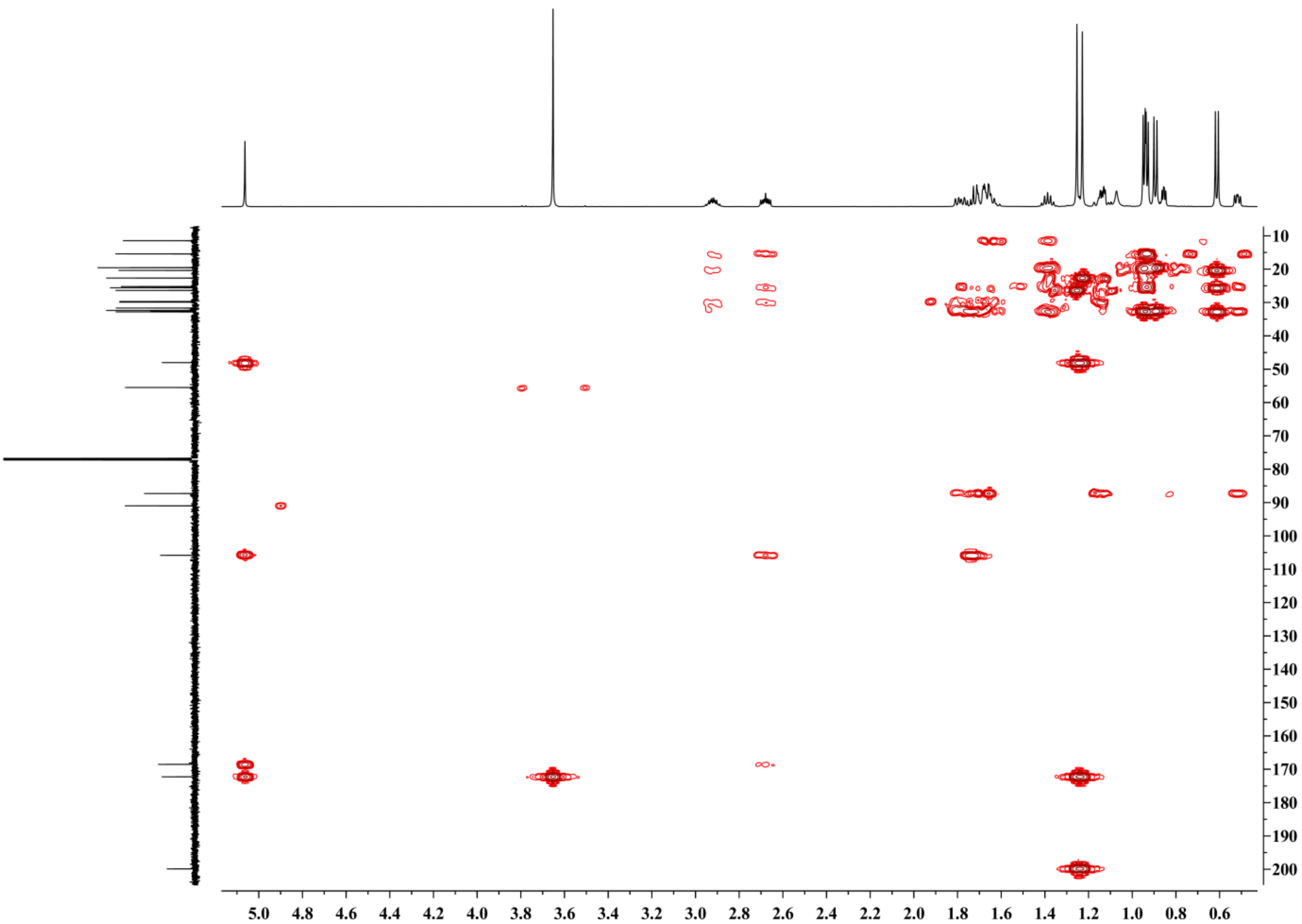

Figure S32. HMBC spectroscopic data of **5**

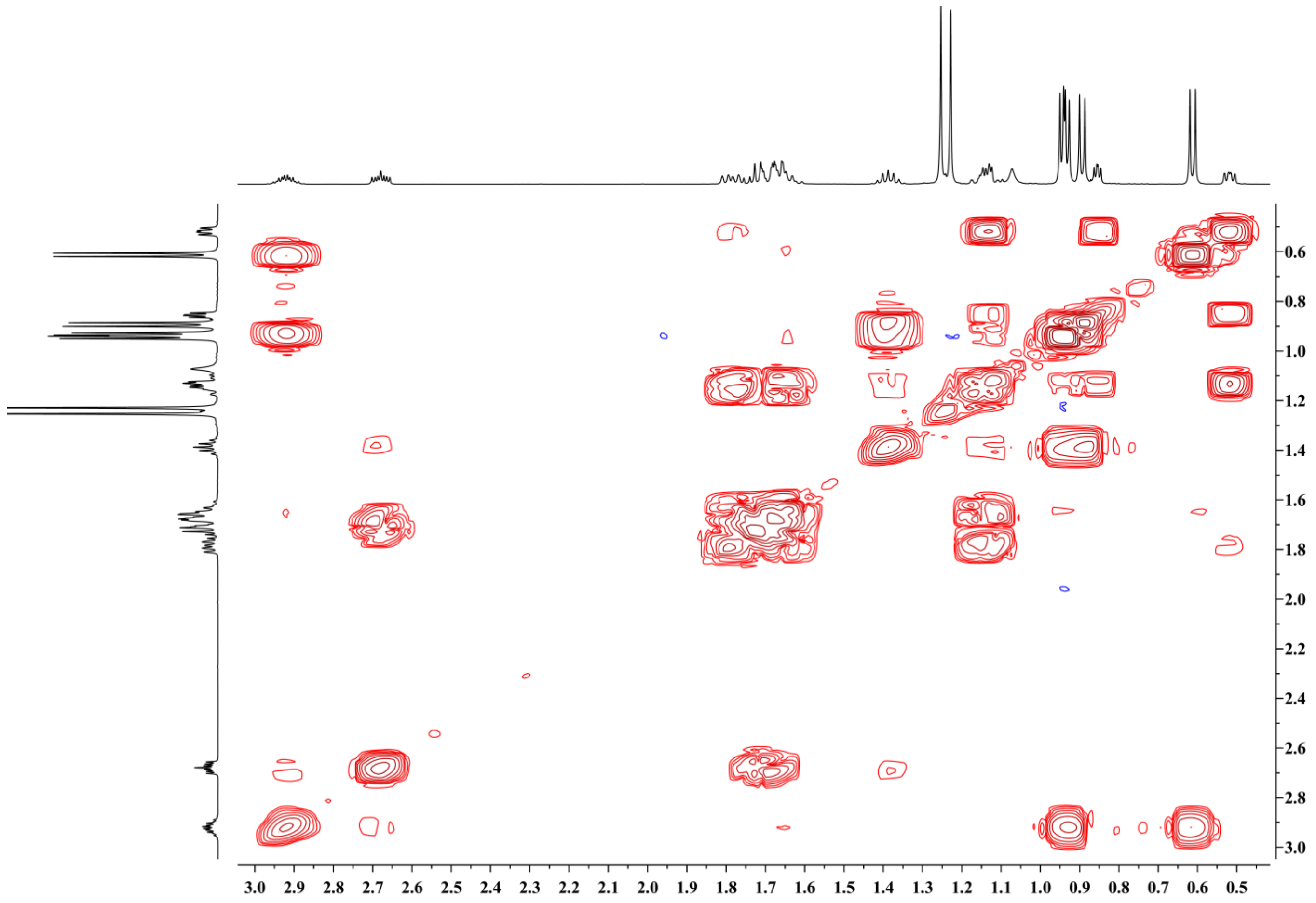

Figure S33. ^{1}H–^{1}H COSY spectroscopic data of **5**

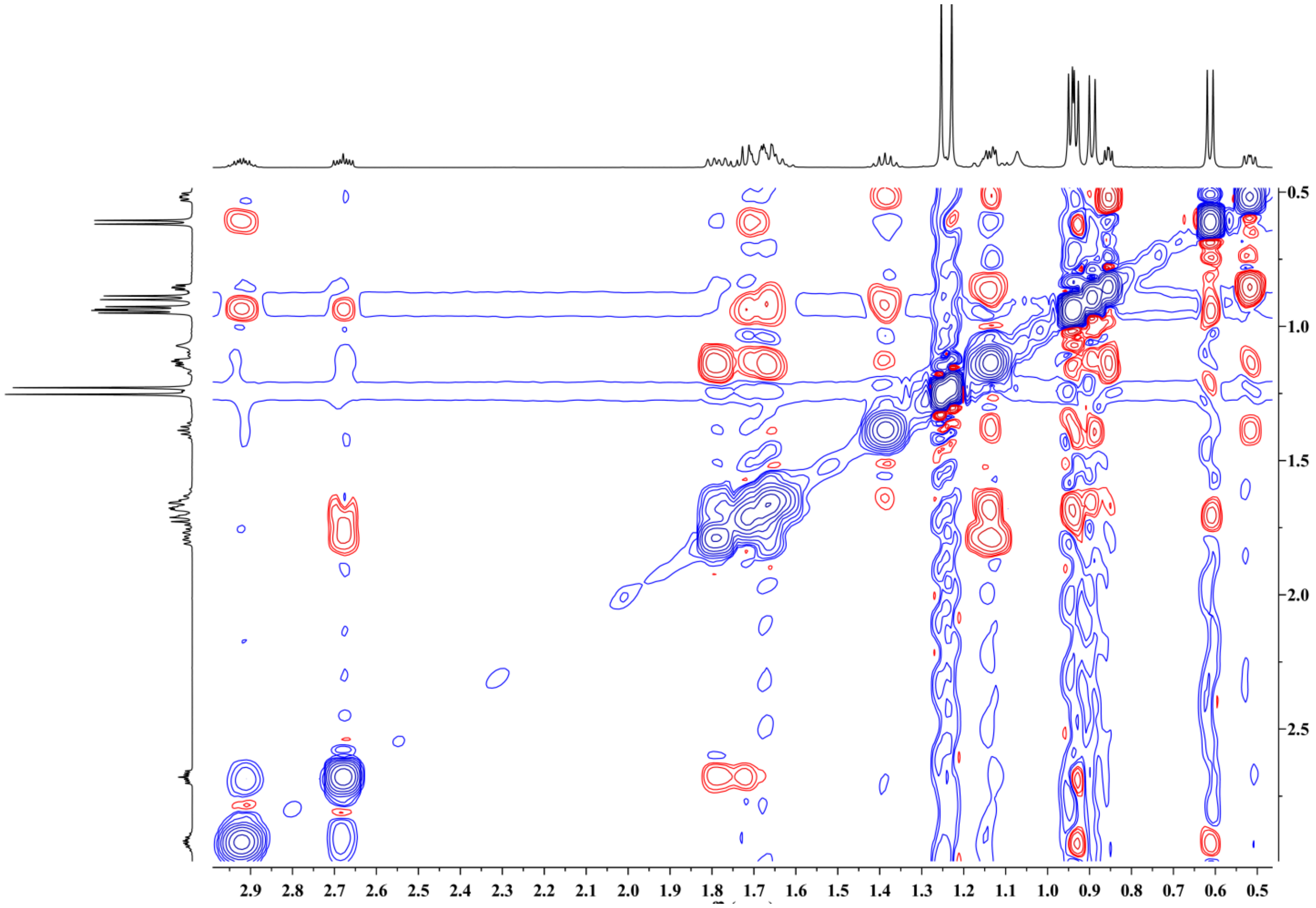

Figure S34. ROESY spectroscopic data of **5**

User Spectra

Fragmentor Voltage	Collision Energy	Ionization Mode
135	0	ESI

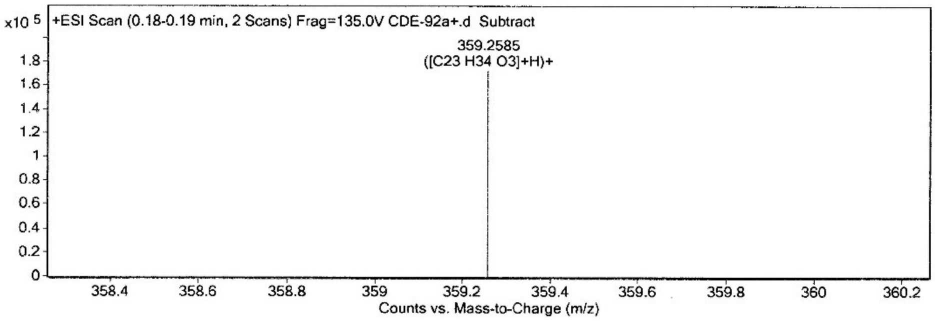

Peak List

m/z	z	Abund	Formula	Ion
359.2585	1	173271.5	C23 H34 O3	(M+H)+
360.2617	1	35876.04	C23 H34 O3	(M+H)+
381.2402	1	112206.63		
382.2433	1	25162.89		
397.2138	1	32706.47		
739.4913	1	88705.5		
740.4943	1	38955.83		
741.4964	1	7755.03		

Formula Calculator Element Limits

Element	Min	Max
C	3	60
H	0	120
O	0	30

Formula Calculator Results

Formula	CalculatedMass	CalculatedMz	Mz	Diff. (mDa)	Diff. (ppm)	DBE
C23 H34 O3	358.2508	359.2581	359.2585	-0.40	-1.11	7.0000

Figure S35. HRESIMS spectroscopic data of **5**

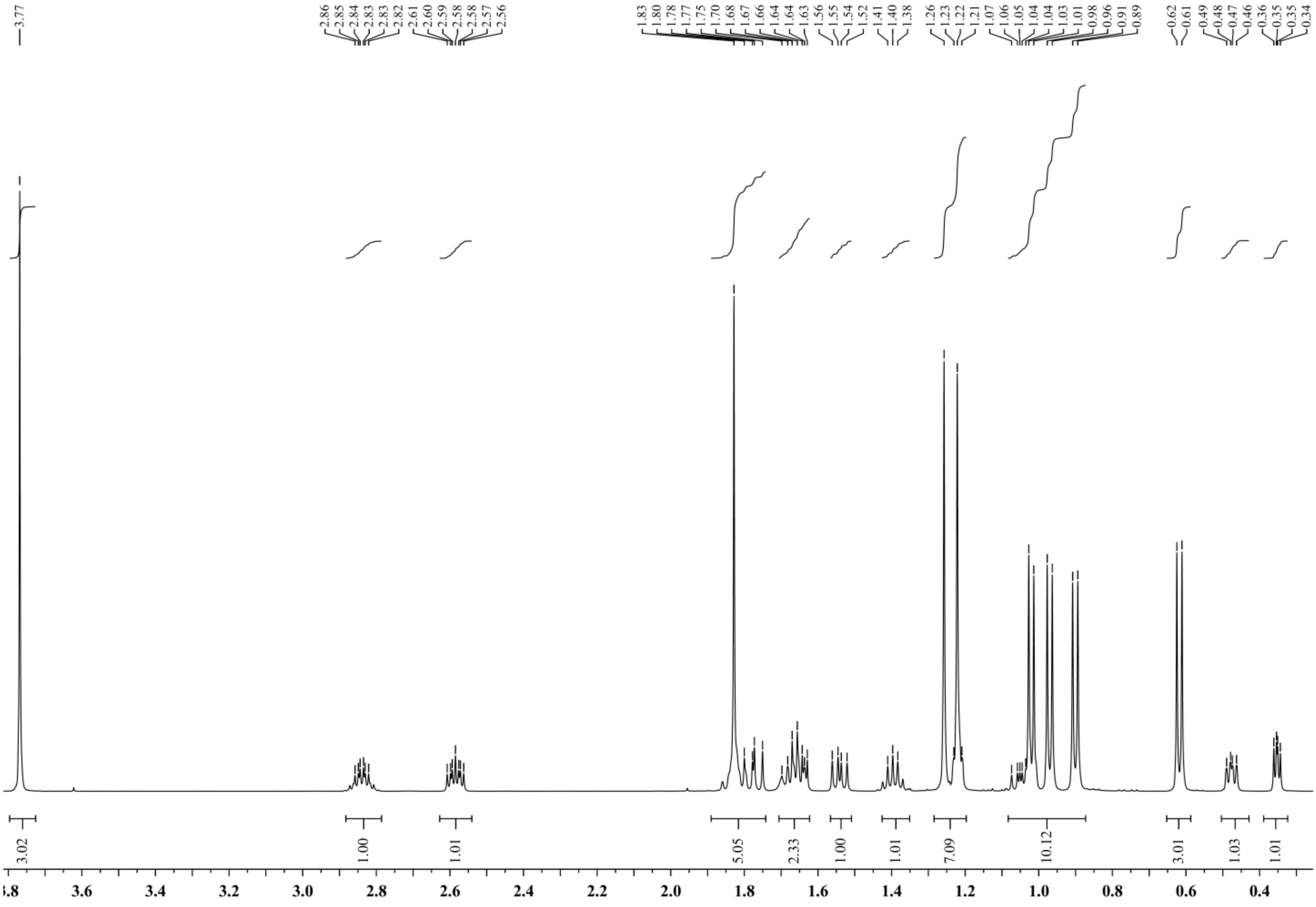

Figure S36. ^{1}H NMR spectroscopic data of **6**

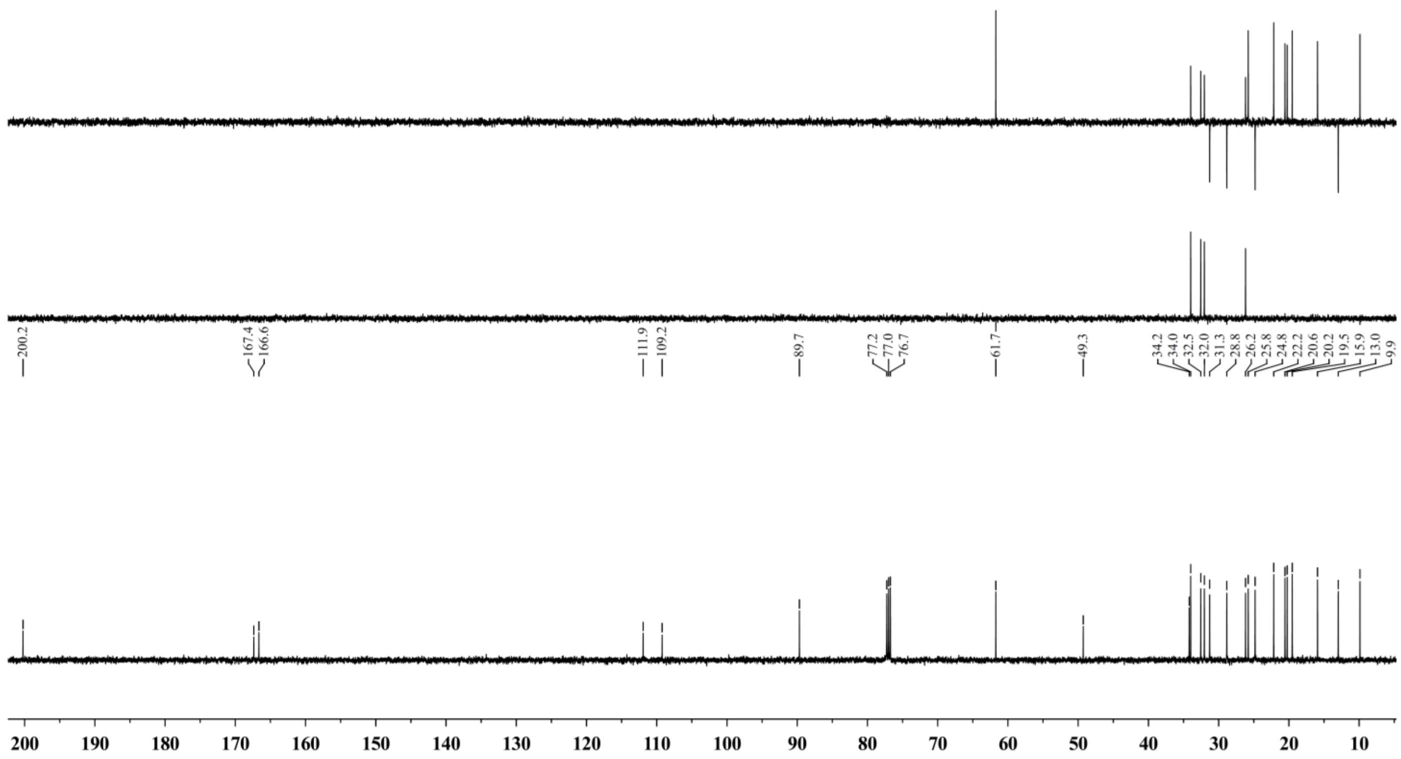

Figure S37. ^{13}C NMR spectroscopic data of **6**

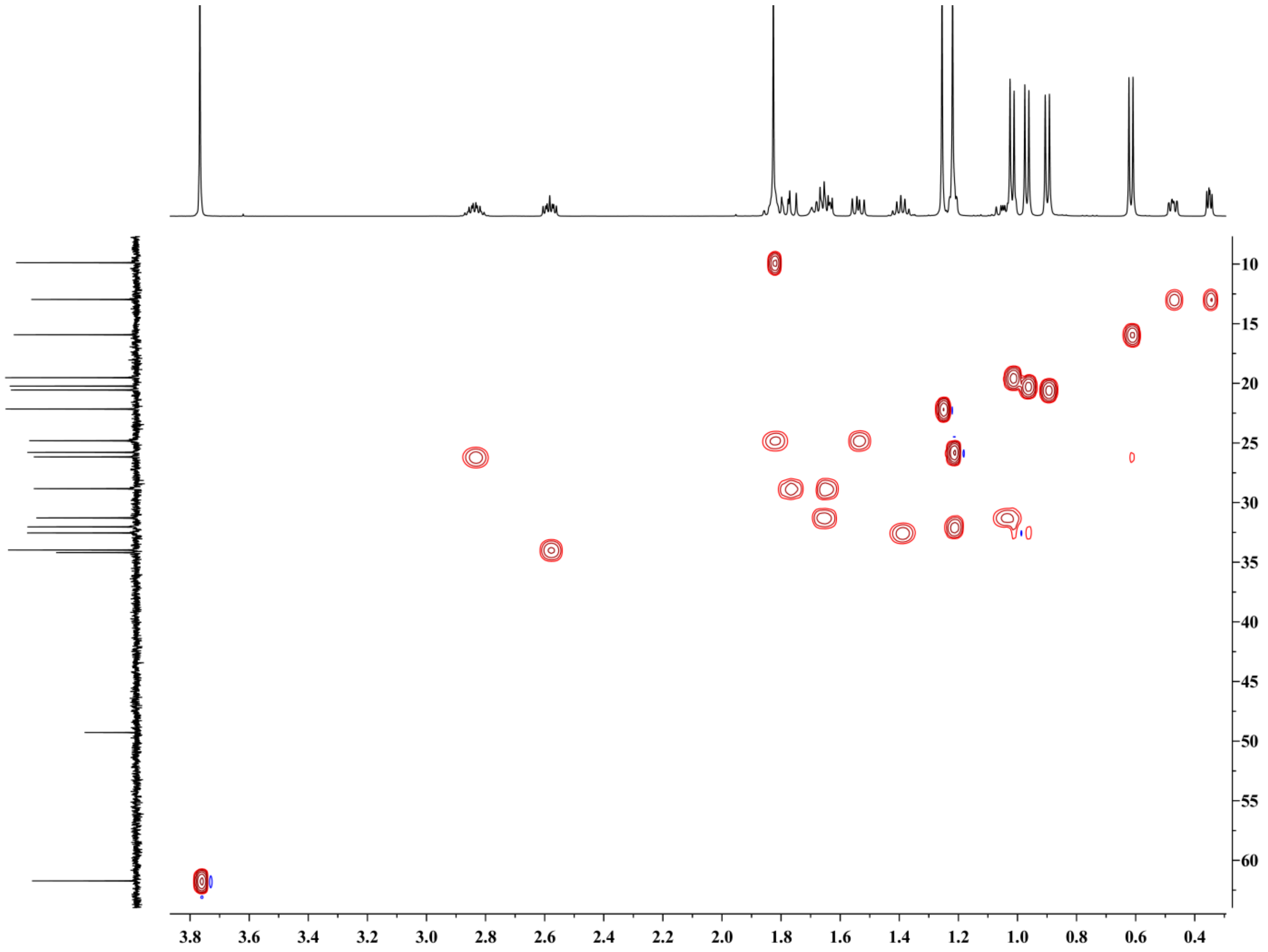

Figure S38. HSQC spectroscopic data of **6**

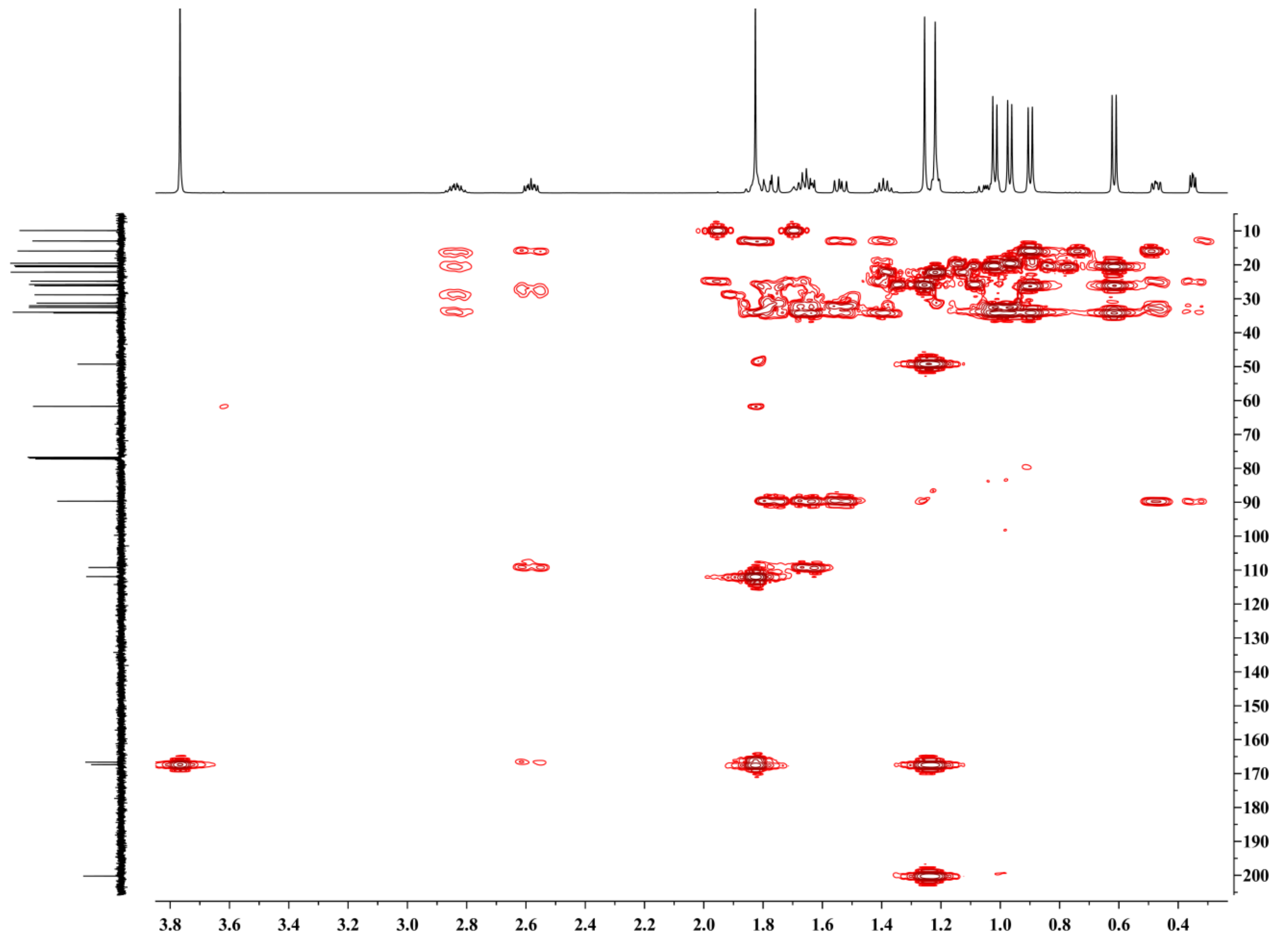

Figure S39. HMBC spectroscopic data of **6**

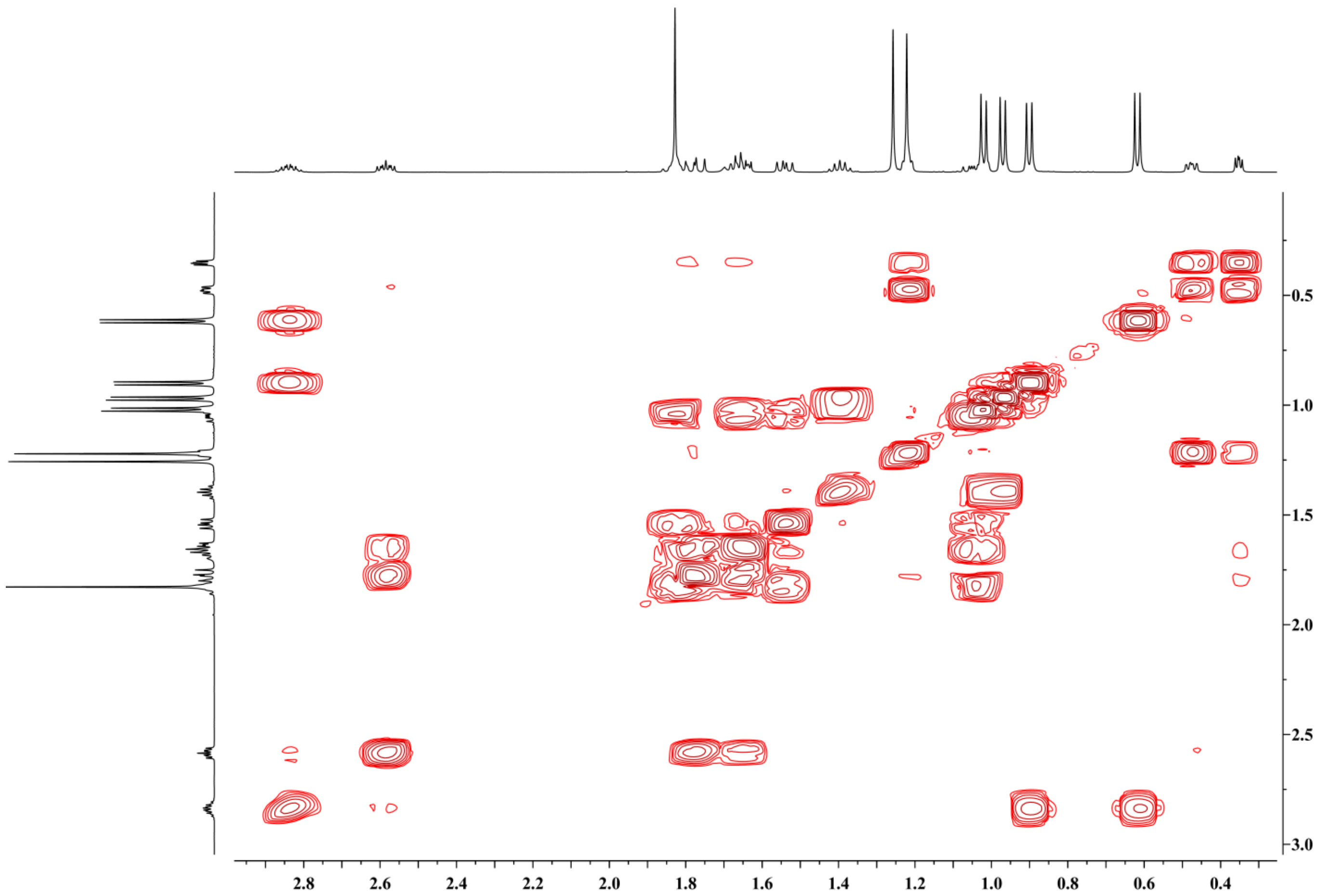

Figure S40. ^{1}H–^{1}H COSY spectroscopic data of **6**

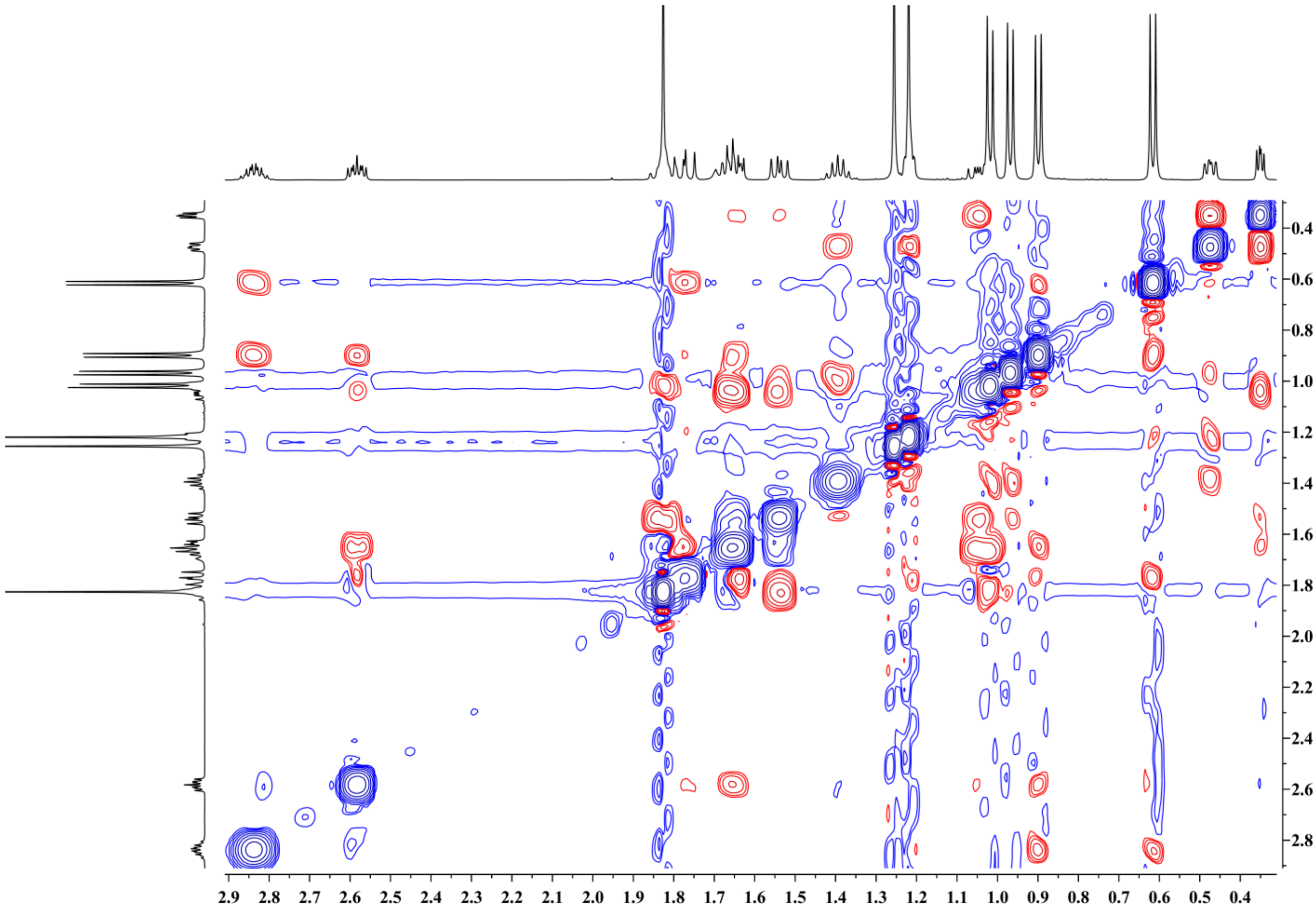

Figure S41. ROESY spectroscopic data of **6**

User Spectra

Fragmentor Voltage	Collision Energy	Ionization Mode
250	0	ESI

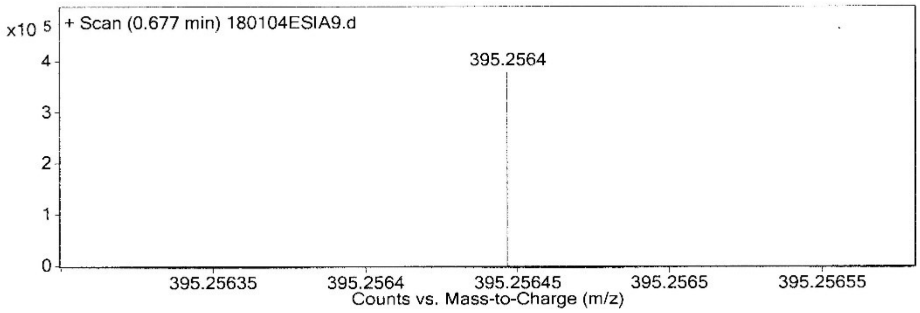

Peak List

m/z	z	Abund	Formula	Ion
121.0509	1	14713.36		
237.148	1	60623.76		
373.2733	1	30692.28		
395.2564	1	380733.59	C24 H36 Na O3	M+
396.2592	1	84082.66	C24 H36 Na O3	M+
411.2288	1	21657.2		
767.5238	1	357465.16		
768.5267	1	173940.91		
769.5292	1	37969.08		
922.0098	1	50301.55		

Formula Calculator Element Limits

Element	Min	Max
C	0	200
H	0	400
O	0	10
Na	1	1

Formula Calculator Results

Formula	CalculatedMass	Mz	Diff.(mDa)	Diff. (ppm)	DBE
C24 H36 Na O3	395.2562	395.2564	-0.2	0.5	6.5

Figure S42. HRESIMS spectroscopic data of **6**

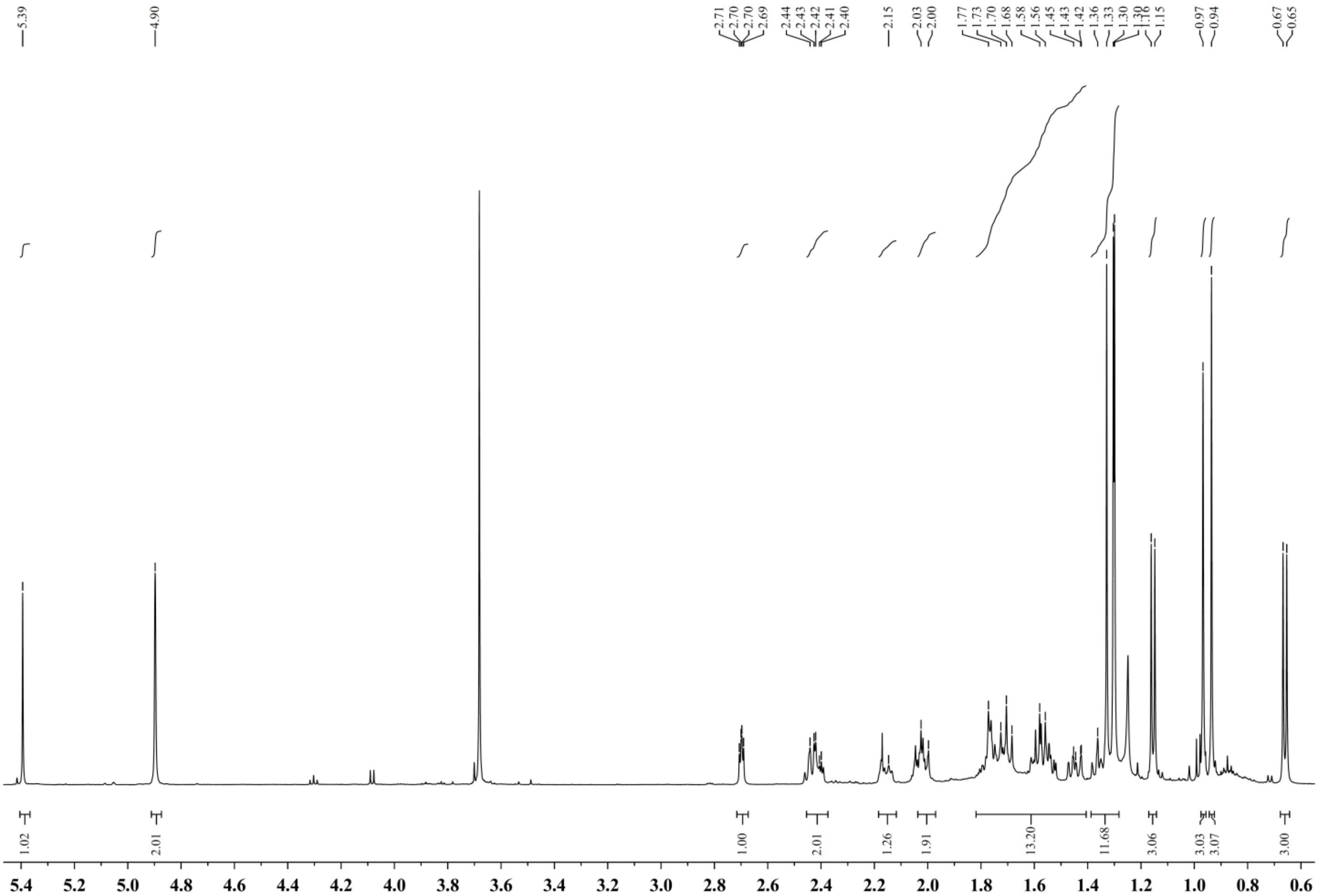

Figure S43. ^{1}H NMR spectroscopic data of **7**

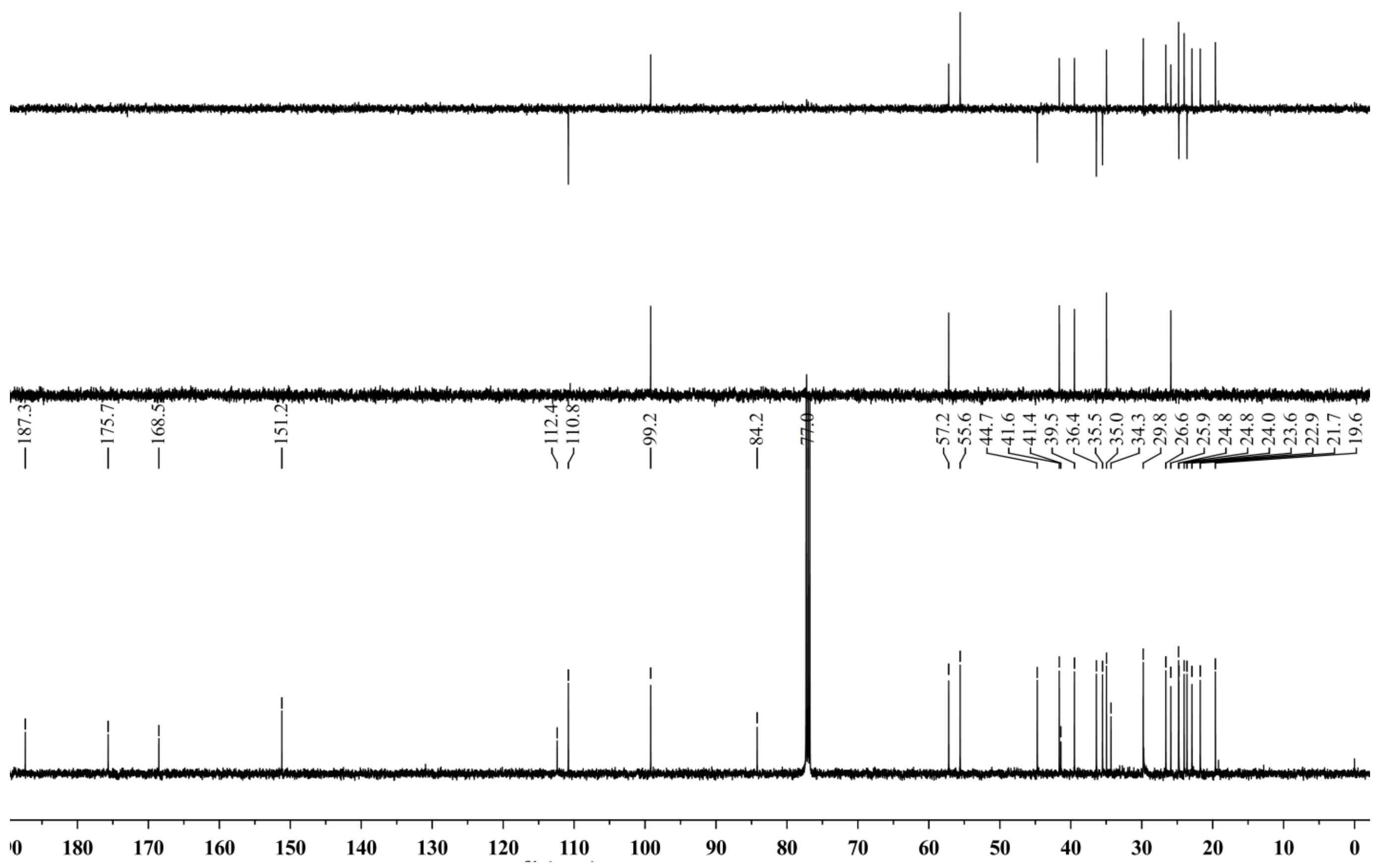

Figure S44. ^{13}C NMR spectroscopic data of **7**

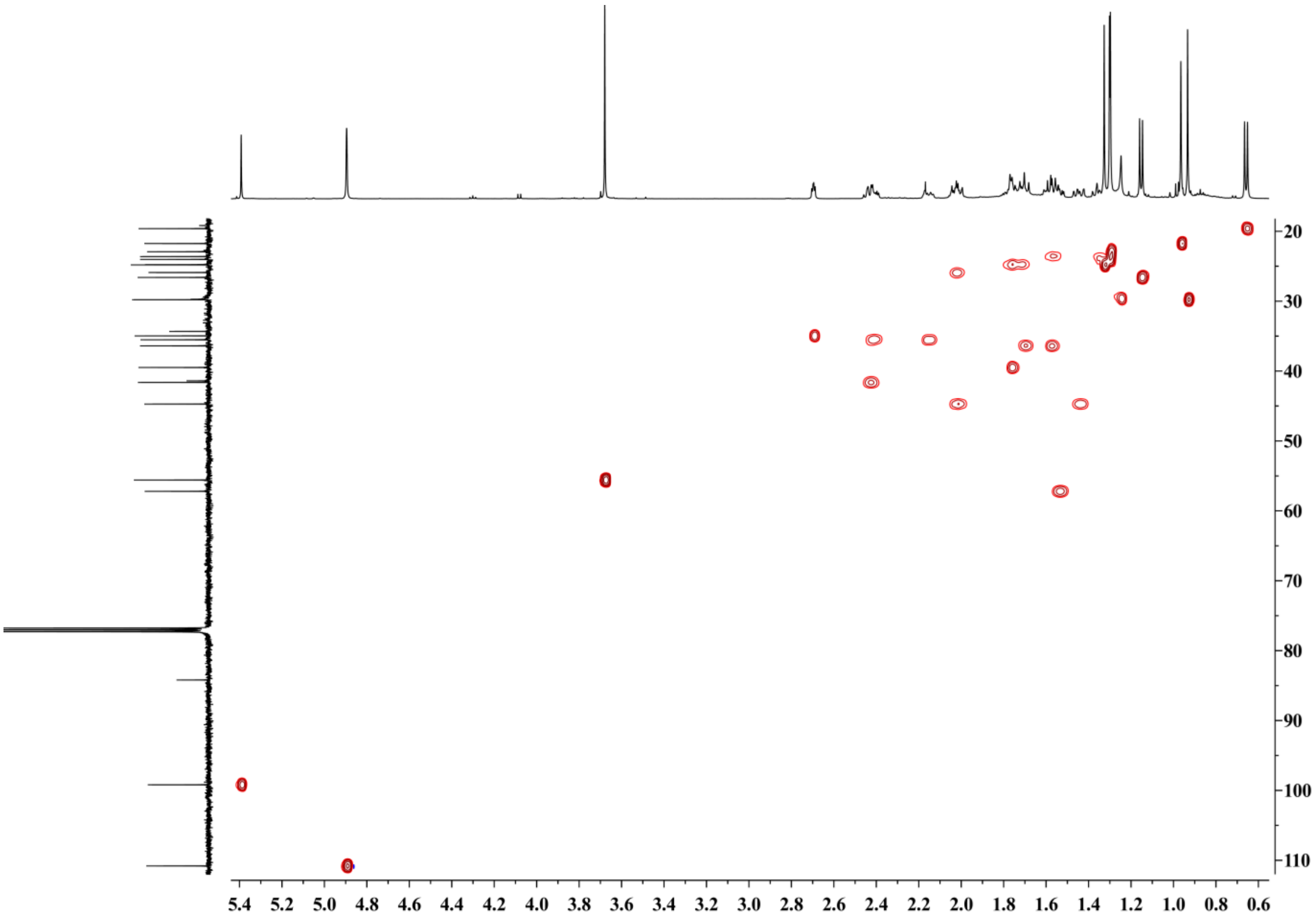

Figure S45. HSQC spectroscopic data of **7**

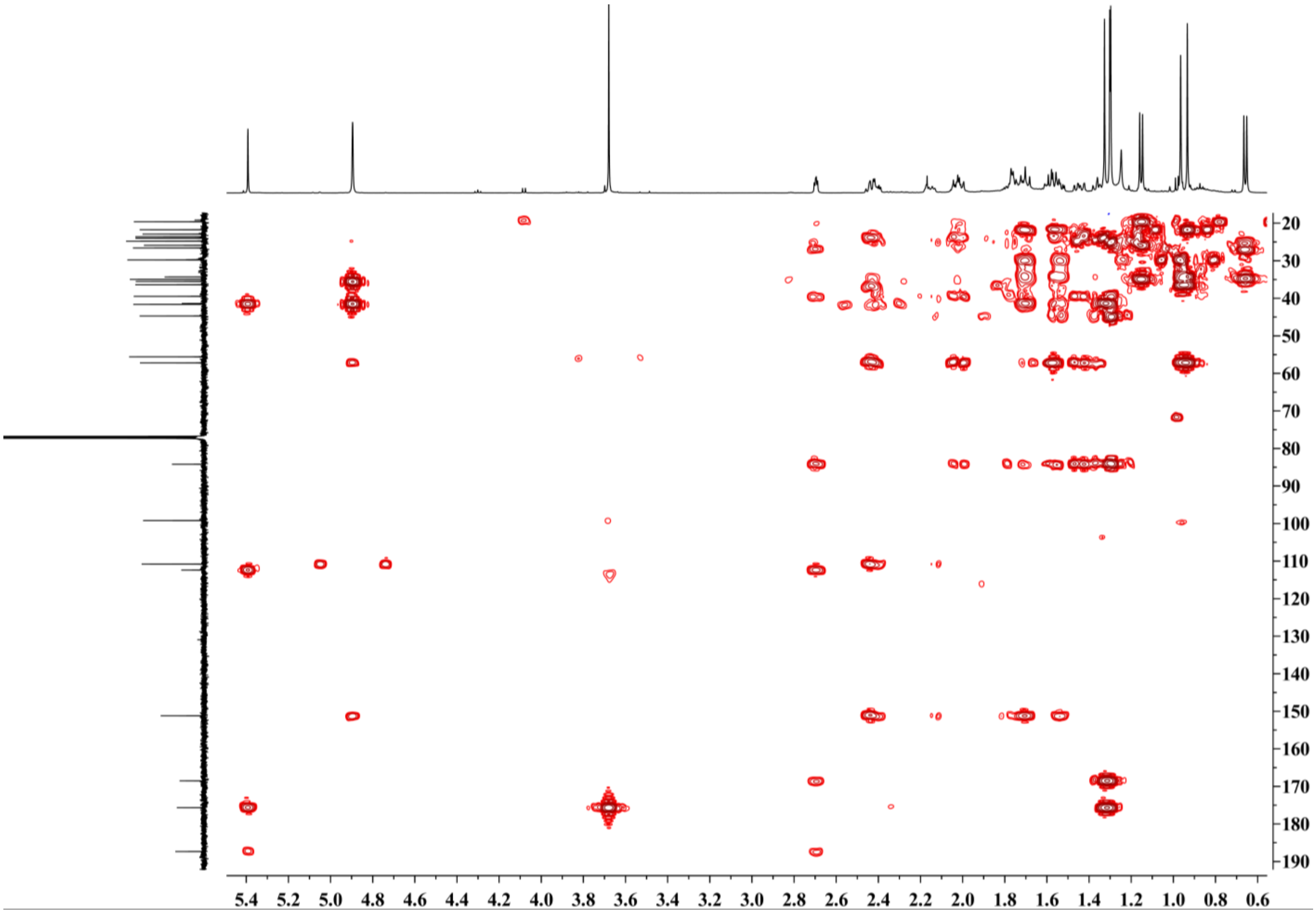

Figure S46. HMBC spectroscopic data of **7**

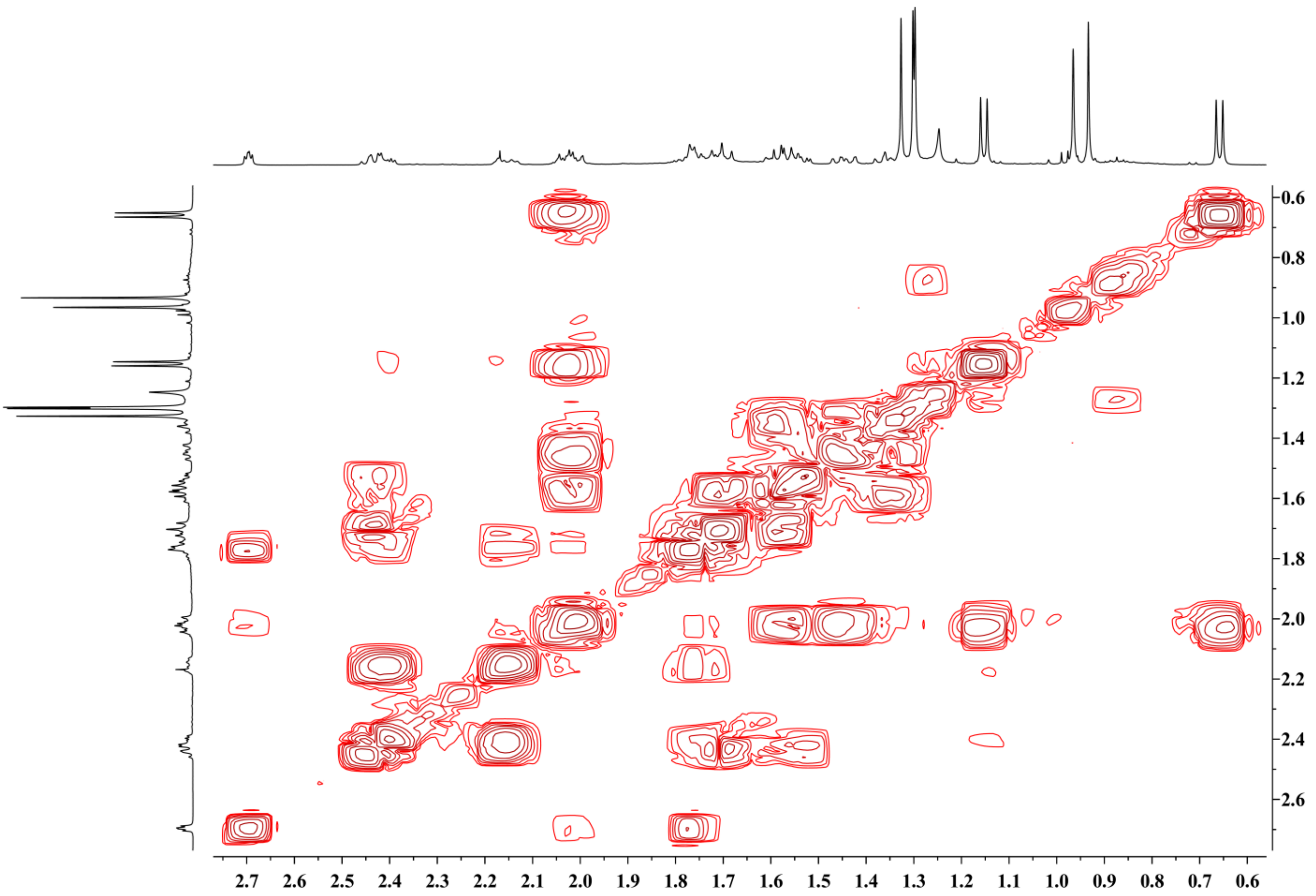

Figure S47. ^{1}H–^{1}H COSY spectroscopic data of **7**

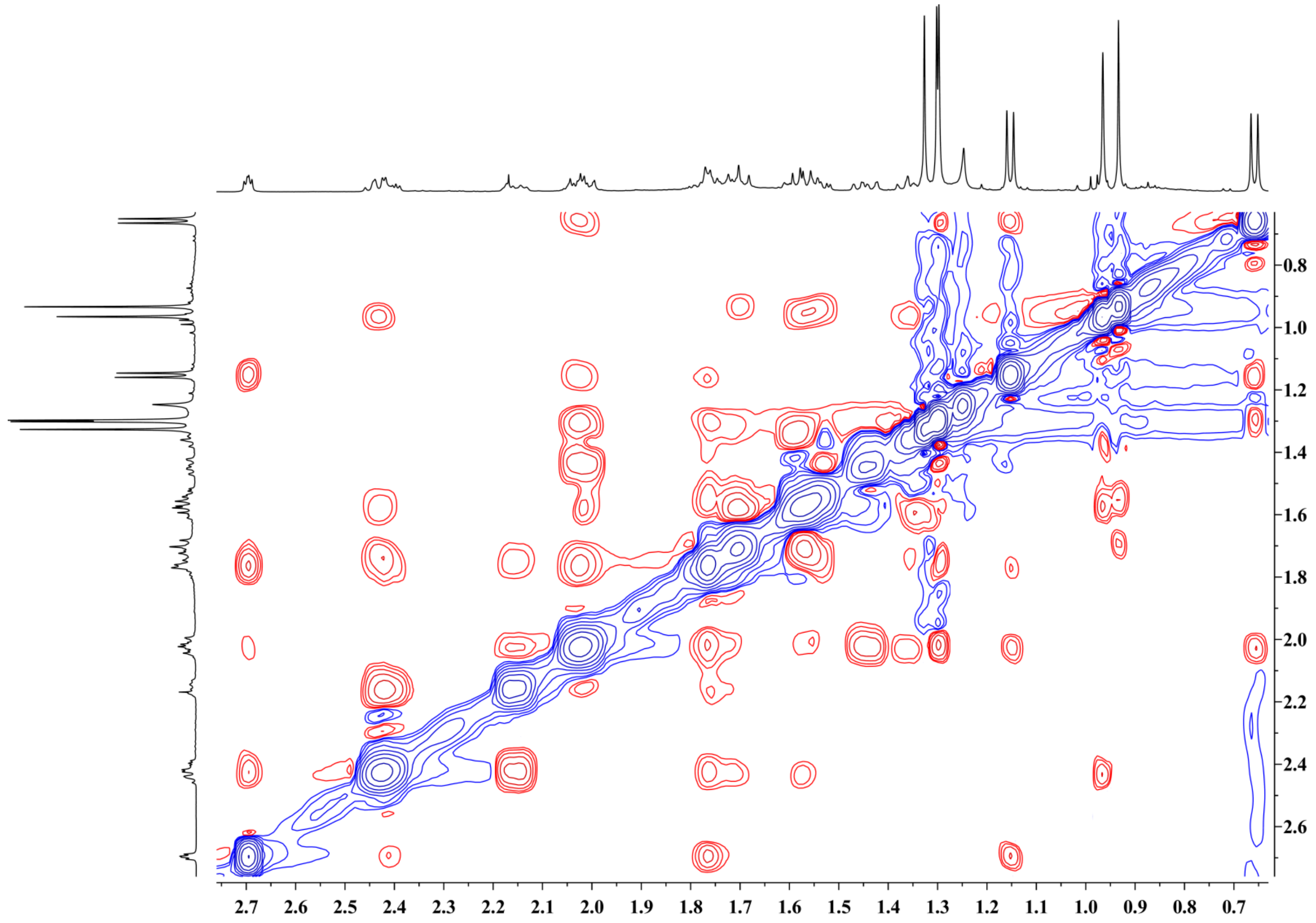

Figure S48. ROESY spectroscopic data of **7**

User Spectra

Fragmentor Voltage	Collision Energy	Ionization Mode
250	0	ESI

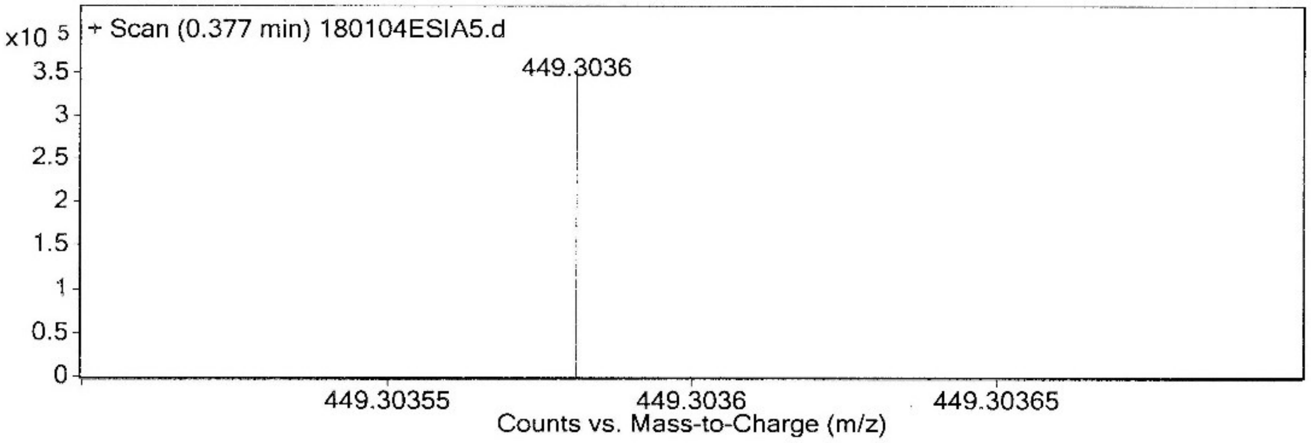

Peak List

m/z	z	Abund	Formula	Ion
427.3207	1	81050.62		
449.3036	1	352845.38	C28 H42 Na O3	M+
450.3064	1	91561.59	C28 H42 Na O3	M+
465.2954	1	97803.91		
875.6182	1	615653.25		
876.6214	1	369536.59		
877.6213	1	116668.23		
1301.9304	1	225486.97		
1302.9339	1	209104.09		
1303.9367	1	86935.7		

Formula Calculator Element Limits

Element	Min	Max
C	0	200
H	0	400
O	0	10
Na	1	1

Formula Calculator Results

Formula	CalculatedMass	Mz	Diff.(mDa)	Diff. (ppm)	DBE
C28 H42 Na O3	449.3032	449.3036	-0.4	1.0	7.5

Figure S49. HRESIMS spectroscopic data of **7**

Computational data of 1–5 and 7

Computational data of **1**-7′*S*,1′*R*,2′*S*,4′*R*,6′*R*

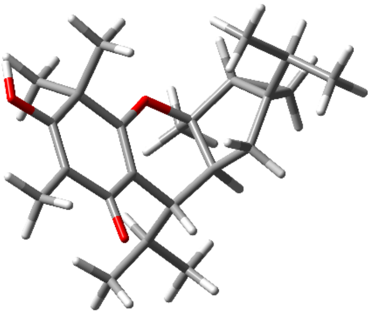

Conf. 1
Distribution = 52.94%
ΔE (kcal/mol) = 0.00

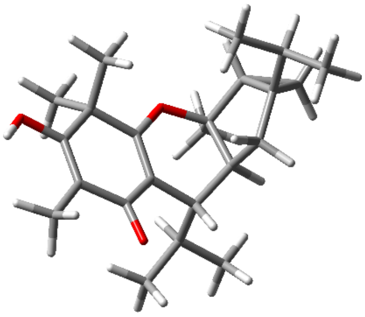

Conf. 2
Distribution = 36.77%
ΔE (kcal/mol) = 0.22

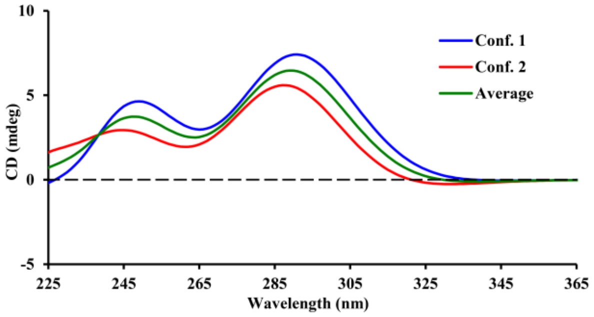

Computational data of **2**-7*R*,1′*S*,2′*S*,4′*R*

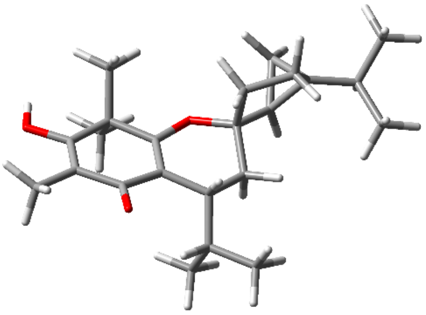

Conf. 1
Distribution = 32.58%
ΔE (kcal/mol) = 0.00

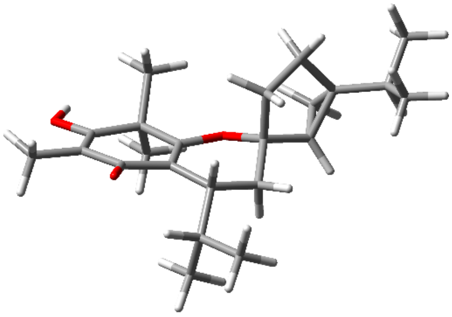

Conf. 2
Distribution = 24.12%
ΔE (kcal/mol) = 0.18

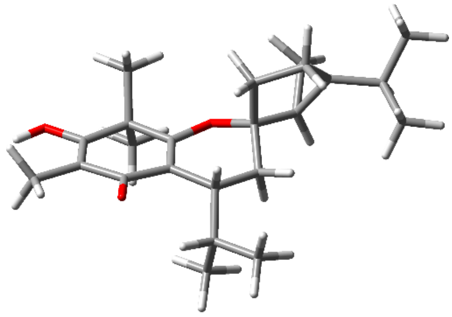

Conf. 3
Distribution = 22.67%
ΔE (kcal/mol) = 0.22

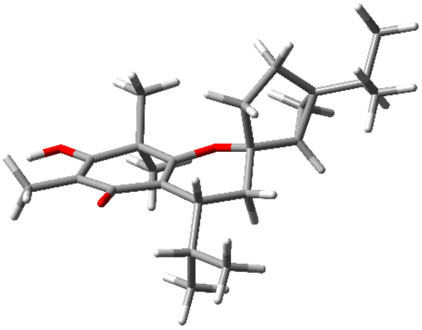

Conf. 4
Distribution = 17.51%
ΔE (kcal/mol) = 0.37

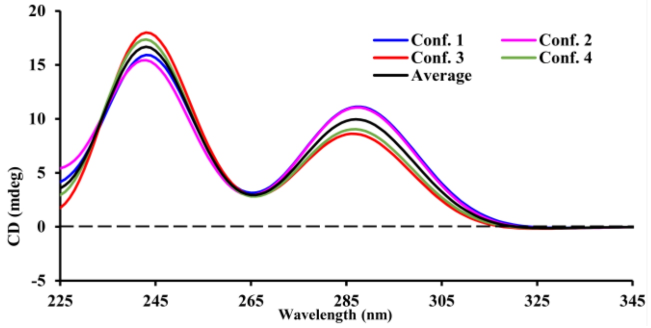

Computational data of **3**-5*R*,7*R*,1′*R*,2′*S*,4′*S*

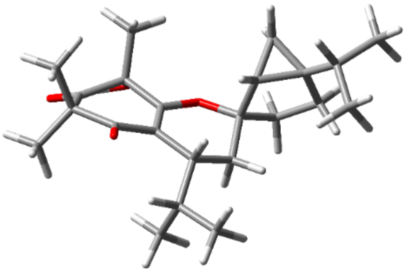

Conf. 1
Distribution = 50.50%
ΔE (kcal/mol) = 0.00

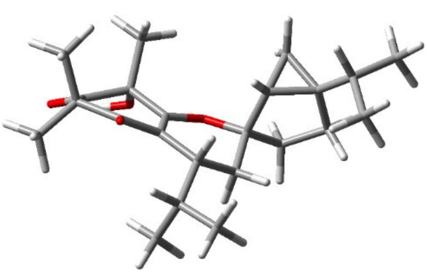

Conf. 2
Distribution = 6.76%
ΔE (kcal/mol) = 0.19

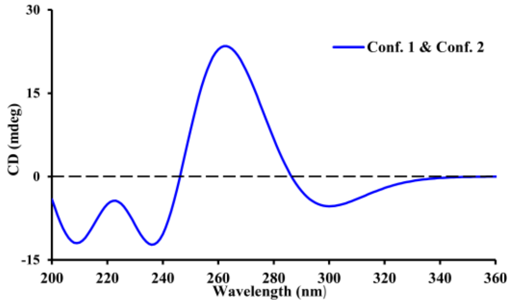

Computational data of **3**-5S,7R,1′R,2′S,4′S

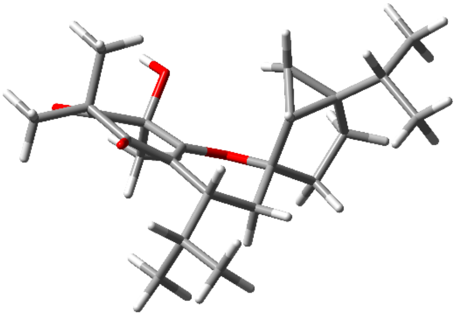

Conf. 1
Distribution = 32.58%
ΔE (kcal/mol) = 0.00

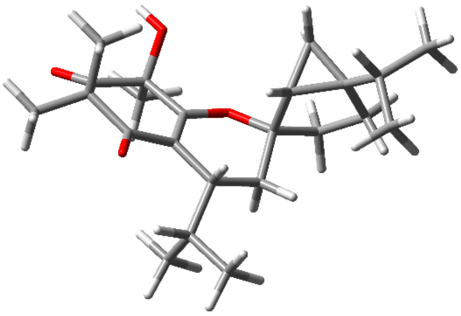

Conf. 2
Distribution = 24.12%
ΔE (kcal/mol) = 0.18

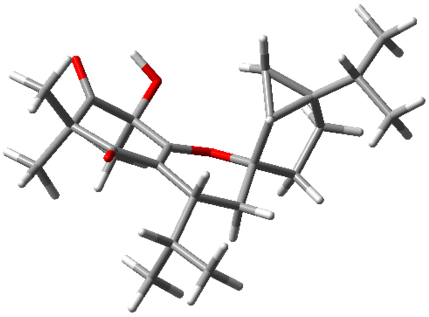

Conf. 3
Distribution = 22.67%
ΔE (kcal/mol) = 0.22

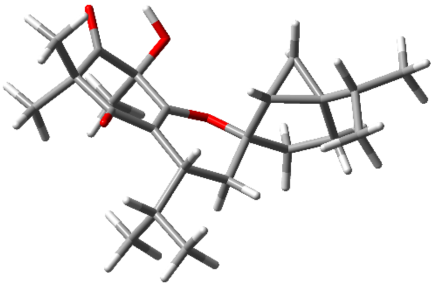

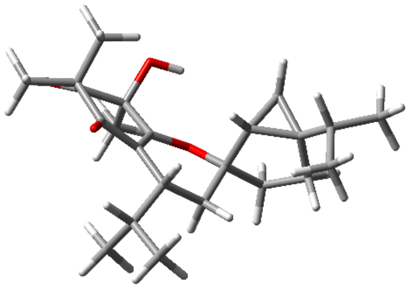

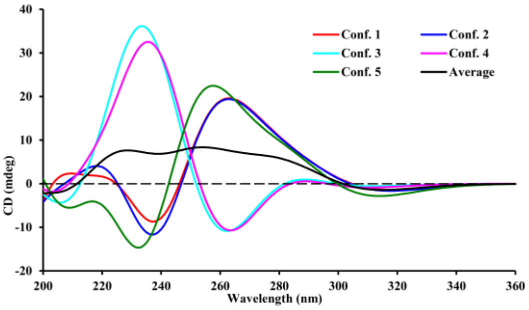

Computational data of **4**-5S,7S,1′R,2′S,4′R

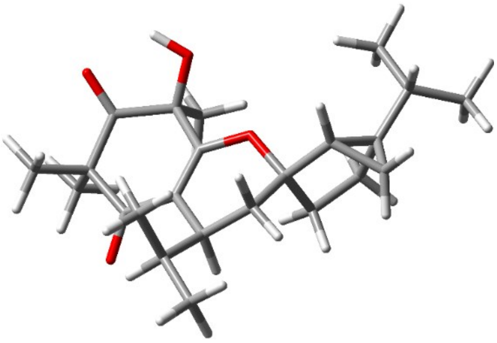

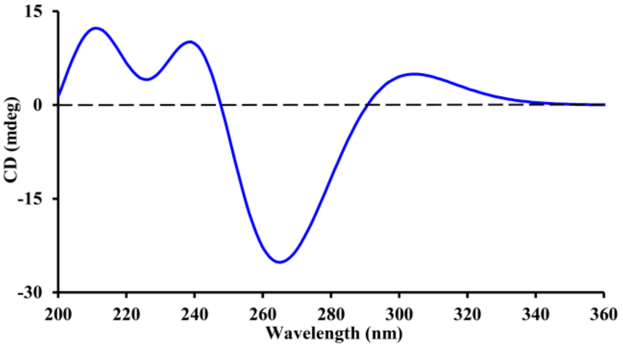

Distribution = 83.98%
ΔE (kcal/mol) = 0.00

Computational data of **4**-5R,7S,1′R,2′S,4′R

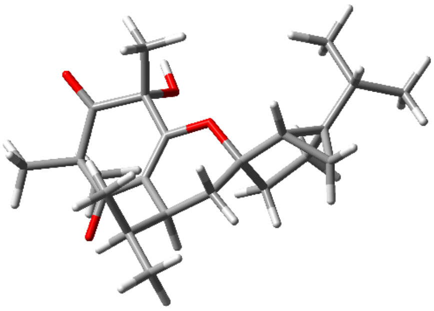

Conf. 1
Distribution = 55.83%
ΔE (kcal/mol) = 0.00

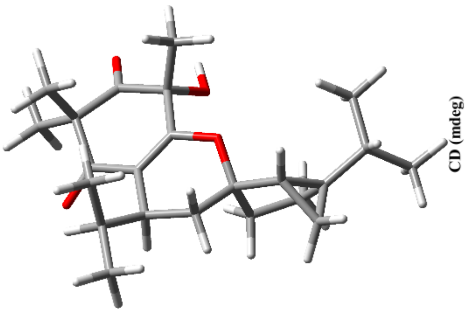

Conf. 2
Distribution = 37.85%
ΔE (kcal/mol) = 0.23

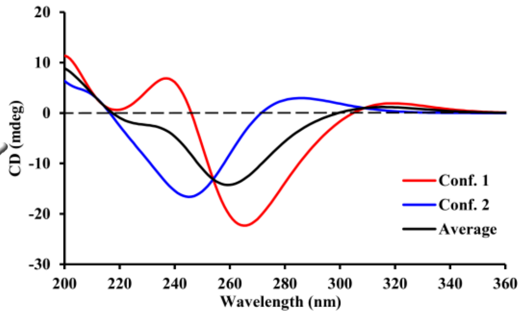

Computational data of **5**-7*S*,1′*R*,2′*R*,4′*S*

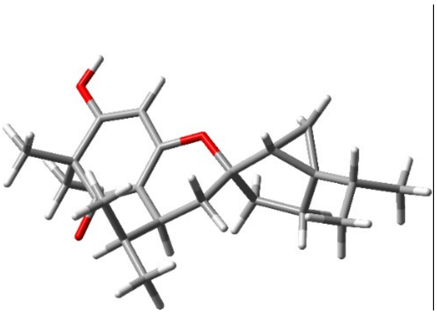

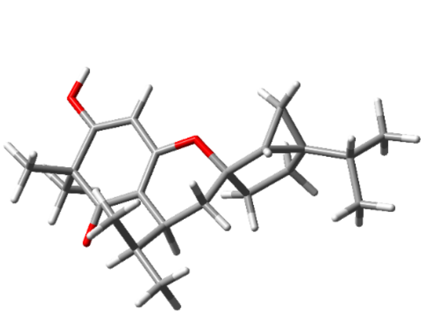

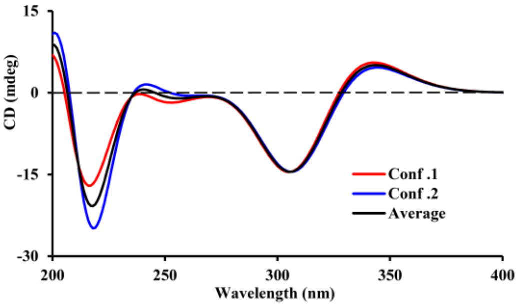

Conf. 1
Distribution = 58.18%
ΔE (kcal/mol) = 0.00

Conf. 2
Distribution = 32.36%
ΔE (kcal/mol) = 0.35

Computational data of **7**-7*S*,1′*R*,2′*R*,4′*S*

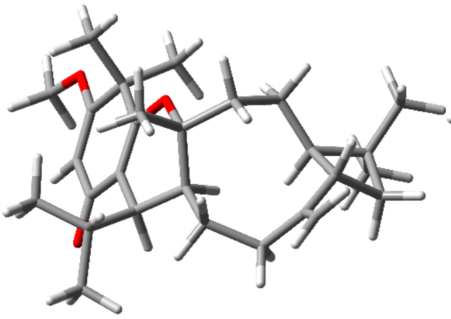

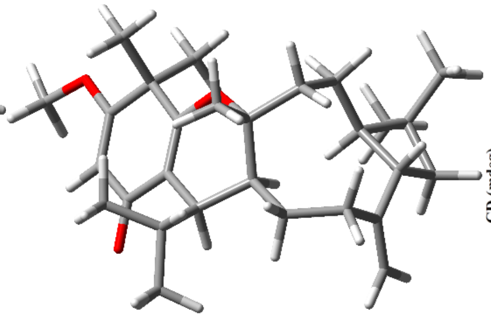

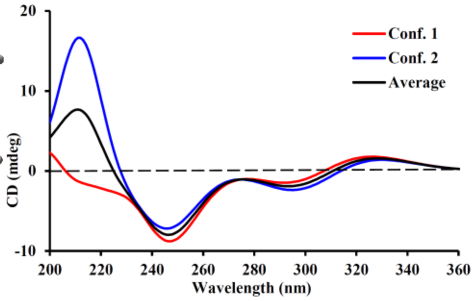

Conf. 1
Distribution = 73.85%
ΔE (kcal/mol) = 0.00

Conf. 2
Distribution = 25.96%
ΔE (kcal/mol) = 0.62
